# Synthesis and Cytotoxic Evaluation of Some Substituted 5-Pyrazolones and Their Urea Derivatives

**DOI:** 10.3390/molecules25040900

**Published:** 2020-02-18

**Authors:** Aliye Gediz Erturk, Hilal Omerustaoglu

**Affiliations:** Department of Chemistry, Faculty of Science & Arts, Ordu University, 52200 Ordu, Turkey; hilal_ustaoglu@hotmail.com

**Keywords:** 5-pyrazolone, urea derivatives, cell proliferation, antioxidant activity

## Abstract

In this paper, a series of new substituted-5-pyrazolones were first synthesized, then formulated by the Vilsmeier–Haack reaction to obtain substituted-4-carbaldehyde-5-pyrazolones. In the final step, when urea was reacted with formulated pyrazolones, we found that, instead of the C=N bond in azomethine form, the compounds tautomerized to form a series of novel pyrazole-4-ylidenemethylurea structures. The structures of these compounds were elucidated by FTIR, ^1^H, ^13^C NMR, LC-MS/MS, and elemental analysis methods. The cytotoxic and antioxidant effects of substituted 5-pyrazolones and their pyrazolone-urea derivatives were investigated in metastatic A431 and noncancerous HaCaT human keratinocytes by a mitochondrial activity test. The effects of the compounds on the migration of cancerous and noncancerous cell lines were investigated by using a cell scratch assay. The General Linear Model, Statistical Package for Social Sciences (SPSS v26) was used to determine if there was a statistically significant difference between the control and the treatment groups. Four of the nine compounds showed an antioxidant effect. All 5-pyrazolone-urea compounds showed higher toxicity (*p* < 0.05) in cancerous A431 cells compared to noncancerous cells at all time points. All compounds also showed a biphasic hormetic effect. Four of the nine compounds inhibited cell migration.

## 1. Introduction

The 5-pyrazolones are used for industrial purposes in the forms of metal extraction agents [1], anticorrosive components [2], and thermally stable polymers [3]. Besides, they are used in pharmaceutical chemistry for drug development [4]. Some of their pharmacological uses include analgesic, anti-inflammatory, antipyretic, antiparasitic, antimalarial, and antifungal effects [5]. Some 5-pyrazolones inhibit enzyme activity that causes neurodegenerative effects [6], and others are used as sedatives. In agricultural industries, they are used as pesticides against insects and weeds [7,8].

**Scheme 1:**
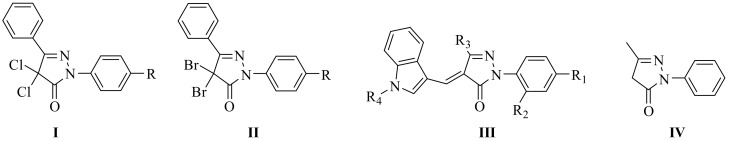
Some biologically active 5-pyrazolone derivatives.

In particular, when halogenated derivatives of pyrazolones are considered, 4,4-dichloro-5-pyrazolone derivatives (**I**) are human telomerase inhibitors, while 4,4-dibromo-5-pyrazolone derivatives (**II**) are important cytotoxic agents for the treatment of tumor metastasis. They also offer potential options for treating AIDS, diabetes, and autoimmune diseases [9]. While indole-derived 5-pyrazolones (**III**) have also been indicated to be a potential anticancer agent in the treatment of lung cancer [10], edaravone (**IV**) has been shown to exhibit a neuroprotective activity to prevent ischemic (brain, myocardial) paralysis due to its antioxidant properties (Scheme 1) [11].

The purpose of this research was to (1) synthesize new structures that have a pyrazolone building block (or core), which may also be essential starting materials that support the development of the pyrazolone library, and (2) investigate the biological potential of these newly synthesized structures, which have strong pharmacologic properties and reduced side effects.

Therefore, pyrazolones, which are obtained from the reactions of suitably substituted β-keto esters and substituted hydrazines, were formulated by the Vilsmeier–Haack reaction. Next, they were entered into a reaction with urea, which resulted in the synthesis of novel urea-pyrazolone derivatives. The biopotential of these compounds was investigated by using three approaches. First, the cytotoxic effects of these compounds on A431 malignant keratinocytes and noncancerous immortal HaCaT keratinocytes were evaluated by using a colorimetric 3-[4,5-dimethylthiazol-2-yl]-2,5-diphenyltetrazolium bromide (MTT) cell viability assay. Second, the antioxidant potential of these compounds on the prevention of cell death caused by hydrogen peroxide was investigated. Third, the effects of these compounds on cell migration were investigated by using a cell scratch assay on cancerous and noncancerous A431 and HaCaT cell lines.

## 2. Results and Discussion

### 2.1. Chemistry

**Scheme 2:**
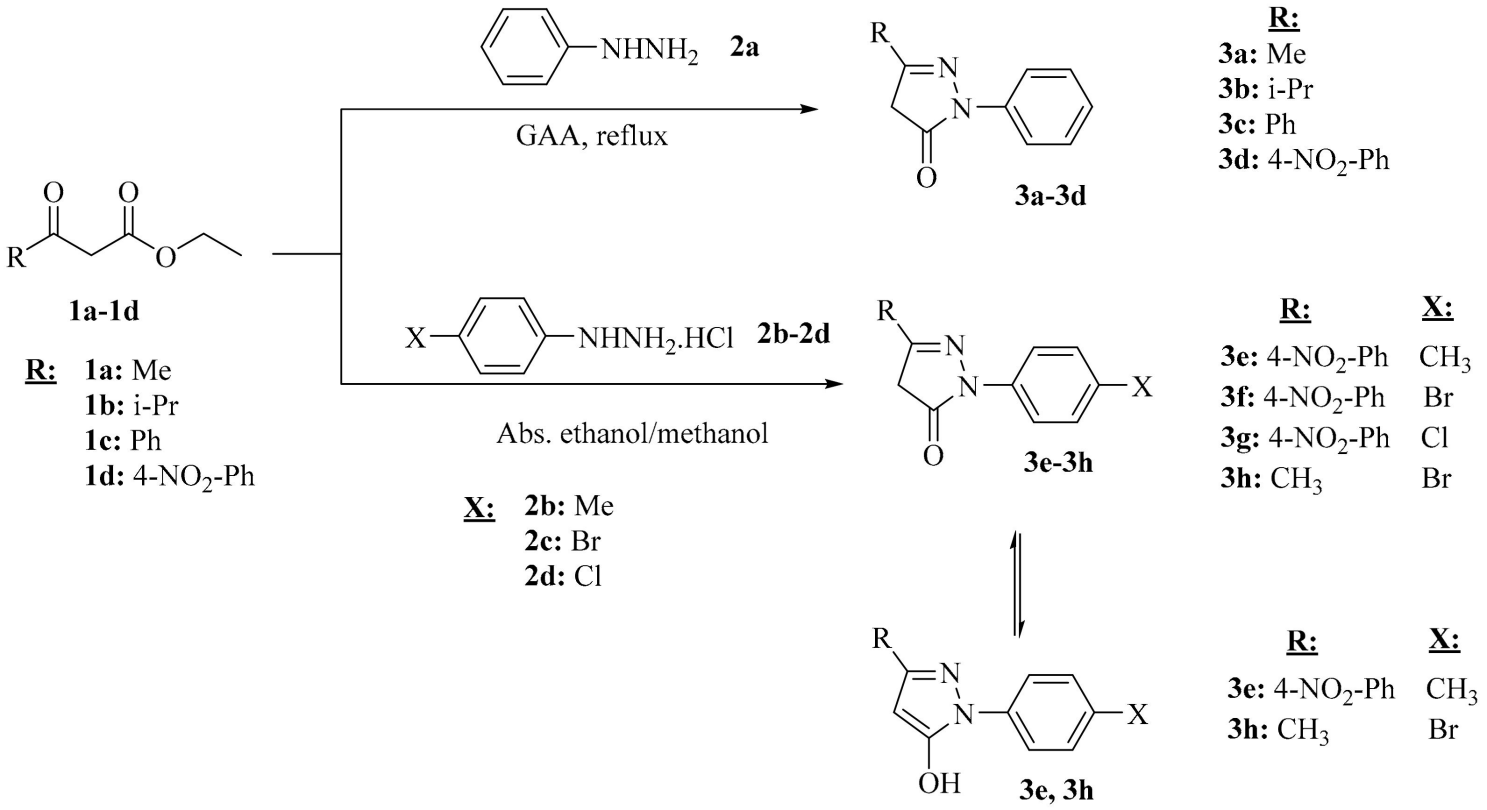
Synthesis of 5-pyrazolone compounds (**3a–h**).

The first four (**3a**–**d**) of the eight synthesized 5-pyrazolone compounds (**3a**–**h**) were obtained from the reflux of phenylhydrazine with glacial acetic acid in an oil bath with the appropriate β-keto ester [9]. Only minor changes were present in the work-up part of 3-(4-nitrophenyl)-1-phenyl-1*H*-pyrazole-5(4*H*)-one **3d** [12]. The remaining four 5-pyrazolone compounds (**3d**–**h**) were obtained from the reaction of the appropriate β-keto esters with phenylhydrazine hydrochloride in different reaction conditions (ethanol, at room temperature for **3h**, methanol reflux for (**3d–3g**)) (Scheme 2).

Furthermore, the compound **3h** has been previously described in the literature as an oily, yellow product of a reaction between p-bromophenyl hydrazine and 2,2-difluoro-4-alkoxy-1,3,2-dioxaborinane [13]. The spectral data of the compound obtained in solid state in this study, under much more mild conditions, were also consistent with the results of this study.

**Scheme 3:**
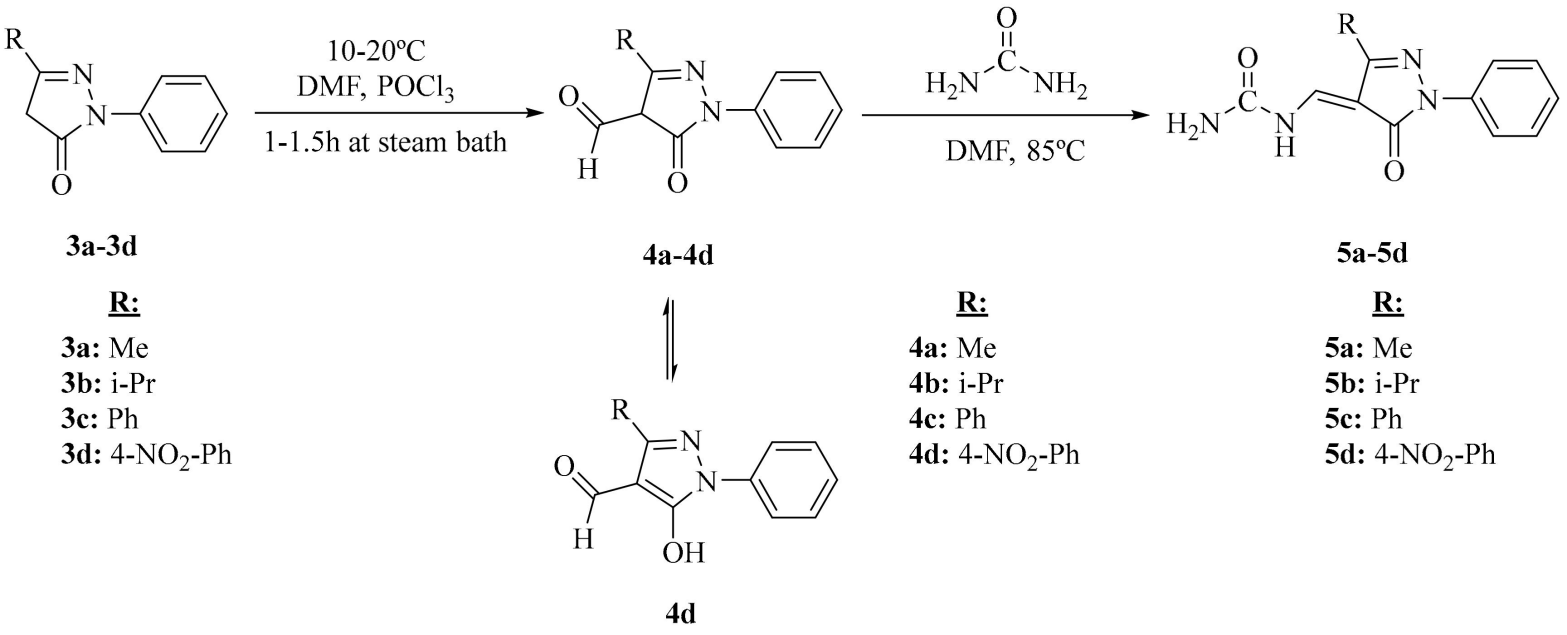
Synthesis of 4-formyl-5-pyrazolones (**4a–d**) and 5-pyrazolone-urea compounds (**5a–d**).

The 4-formyl-5-pyrazolone derivatives (**4a**–**d**) were synthesized by transferring the formyl group to the resulting 5-pyrazolone nuclei under Vilsmeier conditions and were provided with excess phosphorus oxychloride and *N*,*N*-dimethylformamide (Scheme 3). In the final step, these four aldehydes were reacted with excess urea in *N*,*N*-dimethylformamide (DMF) to give the desired ureamethylidene derivatives of pyrazolones (Scheme 3). Of these, the compound **4a** was previously synthesized by Porai and coworkers [14] by mixing 1-phenyl-3-methyl-4-hydroxymethylene-5-pyrazolone and urea in benzene. Crystallization and column chromatography methods, in different solvent systems, were applied to purify all compounds. All spectral data presented for characterization of these newly synthesized compounds (elemental analysis, FTIR, ^1^H and ^13^C NMR, and LC-MS/MS methods) were in agreement with each other.

### 2.2. FTIR

The IR spectra (Figures S1–S9 in Supplementary Materials) of the newly synthesized of pyrazolone compounds (**3e**–**h**) confirmed the chemical identity of the derivatives. Furthermore, the broad bands between 2681 and 2441 cm^1^ and the absence of any signal attributable to carbonyl stretching vibration proved that **3e** and **3h** mainly existed in enol form due to intramolecular chelation by hydrogen bonds in a solid state (Scheme 2).

In the pyrazolone-urea derivatives, the doublet peak between 3421–3294 and 3278–3124 cm^−1^ was determined as the stretching peaks of –NH_2_ group, between 3201–3190 cm^−1^ to single peak as the stretching of urea -NH group, between 1759–1662 cm^−1^ as the stretch peak of pyrazolone carbonyl, and between 1673–1643 cm^−1^ as the stretch peak of urea carbonyl.

### 2.3. NMR

**Scheme 4:**
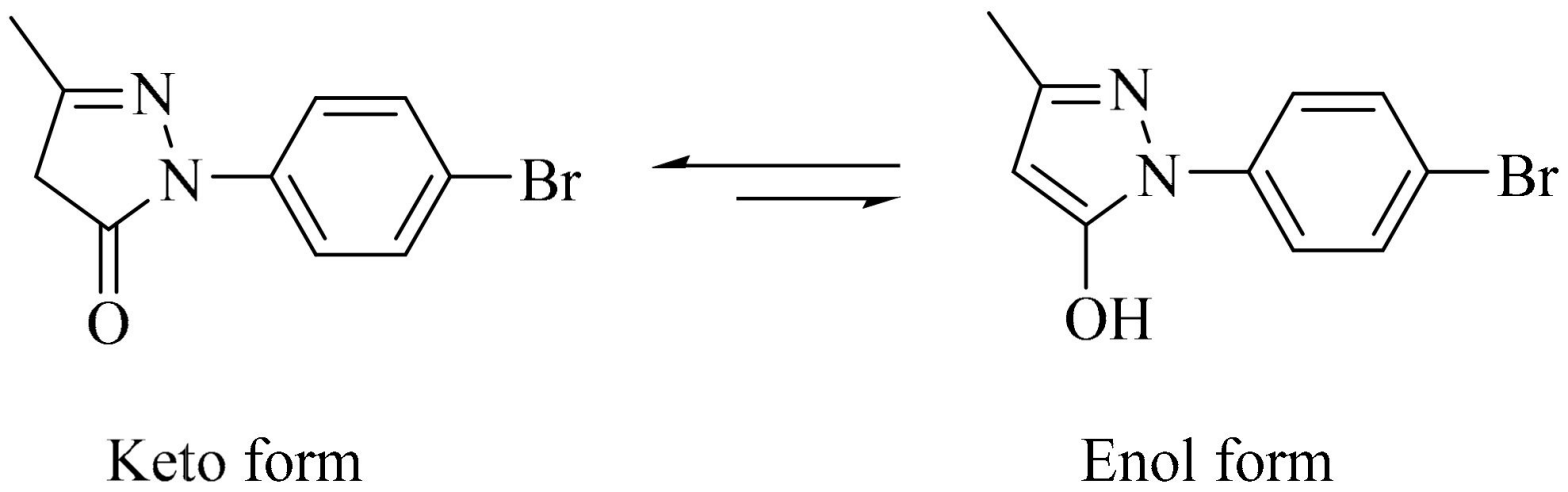
Tautomeric form of the compound **3h** in CDCl_3_.

In the ^1^H-NMR spectra taken in CDCl_3_ of (**3f**–**h**) compounds, -CH_2_ signals of the pyrazolone ring respectively gave resonance at 3.92 ppm (for **3f** and **3g**) and 3.42 ppm (for **3h**) as singlets (Figures S11–S13 in Supplementary Materials). In the literature on tautomeric forms of 5-pyrazolones, it is stated that they can exist in three main tautomeric structures. The research indicates that the form which will be more dominant depends mainly on the solvent. In the gaseous phase, apolar solvent and solvents with low polarity, such as CHCl_3_, CH_2_Cl_2_, and CCl_4_, only the CH form is observed. In polar aprotic solvents, such as DMSO, THF, and CH_3_CN, the mixture of OH and NH structures having a dynamic equilibrium with each other is predominant as the main form [15,16]. The formation of these forms is dependent on specific solvation effects and electrostatic interactions between solvent and solute. If there is a structure supporting the hydrogen bond acceptor near the hydroxyl group, the OH form is observed, and the structure is stabilized by the in-molecule O–H…O bonds [17]. The compound **3h** synthesized has a clear -CH_2_ signal in the pyrazolone ring, especially in proton NMR, and the absence of a broad peak of 10–12 ppm OH proton in ^1^H-NMR confirms that the balance shifts to CH form in CDCl_3_ (Scheme 4).

The ^1^H-NMR spectrum obtained in DMSO-*d_6_* of compound **3e** contained an OH tautomer. This was confirmed by the presence of pyrazolone proton at 6.20 ppm and hydroxy proton δ at 12.01 ppm (Figure S10). In the ^1^H-NMR spectrum of compound **4d**, the peak at δ 10.10 ppm confirmed that the formyl group was attached to the C4 carbon of the starting compound, the pyrazolone ring. This peak confirmed that the formyl group was attached to the C4 carbon of the pyrazolone ring of the starting compound (Figure S14). Besides, the presence of the OH peak of the hydroxyl proton at 9.90 ppm showed that this compound had the OH tautomeric structure.

**Scheme 5:**
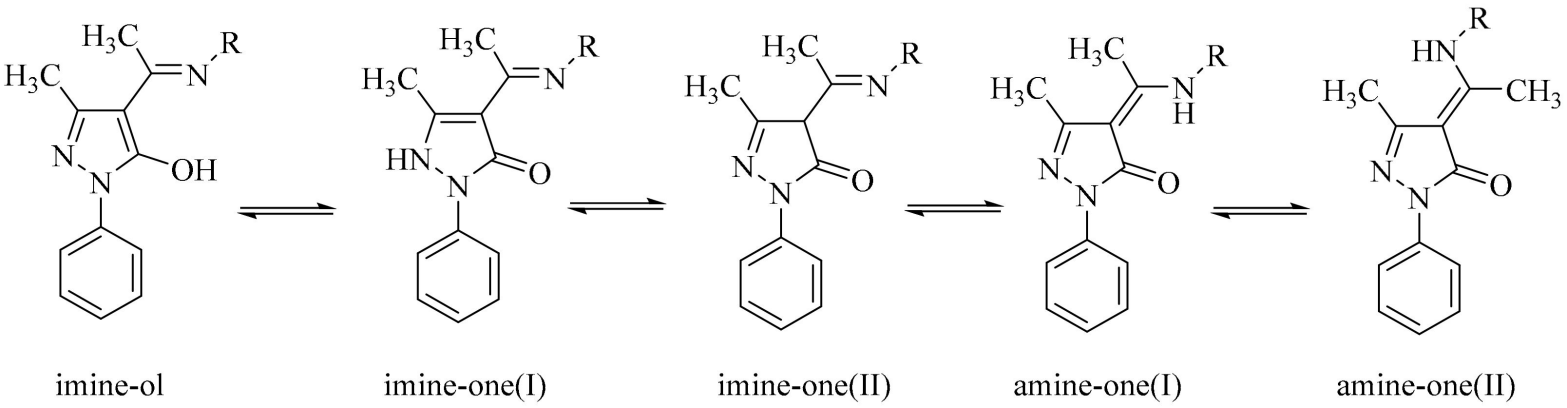
Tautomeric forms of Schiff-based derivatives of 4-acylpyrazolones [18].

When the ^1^H-NMR of the pyrazolone-urea derivatives (**5a**–**d**) were examined, a proton-NH signaling doublet at 11.1–9.69 ppm in the downfield of the spectrum, a proton-CH signaling doublet at 8.31–8.18 ppm, at 9.47–7.79 ppm -NH_2_ signals, which resonated in the form of small broad peaks, were observed. Amarasekara and coworkers [18] stated that Schiff-based derivatives prepared from 4-acetyl-5-methyl-2-phenyl-2,4-dihydro-pyrazole-3-one compounds with alkyl amines are present in the tautomeric form of amine-one (**I**) in chloroform in NMR. Pyrazolones and 4-acylpyrazolones are known to exhibit interesting keto-enol tautomerism, and in principle, Schiff-based derivatives of 4-acylpyrazolones may exist in five possible tautomeric forms, imine-ol, imine-one (**I**), imine-one (**II**), amine-one (**I**), and amine-on (**II**) (Scheme 5).

**Scheme 6:**
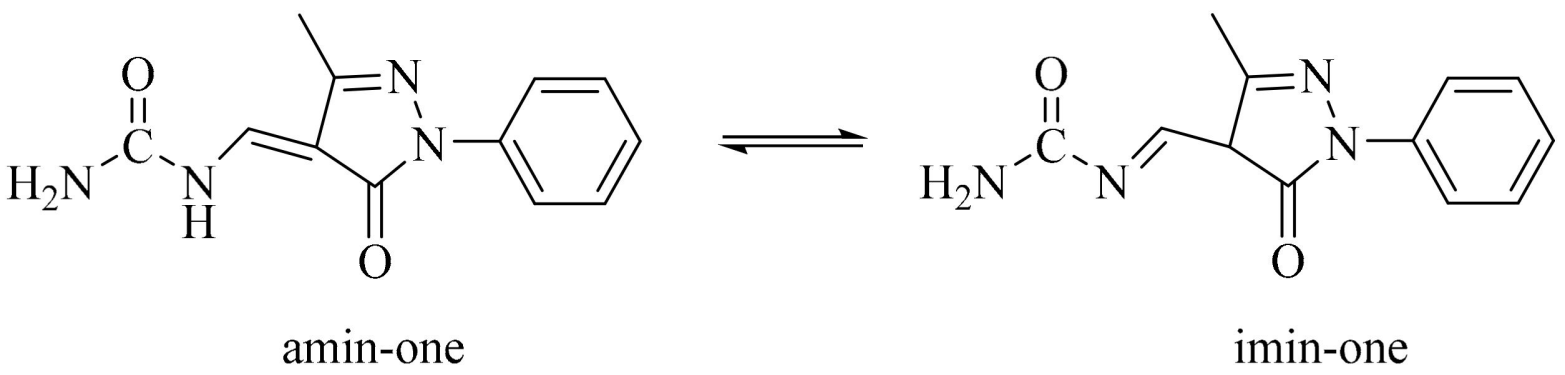
Tautomerism of synthesized compounds (**5a–d**).

The synthesized pyrazolone-urea compounds (**5a**–**5d**) had the –NH. The amino (-NH_2_) and carbonyl (C=O) vibration mode bands were seen in the IR spectrum, and the -NH, -NH_2_, and -CH peaks seen in the ^1^H-NMR showed not to prefer the imine-one form of the pyrazolone ring. This supports the presence of the amine-one tautomeric form, where the urea molecule was attached to the carbon C4 as C = C bond (Scheme 6).

In the ^13^C spectra for (**3f**–**h**) of the pyrazolone compounds (Figures S19–S27 in Supplementary Materials), the -CH_2_ (C4 carbon) carbon of the pyrazolone ring resonated at 43.1 ppm (for **3f**) and 39.32 ppm (for **3g** and **3h**). The pyrazolone carbonyl (C=O pyrazolone) resonated at 170.5 ppm for compound **3f**, and at 169.7 ppm for compounds **3g** and **3h**. These signals showed us that all three pyrazolones were compatible with the form -CH_2_. Our results were found to be compatible with the spectral values of **3h** yellow oily compound obtained by reaction of 2,2-difluoro-4-alkoxy-1,3,2-dioxaborinane with p-bromophenyl hydrazine, as reported by Stefane and coworkers [13]. Ragab and coworkers [8], in their studies involving the synthesis of 4-substituted-1*H*-pyrazole-5 (4*H*)-one, 1-(4-chlorophenyl)-3-phenyl-1*H*-pyrazole-5(4*H*)-one in ^13^C-NMR spectrum, showed the C5 carbon, to which the enolic OH group is attached, at 154 ppm. Similarly, the compound **3e** synthesized in the pyrazolone ring that was linked to the hydroxy group C5 carbon resonated at 154.5 ppm, while the C4 carbon of the pyrazolone ring resonated at 86.5 ppm, and the C3 carbon resonated at 147.7 ppm.

The ^13^C spectrum of compound **4d**, which was a formulated pyrazolone, pointed to the aldehyde group attached from the fourth carbon to the peak pyrazolone ring, which was resonant at 183 ppm. In the pyrazolone ring, the C5 carbon, to which the hydroxy group was attached, resonated at 150.9 ppm, while the C4 carbon of the pyrazolone ring resonated at 116.8 ppm.

For the urea compounds (**5a**–**d**), in the ^13^C spectra, signals that belonged to the C4 carbon, to which the urea molecule was attached, were seen at 104.8–91.73 ppm, while the pyrazolone carbonyls were seen at 165.8–164.5 ppm (C=O pyrazolone), and the resonances of urea carbonyls in the compounds were observed at 157.8–152.9 ppm. The NH-CH carbons in the compounds also peaked at 139.2–138.8 ppm. Besides this, our results were found to be consistent with the spectral values of compound **5a** obtained by the reaction of urea with edaravone in benzene, as reported by Porai-Koshits and coworkers [14].

### 2.4. Biological Activity of Compounds

The role of reactive oxygen species (ROS) in cancer initiation, promotion, and progression is well documented [19]. ROS induce mutations by causing the oxidation of DNA via Fenton reactions [20,21]. Antioxidants stabilize the free radicals, thus preventing the mutations that result from the damage, which may lead to cancer [22]. Previous studies have also reported that some pyrazole derivatives might prevent metastasis because of their potential as anti-angiogenesis agents [23]. Because of these reports, the cytotoxic, antioxidant, and antimetastatic potential of the compounds were tested. The antimetastatic potential of the compounds was tested by using cell scratch assay. In order to assure that the wound closure was due to cell migration and not cell proliferation, we serum-starved the cells for 24 h before wounds were created.

Furthermore, 2 µg/mL colcemid was added to the medium to inhibit cell proliferation. To minimize the cytotoxic effects of compounds on cell migration, cells were treated with 1 mM compound. None of the tested compounds showed a cytotoxic effect on the viability of A431 and HaCaT cells at this concentration.

#### 2.4.1. Effects of Compounds on Cell Viability

To examine the impact of compounds on the viability of cancerous vs. noncancerous cells, we exposed the A431 metastatic skin cancer cells and HaCaT immortalized noncancerous skin cells to seven concentrations (0.001, 0.005, 0.01, 0.05, 0.1, 0.5, and 1 mM) of each of the compounds for 24, 48, 72, and 96 h (Figures S37–S46 in Supplementary Materials). Control group received only vehicle (0.1% DMSO). The cytotoxic effect of compounds was measured by using methyl thiazole tetrazolium (MTT) assay, which measures cell viability and proliferation based on the reduction of MTT to formazan. Statistical analyses of the effects of the compounds on cell viability were determined using a The General Linear Model, Statistical Package for Social Sciences (SPSS v26).

All compounds containing urea (**5a**–**d**) showed higher toxicity (*p* < 0.05) to cancerous A431 cells when compared to the noncancerous cells at all time points (except for **5b** at 48 h and **5d** at 96 h). Compounds (**5a**–**d**) also showed a biphasic response (hormesis). Exposure to low concentrations caused an increase in cell viability, whereas high concentrations reduced cell viability (*p* < 0.05). The degree of biphasic dose response varied for different compounds (Figures S42–S45).

**Figure 1:**
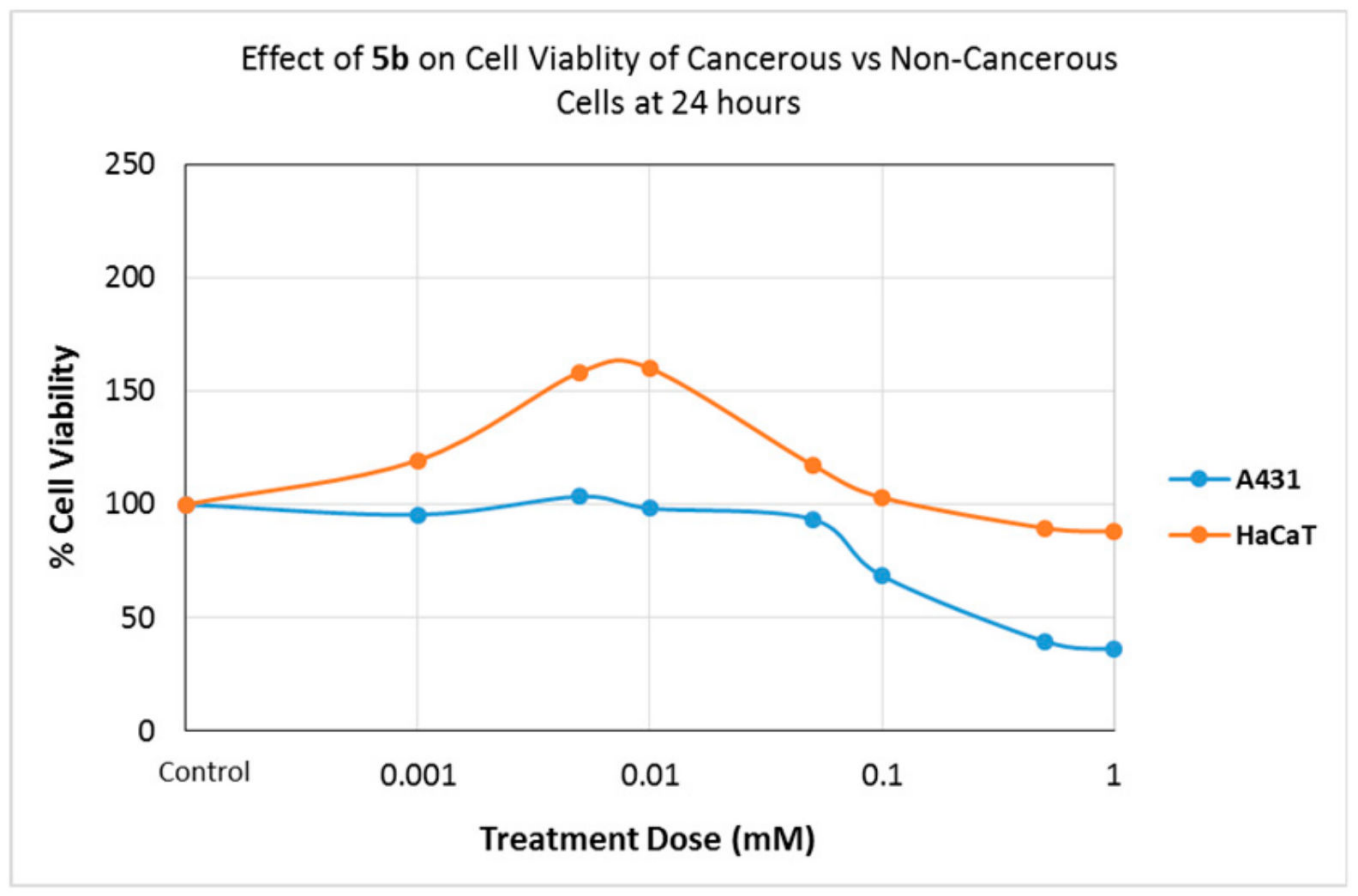
Effect of **5b** on cell viability of cancerous vs. noncancerous cells at 24 h.

Compound 5a reduced cell viability of cancerous A431 cells by 80% at higher concentrations (0.5 mM and higher). This proved that Compound **5a** was selectively toxic to the cancerous cells as it showed minimal toxicity (10%) to noncancerous HaCaT cells under the same conditions (Figure S42). The cytotoxic effect of **5b** was higher on cancerous A431 cells. As the treatment time and dose increased, the cytotoxic effect increased in both cell lines (*p* < 0.05). After 24 h of treatment, compound **5b** showed a hormetic effect on HaCaT cells, whereas it did not cause any increase in viability of cancerous A431 cells at high concentrations (Figure 1).

**Figure 2:**
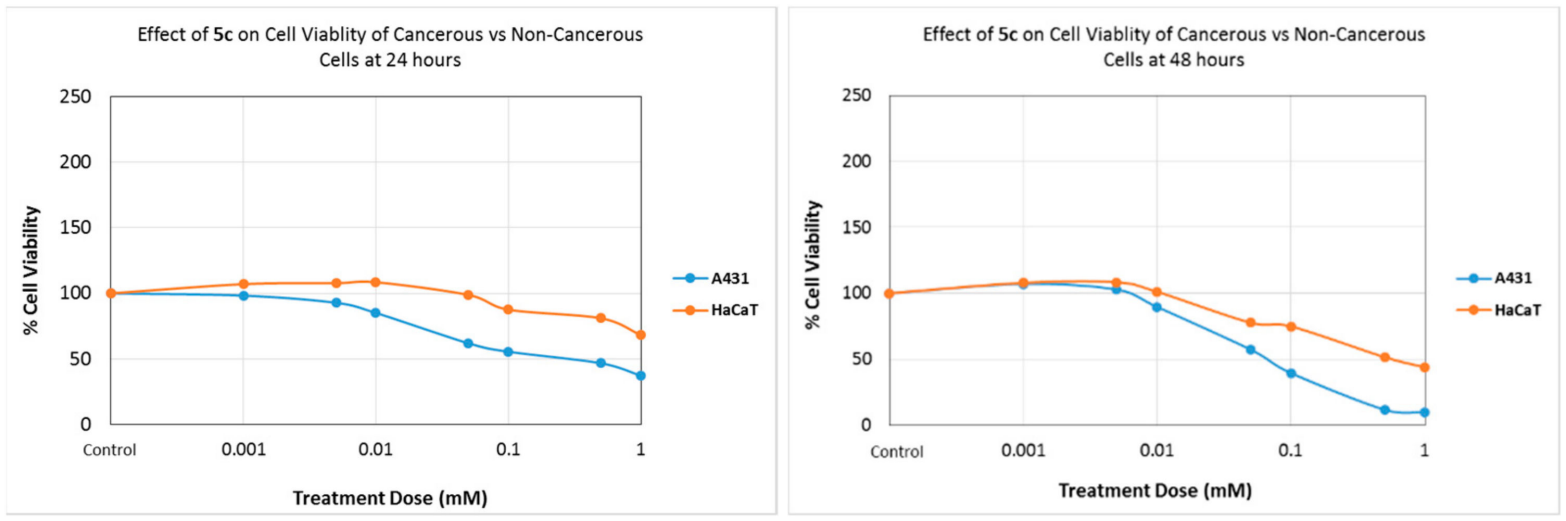
Effect of **5c** on cell viability of cancerous vs. noncancerous cells at 24 h (**left**), 48 h (**right**).

Compound **5c** also showed a higher toxicity level to cancerous cells (*p* < 0.05), particularly when treated with high concentrations. Toxicity of **5c** was also time-dependent. Longer treatment times increase toxicity showing that the compound was stable in cell culture media for 76 h (Figure 2).

Compound **5d** (Figure S45) lost its toxic effect after 24 h of treatment, which demonstrated that the compound might be unstable in solution.

Compounds **3e** and **3f** showed hormesis at low concentrations. A dose- (*p* < 0.05) and time- dependent (*p* < 0.05) increase in the cytotoxic effect was observed for cancerous A431. Both compounds showed lower toxicity to HaCaT cells (Figure S37 and S38).

All of the seven compounds discussed above had differential toxicity to cancerous cells (*p* < 0.05) and low toxicity to noncancerous cells. Drugs that were selectively toxic to cancerous cells were highly sought after since nonselective drugs cause a deleterious effect on healthy human cells. Therefore, we plan to conduct further investigations to discover the biochemistry of this selective toxicity. This is not only significant for uncovering the pharmaceutical potential of these compounds, but also an understanding of the bases for this selective toxicity can provide invaluable insights to researchers. It will help researchers to develop drugs that are highly toxic to tumor cells while they are minimally toxic to the healthy cells.

**Figure 3:**
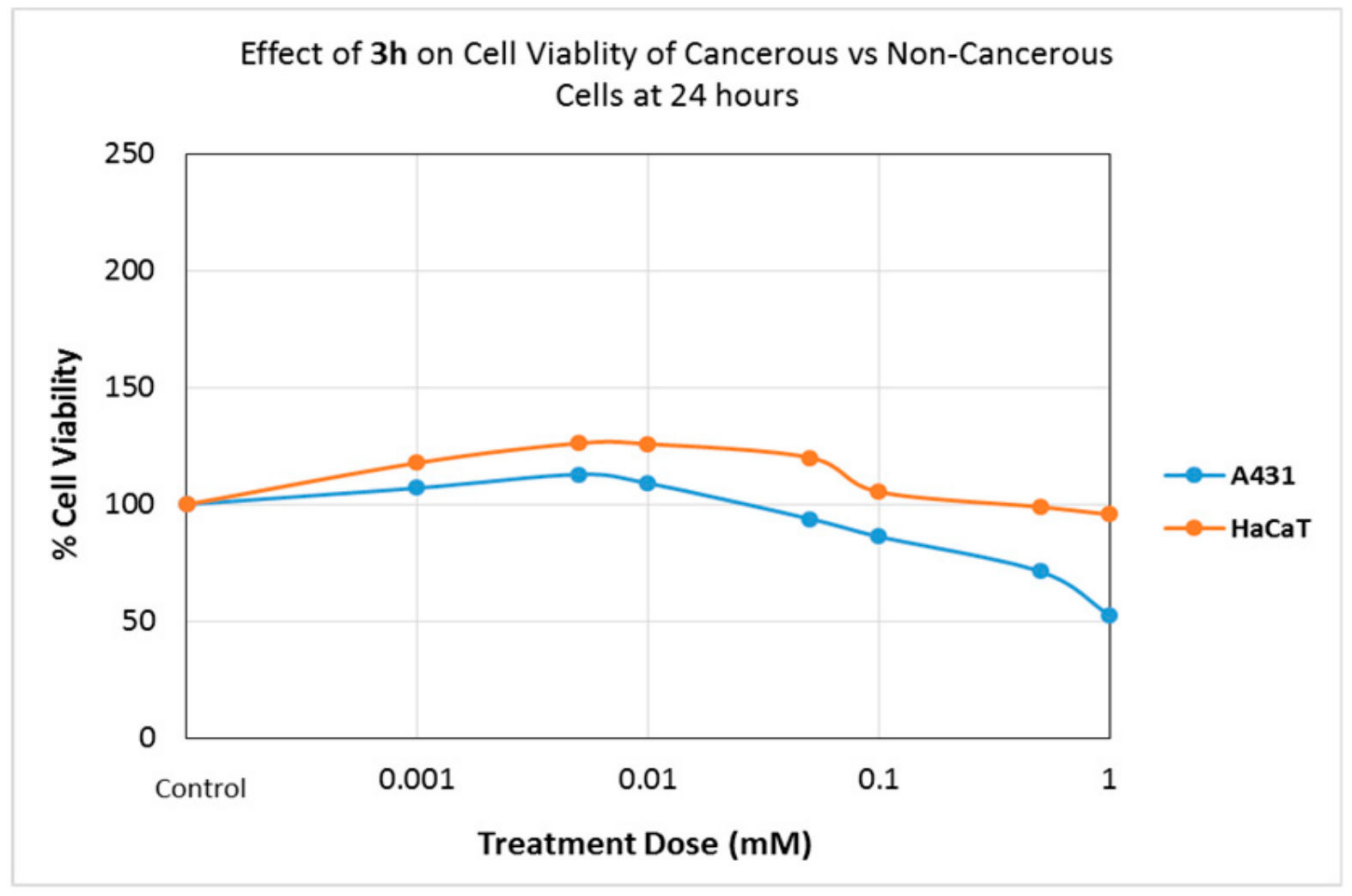
Effect of **3h** on cell viability of cancerous vs. noncancerous cells at 24 h.

Compounds **3h** and **4d** caused time- and dose-dependent cytotoxic effects (*p* < 0.05), both in cancerous and noncancerous cells (Figure 3). Even though they had differential cytotoxicity (*p* < 0.05), their cytotoxic effect was higher to noncancerous cells when compared to the cancerous cells. Compound **3g** caused a nearly 100% toxicity to both cancerous and noncancerous cell lines at 96 h and high concentrations (p < 0.05). Because compounds **4d** and **3g** showed higher toxicities to HaCaT cells, we speculate that their potential applicability for pharmaceutical uses is limited.

Recent research shows that pyrazole derivatives show cytotoxic effects on a variety of cell lines via several different biochemical mechanisms. For example, ferrocenyl pyrazole derivatives were reported to inhibit phosphoinositide 3-kinase/Protein kinase B (PI3K/Akt) and extracellular signal-regulated kinases (ERK 1/2) signaling pathways [24]. PI3K/Akt and ERK 1/2 signaling pathways are the two primary pathways involved in regulating cell proliferation, cell cycle, and apoptosis. Activation and function of both pathways are under the tight control of multistep signaling control mechanisms. A significant number of tumor cells (e.g., 41% of all melanomas) show some form of mutation in either PI3K/Akt or ERK signaling. Currently, the PI3K/Akt and ERK signaling pathways are among the most significant molecular targets for cancer therapy. Drugs that inhibit one of the activation steps of these pathways prevent cancer cell proliferation and trigger cell death. Ferrocenyl pyrazoles are reported to inhibit PI3K/Akt and ERK 1/2 signaling pathways in cancerous Michigan Cancer Foundation-7 (MCF-7). Ferrocenyl pyrazoles are also reported to cause membrane blebbing in MCF-7 breast cancer cells. The same researchers reported that nontumorigenic MCF-10F cells do not show membrane blebbing or inhibition of PI3K/Akt and ERK 1/2 signaling pathways when treated with ferrocenyl pyrazoles under the same conditions [24]. We recommend investigating the effects of the compounds used in this project on PI3K/Akt and ERK 1/2 signaling pathways on two different cell lines.

Similarly, Kim and his colleagues [25] reported that some phenylpyrazolodiazepin-7-one derivatives prevent cell proliferation in tumorigenic cells via selective Raf kinases inhibitor activity. Raf is the key activator of the ERK pathway, which is deregulated in one-third of all human cancers. Raf kinase activates the Raf protein causing the activation of the ERK pathway. Therefore, Raf kinase inhibitors are molecular targets for cancer drug development. Kim et al. also reported [25] that 1*H*-pyrazole-3-yl-amide derivatives reduce cell viability in several human cell lines by inhibition of cyclin-dependent kinases [26]. Cyclin-dependent kinase controls cell cycle by phosphorylating proteins directly involved in cell division. Mutations in cyclin-dependent kinases lead to cancer by promoting uncontrolled cell proliferation. Drugs that inhibit cyclin-dependent kinase activity prevent cancer cells from dividing. We recommend investigating the effects of the compounds used in this project on Raf kinase and on cyclin-dependent kinases.

#### 2.4.2. Cell Migration Assay

**Figure 4:**
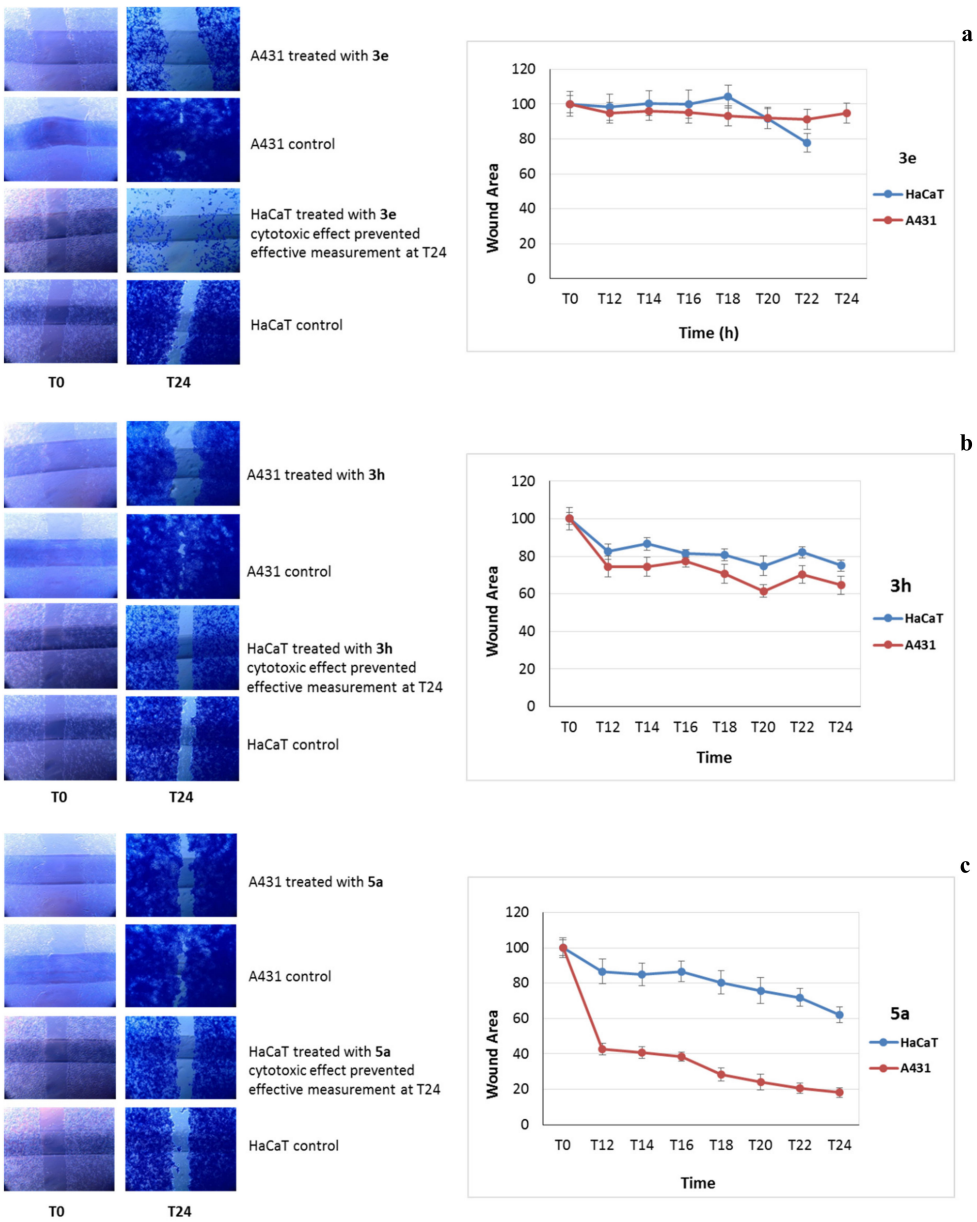

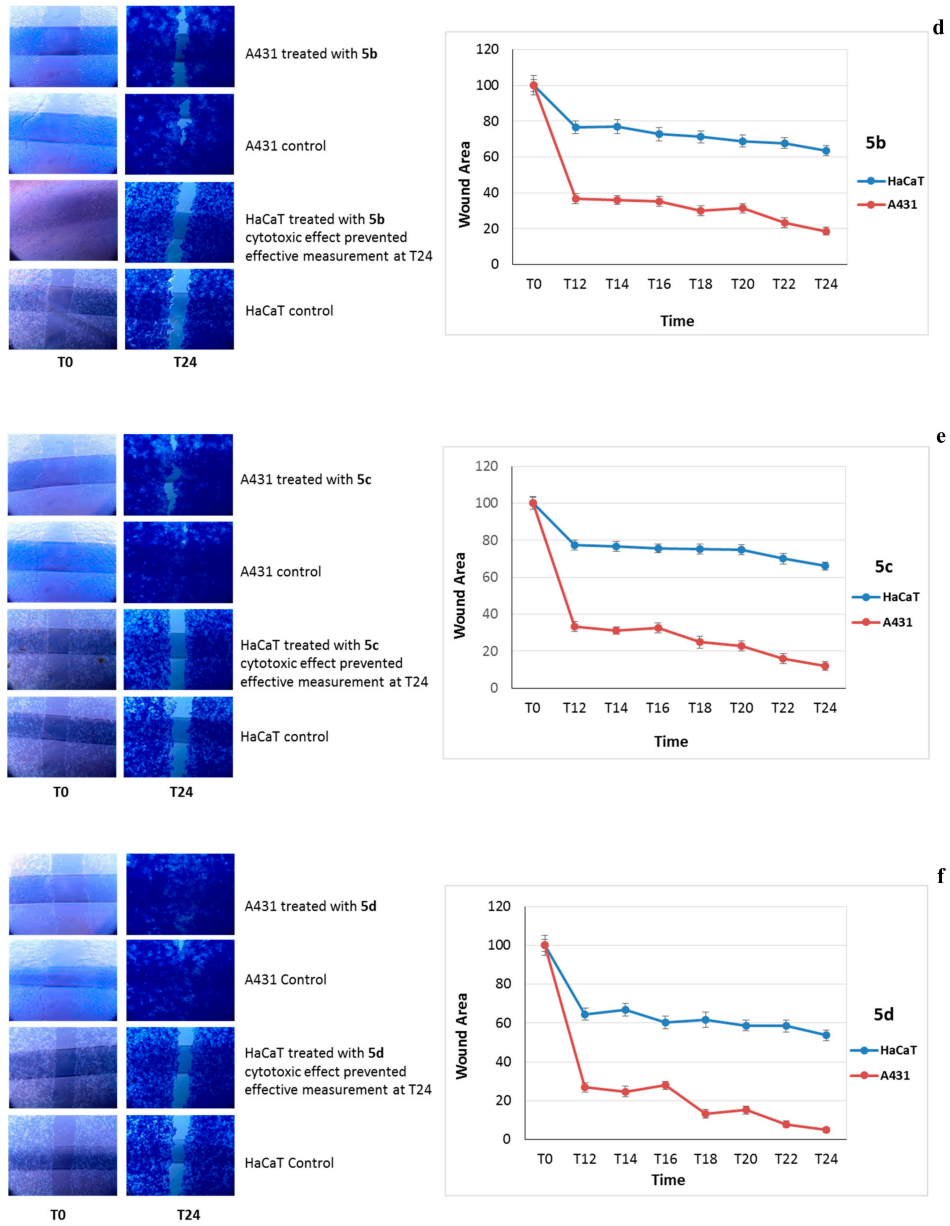
The effect of compounds (**a**) **3e**, (**b**) **3h**, (**c**) **5a,** (**d**) **5b**, (**e**) **5c**, and (f) **5d** on cell migration of cancerous A431 vs. noncancerous HaCaT keratinocytes.

A wound-healing assay was performed to investigate whether the compounds inhibit the migration and invasion capabilities of human keratinocytes. Cell migration was evaluated by measuring the invasion of an artificial wound by the cells (Figure 4). To distinguish wound healing via cell proliferation from wound healing via cell migration, cells were kept in a serum-free medium containing 2 µg/mL colcemid starting 24 h prior to treatment. Cells were treated with 1 μM (micromolar) compound. This concentration was chosen because none of the compounds showed a cytotoxic effect on A431 and HaCaT cell at this concentration. The control group received only vehicle (0.1% DMSO). An image at time zero was taken as a baseline, immediately after the artificial scratch wounds were created. The wound closure was monitored over a 24-h period. Time-lapsed images of scratch wounds were taken every two hours, starting at a time point of 12 h after the wounds were created. The percent of cell migration was calculated and compared in cancerous vs. noncancerous keratinocytes and also between the control and treatment groups.

Compound **3e** prevented cell migration of cancerous and noncancerous keratinocytes (*p* < 0.05). In the noncancerous HaCaT cell line, there was a 30% decrease in the control wound area 12 h after the treatment. This stayed relatively stable over the 24-h periods. The treatment group showed no wound closure for 22 h following the treatment. However, when the cells were stained, they were washed away from the wells; therefore, no measurements were made at 24 hours following the treatment (T24). Because compound **3e** prevents the migration of cancerous cells, it should be further investigated for its ability to reduce cancer metastasis (Figure 4a). Compound **3f** inhibited the migration of cancerous A431 keratinocytes (*p* < 0.05) for the first 18 h, whereas it caused an increase in the migration of noncancerous HaCaT cell line (*p* > 0.05) after 12 h of treatment. We were not able to photograph this group after 24 h of treatment. Cells detached entirely from the wells when they were treated with cold methanol for staining.

Compound **3h** caused a decrease in relative cell invasion, both in cancerous and noncancerous keratinocytes (*p* < 0.05). The change in the pattern was similar, both in cancerous and noncancerous cell lines; however, the difference between the control and treatment groups was higher in cancerous A431 cells. Compound **3f** prevented the migration of A431 cells by 60% when compared to control cells (*p* < 0.05). Because compound **3f** reduced the migration of cancerous cells without causing a significant change to noncancerous cell behavior, it should be further investigated for its ability to reduce cancer metastasis (Figure 4b).

Compound **5a** caused a decrease in relative cell invasion both in cancerous and noncancerous keratinocytes (*p* < 0.05). An 8% reduction in cell migration in the noncancerous cell line (*p* < 0.05) and a 13% reduction in the migration of cancerous cells (*p* < 0.05) were observed. Both the reduction in cell migration and the difference between the migration of cancerous and noncancerous cell lines were found to be statistically significant (*p* < 0.05). Similar to compound **5a,** compound **5b** caused a decrease in relative cell invasion in cancerous keratinocytes (*p* < 0.05). However, it did not cause any change to the cell migration pattern of control or treatment groups in HaCaT cell lines. Twenty hours after wounds were created, about 32% of wound closure was observed in the control group, and 33% closure was observed in the treatment group of the HaCaT cells. However, in cancerous A431 cells, 20 h after wounds were created, about 88% of wound closure was observed in the control cells, whereas about 69% of wound closure was observed in the treatment group (*p* < 0.05). This 19% reduction in cell migration by the compound **5b** was statistically significant (p < 0.05). Because both compounds **5a** and **5b** reduced the migration of cancerous cells, especially **5a,** without causing a change to noncancerous cell behavior, they should be further investigated for their ability to reduce cancer metastasis (Figure 4c,d).

The control group and treatment groups for compounds **5c** and **5d** caused a time-dependent increase in the amounts of cells that migrated to the center of the wound for the control and treatment groups of both cancerous and noncancerous cell lines (Figure 4e,f). However, the changes were not statistically significant (*p* > 0.05). Previous research has reported that diaryl urea compounds inhibit p38 MAP kinase, which is a protein that is activated in response to cellular stress. When activated, p38 MAP kinase causes the activation of several transcription factors. Changes to the activity of p38 are shown to lead to an excessively high activity of transcription factor nuclear factor kappa-light-chain-enhancer of activated B cells (NF-κB), which coordinates cell survival and death [27]. Abnormalities in NF-κB activity are reported to cause cancer, as well as activation of genes that promote cancer cell metastasis [28]. Therefore, chemicals that can inhibit p38 protein are possible therapeutic agents. We recommend testing the effects of the compounds studied in this research on the viability of cells, and cell migration may be a result of the interaction with p38 protein.

#### 2.4.3. Preventing Cell Death Caused by Hydrogen Peroxide

**Figure 5:**
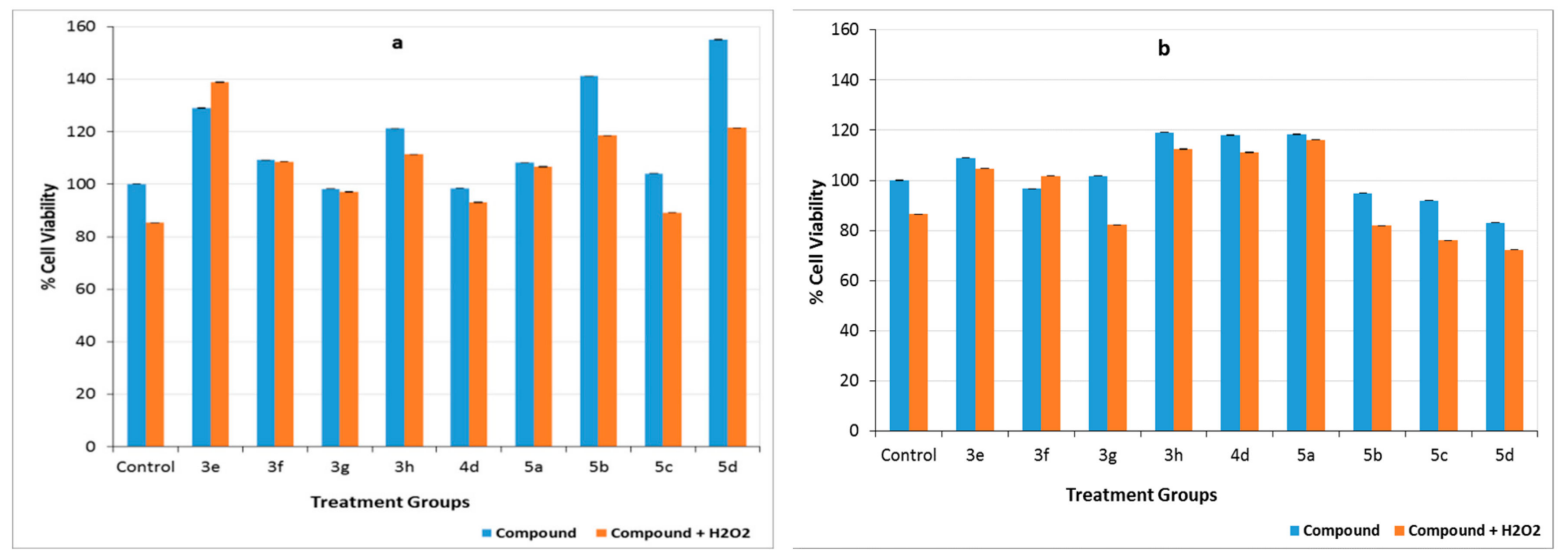
H_2_O_2_-induced cell death protective effects of compounds (**a**) in cancerous A431 and (**b**) in noncancerous HaCaT cell lines.

Our data showed that all of the nine compounds studied in this project had a hormetic effect on either one or both cell lines. Hormesis is the biphasic dose response against various agents. The typical hormetic effect is characterized by stimulation or the increase in cell viability when exposed to a low dose followed by an inhibitory or cytotoxic effect on cell viability when exposed to a higher dose [29]. Even though the mechanisms behind hormesis are not well understood, several researchers have reported that the antioxidant activity of various compounds could cause hormetic effects [30,31,32]. Because pyrazoles are known to have a high antioxidant activity, the antioxidant capacity of the compounds was examined by investigating their ability to prevent cell death caused by H_2_O_2_. Because HaCaT cells and A431 cells are reportedly strongly resistant to the effects of H_2_O_2_ [33], a relatively high dose of H_2_O_2_ was used in these experiments. Our data showed that compounds **3e**, **3f**, **3g**, and **5a** prevented H_2_O_2_-induced cell death in noncancerous HaCaT cell lines (*p* < 0.05), whereas only **3f** prevented the H_2_O_2_-induced cell death in A431 cell lines (*p* < 0.05) (Figure 5). We did not investigate the mechanism behind the protection from H_2_O_2._ Because exogenously applied H_2_O_2_ induces cell death in human cells via intracellular superoxide (O_2_*-*) generation [34], we speculate that these compounds could prevent H_2_O_2_-induced cell death via their antioxidant capacity.

## 3. Materials and Methods

### 3.1. Materials and Physical Measurements

All of the reactants were used as purchased from Alfa Aesar (Heysham, Lancashire, UK), Merck (Darmstadt, Germany), Sigma-Aldrich (St. Louis, MO, USA), and Fluka (Mexico City, MO, USA) and all solvents were used without further purifications. The water content of DMF was less than 0.1%. All of the obtained products were purified by column chromatography, using silica gel 60 (0.063–1.100 mm). The melting points were determined in open glass capillaries in the Electrothermal IA9200 melting point apparatus (Staffordshire, UK) and were uncorrected. A Shimadzu Affınity-1 FTIR spectrophotometer (Duisburg, F.R. Germany) was used to record IR spectra using the attenuated total reflectance (ATR) method. The ^1^H-NMR spectra were recorded in a Varian Mercury-400 (Seattle, WA, USA), 400 MHz, while ^13^C NMR spectra were recorded in a Varian Mercury-400, 100 MHz in DMSO-*d_6_*, and CDCl_3_ as a solvent. Mass spectra were obtained on a 3200 QTrap-AB Sciex LC-MS/MS spectrometer (Darmstadt, Germany). Carbon, hydrogen, and nitrogen were estimated in a PerkinElmer 2400 Series II CHNS/O Elemental Analyzer (Seattle, WA, USA). The results of the elemental analyses (Table S1) and the LC-MS/MS spectra (Figures S28–S36) were all consistent with their respective molecular formulas. All of the reactions were monitored by thin-layer chromatography (TLC) on precoated silica gel 60 F_254_ aluminum sheets (Merck); spots were visualized under UV light at 254 nm.

### 3.2. Synthesis of Compounds

#### 3.2.1. Synthesis of 5-Pyrazolone Compounds (**3a**–**c**) Obtained from Phenylhydrazine

The 17 mmol of the appropriate β-ketoester was reacted with 14 mmol of phenylhydrazine and 5 mL of glacial acetic acid. It was refluxed in an oil bath. After the reaction was complete, the mixture was evaporated. The remaining oily residue was solidified in an ice bath by the addition of cold diethyl ether or hexane (20 mL). The solids were filtered through Gooch crucible and were passed 2–3 times through cold ether. Then column chromatography was performed.

*The 3-methyl-1-phenyl-1H-pyrazole-5(4H)-one* (**3a**). This compound was synthesized according to the general procedure using 17 mmol (2.21 g) of ethyl acetoacetate and 14 mmol (1.51 g) of phenylhydrazine in 5 mL of glacial acetic acid by refluxing for 6 h. SiO_2_; n-hexane/ethyl acetate 2:1- Retention factor (*R_f_*): 0.16. The resulting solids were recrystallized in the benzene-petroleum ether (1:3) and collected by filtration to give 0.999 g (41%) as a white solid, Melting point (mp): 127–130 °C ([9], 127–129 °C). Compounds (**3a**–**c**) were synthesized according to the literature [9], and their FTIR, and ^1^H and ^13^C NMR spectra corresponded to the previously reported data.

*The 3-isopropyl-1-phenyl-1H-pyrazole-5(4H)-one* (**3b**). This compound was synthesized according to the general procedure using 17 mmol (2.50 g) of methyl isobutyrylacetate and 14 mmol (1.51 g) of phenylhydrazine in 5 mL of glacial acetic acid by refluxing for 12 h. SiO_2;_ n-hexane/ethyl acetate 2:1- *R_f_*: 0.29. The solid was recrystallized from benzene-petroleum ether (1:3). White solid, yield: 0.991 g (35%), mp: 100–101 °C ([9]; 101–103 °C).

*The 1,3-diphenyl-1H-pyrazole-5(4H)-one* (**3c**). This compound was synthesized according to the general procedure using 17 mmol (3.41 g) of ethyl benzoylacetate and 14 mmol (1.51 g) of phenylhydrazine in 5 mL of glacial acetic acid by refluxing for 8 h. SiO_2;_ n-hexane/ethyl acetate 2:1- *R_f_*: 0.32. The solid was recrystallized from benzene. White solid, yield: 0.153 g (61%), mp: 137–139 °C ([9]; 136–138 °C).

*The 3-(4-nitrophenyl)-1-phenyl-1H-pyrazole-5(4H)-one* (**3d**). 10 mmol (2.3 g) ethyl 4-nitrobenzoyl acetate, 10 mmol (1.4 mL) phenylhydrazine, and 10 mL glacial acetic acid were refluxed in an oil bath for 5 h. After the reaction was complete, the mixture was evaporated. The residue was dissolved in ethyl acetate (20 mL) and extracted with saturated NaHCO_3_ (2 × 10 mL). The organic phase was re-extracted with saturated brine, then dried with Na_2_SO_4_, and the solvent was then evaporated. Then column chromatography was performed (SiO_2_; n-hexane/ethyl acetate 2:1- *R_f_*: 0.22). The solid was recrystallized from ethanol, and collected by filtration to give 1.096 g (39%) as a yellow solid, mp: 188–191 °C ([12]; 190–191 °C). Compound **3d** was synthesized according to the literature [12], and its FTIR, and ^1^H and ^13^C NMR spectra corresponded to the previously reported data.

#### 3.2.2. Synthesis of 5-Pyrazolone Compounds (3e–g) Derived from Phenylhydrazine Hydrochloride

The 5 mmol of β-keto ester, 5 mmol of an appropriately substituted phenylhydrazine hydrochloride in 10 mL of absolute methyl alcohol were refluxed in an oil bath (reaction times: For **3e** 8h, for **3f** 16 h, for **3g** 12 h). After the reaction was complete, the mixture was evaporated. The residue was purified by using column chromatography (5:2 petroleum ether: ethyl acetate).

*The 5-hydroxy-3-(4-nitrophenyl)-1-(p-tolyl)-1H-pyrazole* (**3e**). The title compound was synthesized according to the general procedure using 5 mmol (1.19 g) of ethyl 4-nitrobenzoyl acetate and 5 mmol (0.81 g) of p-tolylhydrazine hydrochloride. SiO_2;_ petroleum ether/ethyl acetate 5:2- *R_f_*: 0.32. Yellow solid, yield: 0.797 g (54%), mp: 220–221 °C. Elemental analysis; calculated for C_16_H_13_N_3_O_3_; C, 65.08; H, 4.44; N, 14.23; found: C, 64.97; H, 4.54; N, 14.17. IR (ATR, cm^-1^): 3093, 2924, 2850, 2615–2441 (enolic OH), 1562 (C=N), 1415, 1350 (NO_2_) 1381, 1238, 1145, 1111, 1076, 1018, 964, 852, 744. The ^1^H-NMR (400 MHz, DMSO-*d_6_*): δ 12.01(s, 1H, -O***H***), 8.28 (d, 2H, nitrophenyl C***H***, *J* = 8.8 Hz), 8.10 (d, 2H, nitrophenyl C***H***, *J* = 8.8 Hz), 7.71(d, 2H, phenyl C***H***, *J* = 8.4 Hz), 7.32 (d, 2H, phenyl C***H***, *J* = 8.4 Hz), 6.20 (s, 2H, pyrazolone C***H***,), 2.35 (s, 3H, -C***H***_3_). The ^13^C-NMR (100 MHz, DMSO-*d_6_*,): δ 154.5 (***C***=O pyrazolone), 147.7, 147, 140.4, 136.6, 136.1, 129.9 (2xAr ***C***H), 124.5 (2xAr ***C***H), 126.3 (2x Ar ***C***H), 121.9 (2xAr ***C***H), 86.5 (C_4_ pyrazolone), 21 (-***C***H_3_). LC-MS/MS *m/z* 294.4 [M-1]^+^, 295.4 [M]^+^.

*The 1-(4-bromophenyl)-3-(4-nitrophenyl)-1H-pyrazole-5(4H)-one* (**3f**). The title compound was synthesized according to the general procedure using 5 mmol (1.19 g) of ethyl 4-nitrobenzoyl acetate and 5 mmol (1.12 g) of 4-bromophenylhydrazine hydrochloride. SiO_2_; petroleum ether /ethyl acetate 5:2- *R_f_*: 0.12. Yellow solid, yield: 0.450 g (25%), mp: 182–183 °C. Elemental analysis; calculated for C_15_H_10_BrN_3_O_3_: C, 50.02; H, 2.80; N, 11.67; found: C, 49.86; H, 2.83; N, 11.55. IR (ATR, cm^-1^): 3105, 2920, 2850, 1705 (C=O), 1589–1485 (C=N & C=C), 1338 (-NO_2_), 1315, 1296, 1157, 1122, 1068, 1002, 952, 821, 748 (C-Br). The ^1^H-NMR (400 MHz, CDCl_3_): δ 8.36 (d, *J*:9.2 Hz, 2H, nitrophenyl C***H***), 7.97 (d, *J*:8.8 Hz, 2H, nitrophenyl C***H***), 7.89 (d, *J*:9.2 Hz, 2H, bromophenyl C***H***), 7.56 (d, *J*: 9.2 Hz, 2H, bromophenyl C***H***), 3.92 (s, 2H, pyrazolone C***H***). The ^13^C-NMR (100 MHz, CDCl_3_,): δ 169.7 (***C***=O), 152.5, 148.9, 136.8, 136.2, 132.0 (2xAr ***C***H), 126.7 (2xAr ***C***H), 124.3 (2xAr ***C***H), 120.5, 118.8 (2xAr ***C***H), 39.32 (-***C***H_3_). LC-MS/MS *m/z* 359.9 [M]^+^, 361.4 [M+2]^+^.

*The 1-(4-chlorophenyl)-3-(4-nitrophenyl)-1H-pyrazole-5(4H)-one* (**3g**). The title compound was synthesized according to the general procedure using 5 mmol (1.19 g) of ethyl 4-nitrobenzoyl acetate and 5 mmol (0.91 g) of 4-chlorophenylhydrazine hydrochloride. SiO_2_; petroleum ether/ethyl acetate 5:2- *R_f_*: 0.2. Orange solid, yield: 1.105 g (70%), mp: 185–186 °C. Elemental analysis; calculated for C_15_H_10_ClN_3_O_3_: C, 57.07; H, 3.19; N, 13.87; found: C, 56.79; H, 3.29; N, 13.87. IR (ATR, cm^-1^): 3093, 2916, 2850, 1705 (C=O), 1593–1485 (C=N & C=C), 1340 (-NO_2_), 1315, 1276, 1157, 1122, 1087, 1006, 952, 825, 748 (C-Cl). The ^1^H-NMR (400 MHz, CDCl_3_): δ 8.36 (d, *J*: 9.2 Hz, 2H, nitrophenyl C***H***), 7.97 (d, *J*: 8.8 Hz, 2H, chlorophenyl C***H***), 7.94 (d, *J*: 3.2 Hz, 2H, chlorophenyl C***H***), 7.41 (d, *J*: 3.2 Hz, 2H, nitrophenyl C***H***), 3.92 (s, 2H, pyrazolone C***H***). The ^13^C-NMR (100 MHz, CDCl_3_): δ, 169.7 (***C***=O), 152.4, 148.9, 136.3, 136.2, 131, 129.1 (2xAr ***C***H), 126.7 (2xAr ***C***H), 124.3 (2xAr ***C***H), 120.2 (2xAr ***C***H), 39.3 (-CH_3_). LC-MS/MS *m/z* 314.2 [M-1]^+^; 316.2 [M+1]^+^.

*The 1-(4-bromophenyl)-3-methyl-1H-pyrazole-5(4H)-one* (**3h**). The 2.25 mmol (0.3 mL) of ethyl acetoacetate and 3 mmol (0.7 g) of 4-bromophenylhydrazine hydrochloride in 5 mL of absolute ethanol were allowed to stir at room temperature for 24 h. After the reaction was complete, the mixture was evaporated. The residue was purified by using column chromatography. SiO_2_; petroleum ether/ethyl acetate 5:2- *R_f_*: 0.18. Cream solid, yield: 0.308 g (54%), mp: 170–172 °C ([13]; yellow oily compound). Elemental analysis; calculated for C_10_H_9_BrN_2_O: C, 47.46; H, 3.58; N, 11.07; found: C, 47.54; H, 3.54; N, 11.14. IR (ATR, cm^-1^): 3051, 2916, 2850, 2681–2522 (enolic OH), 1610, 1581, 1400 (C=N & C=C), 1361, 1330, 1222, 1149, 1076, 1006, 750, 624, 574 (C-Br). The ^1^H-NMR (400 MHz, CDCl_3_): δ 7.82 (d, *J* =9.2 Hz, 2H, Ar C***H***), 7.50 (d, *J* =8.8 Hz, 2H, Ar C***H***), 3.42 (s, 2H, pyrazolone C***H***), 2.22 (s, 3H, -C***H***_3_). The ^13^C-NMR (100 MHz, CDCl_3_): δ 170.5 (**C**=O), 156.6, 137.2, 131.8 (2 × Ar ***C***H), 120.2, 117.8 (2 × Ar ***C***H), 43.1 (-***C***H_2_), 17.1 (-***C***H_3_). LC-MS/MS *m/z* 251 [M]^+^; 253 [M+2]^+^.

#### 3.2.3. General Procedure for the Preparation of 4-Formyl-2-Pyrazoline-5-One (**4a**–**d**)

In a round flask, 1.15 mmol of pyrazolone was dissolved in 2 mL of DMF. After cooling to 0 °C in an ice bath, 1.28 mmol (0.12 mL) phosphoryl chloride (POCl_3_) was added dropwise for 15 min while the temperature remained between 0–20 °C. After the addition was complete, the reaction mixture was heated in water vapor at 85 °C for 1.5 h. After the reaction was completed, 10 mL of the ice–water mixture was poured into the room-temperature mixture. The reaction mixture was stirred overnight. The aqueous mixture was extracted with chloroform (3 × 10 mL), then dried with Na_2_SO_4_, and the solvent was evaporated. The solids were crystallized in ethanol.

*The 3-Methyl-1-phenyl-4-formyl-2-pyrazoline-5-one* (**4a**). It was synthesized according to the literature. Yellow solid, yield: 0.116 g (50%), mp: 177–179 °C ([35]; 175–176 °C). Compounds **4a** and **4b** were synthesized according to the literature [35], and their FTIR, and ^1^H and ^13^C NMR spectra corresponded to the previously reported data.

*The 3-Isopropyl-1-phenyl-4-formyl-2-pyrazoline-5-one* (**4b**). It was synthesized according to the literature. Yellow solid, yield: 1.266 g (55%), mp: 155–157 °C ([35] 149–150 °C).

*The 1,3-diphenyl-4-formyl-2-pyrazoline-5-one* (**4c**). It was synthesized according to the literature. Yellow solid, yield: 0.499 g (45%), mp: 146–147 °C (According to the literature, when this product is crystallized from naphtha, the melting point is 146.5–148.5 °C, and when it is crystallized from 2-methoxyethanol again, the melting point increases to 248–249 °C.) [36]. Compound **4c** was synthesized according to the literature [36], and its FTIR, and ^1^H and ^13^C NMR spectra corresponded to the previously reported data.

*The 5-hydroxy-3- (4-nitrophenyl) -1-phenyl-4-formyl-1H-pyrazole* (**4d**). The 1.4 mmol (0.4 g) of compound **3d** was placed on a round 25-mL capacity flask and dissolved in 1 mL of DMF then cooled in ice to 0 °C. Then, 0.5 mL (5.4 mmol) of POCl_3_ was added dropwise for 15 min while the temperature was maintained between 0–20 °C. After the addition was complete, the reaction mixture was heated in water vapor at 85 °C for 3 h. After the reaction was completed, 10 mL of iced water was poured into the room-temperature mixture. The reaction mixture was stirred overnight. The aqueous mixture was extracted with dichloromethane (3 × 10 mL), then dried with Na_2_SO_4_, and the solvent was evaporated. Purification by silica gel column chromatography (petroleum ether/ethyl acetate 5:2) gave compound **4d** as an orange solid. The solid was recrystallized from ethanol, and collected by filtration to give 0.195 g (45%), as an orange solid, mp: 272–273 °C. Elemental analysis; calculated for C_16_H_11_N_3_O_4_: C, 62.14; H, 3.59; N, 13.59; found: C, 62.06; H, 3.50; N, 13.65. IR (ATR, cm^-1^): 3066, 2978, 2850, 2800, and 2742 (aldehyde CH), 1670 (C=O), 1597 (C=N), 1315 (-NO_2_), 1489, 1292, 1126, 1095, 1029. The ^1^H-NMR (400 MHz, CDCl_3_): δ 10.10 (s, 1H, -C***H***O), 9.90 (s, 1H, -O***H***), 8.34 (d, *J*: 8.4 Hz, 2H, nitrophenyl C***H***), 8.19 (d, *J*:8.4 Hz, 2H, nitrophenyl C***H***), 7.66–7.61 (m, 5H, Ar C***H***). The ^13^C-NMR (100 MHz, CDCl_3_,): δ 183, 150.9, 148.3, 137.2, 136.7, 135.7, 129.9 (2 × Ar ***C***H), 129.8 (2 × Ar ***C***H), 129.5, 125.4 (2 ×Ar ***C***H), 123.6 (2 × Ar ***C***H), 116.8. LC-MS/MS *m/z* 308.2 [M − 1]^+^.

#### 3.2.4. General Procedure for the Preparation of Urea-5-Pyrazolones (**5a–d**)

After 1 mmol of the compound 4-formyl-2-pyrazoline-5-one was dissolved in 10 mL of DMF, 10 mmol of urea was added to the mixture. The reaction mixture was heated at 85 °C in an oil bath for 6 h. After the reaction was completed, 20 mL of bi-distilled water was added to the mixture and allowed to stir for 10 min. The aqueous mixture was removed by extraction in chloroform (3 × 10 mL), dried with Na_2_SO_4_, and the solvent was evaporated. The solids were purified by column chromatography (5:2 petroleum ether: ethyl acetate).

*N-[(1,5-dihydro-3-methyl-5-oxo-1-phenyl-4H-pyrazol-4-ylidene)methyl]urea* (**5a**). SiO_2_; petroleum ether/ethyl acetate 5:2- *R_f_*: 0.52. Yellow solid, yield: 0.068 g (28%), mp: 230–232 °C (lit^23^; 228–229 °C). Elemental analysis; calculated for C_12_H_12_N_4_O_2_: C, 59.01; H, 4.95; N, 22.94; found: C, 58.93; H, 4.81; N, 23.07. IR (ATR, cm^-1^): 3348, 3278 (-NH_2_), 3201 (-NH), 3062, 1735 (C=O), 1670 (C=O), 1597 (C=N), 1558, 1489, 1377, 1253, 1161, 1045. The ^1^H-NMR (400 MHz, DMSO-*d_6_*): δ 10.8 (d, *J*:12.4 Hz, -N***H***), 8,21 (d, *J*: 12 Hz, urea-C***H***=C), 7.94 (d, *J*: 7.6 Hz, 2H, Ar C***H***), 7.79 (s, 2H, -N***H***_2_), 7.41 (t, *J*: 16 Hz, 2H, Ar C***H***), 7.15 (t, *J*: 14.8 Hz, H, Ar C***H***), 2.24 (s, 3H, -C***H***_3_). The ^13^C-NMR (100 MHz, DMSO-*d_6_*): δ 164.5, 153.1, 150.3, 143.5, 139.2, 129.3(2xAr ***C***H), 124.5, 118.1(2xAr ***C***H), 104.8, 12,9 (-***C***H_3_). LC-MS/MS: *m/z* 243.2 [M-1]^+^; 244.3 [M]^+^.

*N-[(1,5-dihydro-1-phenyl-5-oxo-3-(isopropyl)-4H-pyrazol-4-ylidene)methyl]urea* (**5b**). SiO_2_; petroleum ether /ethyl acetate 5:2- *R_f_*: 0.16. Yellow solid, yield: 0.089 g (33%), mp: 179–181 °C. Elemental analysis; calculated for C_14_H_16_N_4_O_2_: C, 61.75; H, 5.92; N, 20.58; found: C, 61.88; H, 5.86; N, 20.77. IR (ATR, cm^−1^): 3340–3270 (-NH_2_), 3190 (-NH), 3062, 2970, 2873, 1701 (C=O), 1662 (C=O), 1577 (C=N), 1489, 1377, 1253, 1161, 1014. The ^1^H-NMR (400 MHz, DMSO-*d_6_*): δ 10.87 (d, *J*: 12 Hz, 1H, -N***H***), 8.25 (d, *J*:12 Hz, 1H, -C***H***=C), 7.97 (d, *J*: 7.6 Hz, 2H, Ar C***H***), 7.85 (s, 2H, -N***H***_2_), 7.42 (t, *J*:16 Hz, 2H, Ar C***H***), 7.16 (t, *J*: 14.8 Hz, 1H, Ar C***H***), 3.11 (m, 1H, -C***H***), 1.26 (d, *J*: 7.2 Hz, 6H,-CH(C***H***_3_)_2_). The ^13^C-NMR (100 MHz, DMSO-*d_6_*): δ 164.8 (***C***=O), 157.8 (***C***=O), 143, 139.2, 129.3 (2 × Ar ***C***H), 124.6, 118.2 (2 × Ar ***C***H), 103.2, 26.8 (-***C***H), 21.6 (-CH(***C***H_3_)_2_). LC-MS/MS: *m/z* 271.2 [M-1]^+^ 272.9 [M]^+^.

*N-[(1,5-dihydro-1,3-diphenyl-5-oxo-4H-pyrazol-4-ylidene)methyl]urea* (**5c**). SiO_2_; petroleum ether/ethyl acetate 5:2- *R_f_*: 0.2. Yellow solid, yield: 0.110 g (36%), mp: 220–223 °C. Elemental analysis; calculated for C_17_H_14_N_4_O_2_: C, 66.66; H, 4.61; N, 18.29; found: C, 66.90; H, 4.50; N, 18.50. IR (ATR, cm^-1^): 3421, 3278 (-NH_2_), 3201 (-NH), 3059, 1759 (C=O), 1658 (C=O), 1597 (C=N), 1477, 1373, 1265, 1172, 1111. The ^1^H-NMR (400 MHz, DMSO-*d_6_*): δ 11.11 (d, *J*: 12.4 Hz, 1H, -N***H***), 8.31 (d, *J*: 12.4 Hz, 1H, -C***H***=C), 8.06 (d, *J*: 8 Hz, 2H, Ar C***H***), 7.88 (s, 2H, -N***H***_2_), 7.75 (d, *J*: 7.6 Hz, 2H, Ar C***H***), 7.55–7.47 (m, 5H, Ar C***H***), 7.22 (t, *J*: 14.4 Hz, 1H, Ar C***H***). The ^13^C-NMR (100 MHz, DMSO-*d_6_*): δ 164.8(***C***=O), 152.9 (***C***=O), 150.9, 145.2, 139, 131.4, 130.1 (2 × Ar ***C***H), 129.5 (2 × Ar ***C***H), 129.4, 128.1 (2 × Ar ***C***H), 125.1, 118.7 (2 × Ar ***C***H), 102.7. LC-MS/MS: *m/z* 305.2 [M-1]^+^ 306.5 [M]^+^.

*N-[(1,5-Dihydro-1-phenyl-5-oxo-3-(4-nitrophenyl)-4H-pyrazol-4-ylidene)methyl]urea* (**5d**). SiO_2_; petroleum ether/ethyl acetate 5:2- *R_f_*: 0.08. Orange solid, yield: 0.109 g (31%), mp: >250 °C decomposition. Elemental analysis; calculated for C_17_H_13_N_5_O_4_: C, 58.12; H, 3.73; N, 19.93; found: C, 58.26; H, 3.70; N, 20.16. IR (ATR, cm^−1^): 3294–3124 (-NH_2_ & -NH), 3051, 1662 (C=O), 1643 (C=O), 1597 (C=N), 1512, 1381, 1195, 1103, 1323 (-NO_2_). The ^1^H-NMR (400 MHz, DMSO-*d_6_*): δ, 9.69 (d, *J*: 14.8 Hz, 1H, -N***H***), 9.47 (s, 2H, -N***H***_2_), 8.34 (d, *J*: 8.8 Hz, 2H, nitrophenyl C***H***), 8.21–8.15 (dd, *J_1_*: 8.4, *J_2_*: 8 Hz 1H, -C***H***=C), 8.09 (d, *J*: 8.4 Hz, 2H, Ar C***H***), 8.03 (d, *J*: 8.8 Hz, 2H, nitrophenyl C***H***), 7.45 (t, *J*:16 Hz, 2H, Ar C***H***), 7.19 (t, *J*: 14.8 Hz, 1H, Ar C***H***). The ^13^C-NMR (100 MHz, DMSO-*d_6_*): δ 165.8 (***C***=O), 154.9 (**C**=O), 147.8, 147.7, 139.5, 138.8, 129.2 (2 × Ar ***C***H), 12 8.9 (2 × Ar ***C***H), 124.4 (2 × Ar ***C***H), 118.8 (2 × Ar ***C***H), 97.3. LC-MS/ MS: *m/z* 353.4 [M+2]^+^ 374.6 [M+Na]^+^.

### 3.3. Biological Assays

#### 3.3.1. Cell Cultures

Human skin cancer cell lines A431 (highly invasive, metastatic) and HaCaT (noncancerous, immortalized) were maintained as a monolayer culture in Dulbecco’s Modified Eagle Medium (DMEM) supplemented by 10% fetal bovine serum (FBS). Gentamicin and fungizone were added to prevent contamination. Cell cultures were kept in a CO_2_ incubator at 37 °C under 5% CO_2_. Cells were subcultured every third day (1:10 ratio). For subcultures, cells were rinsed with Trypsin containing ethylenediaminetetraacetic acid (Trypsin-EDTA) after the cell culture medium was removed. After 5 min of incubation in Trypsin-EDTA at 37 °C, cells were dislodged by gentle aspiration. The cells were transferred into a 15 mL conical tube and centrifuged at 200× *g* for 2 min. The pellet was resuspended in a minimal volume of complete growth medium (37 °C). After determining the total number of cells and percent viability using the Countess^®^ Automated Cell Counter (Invitrogen, Grand Island, NY, USA), growth media was added to achieve the required cell concentration. Cells then were diluted to the seeding density in a new cell culture flask. The flasks were returned the cells to the incubator.

#### 3.3.2. Cell Viability Assay

Human cancerous A431 and HaCaT keratinocytes were seeded in 96-well plates (5000 cells per well) and incubated in standard cell culture medium overnight. Compounds were dissolved in DMSO to prepare a 10 mM stock solution and were further diluted in cell culture medium to prepare the treatment doses. The following day, the culture medium was removed, and the cells were treated with 0.0001, 0.001, 0.005, 0.01, 0.05, 0.1, 0.5, and 1 mM compound for each chemical for 24, 48, 72, and 96 h. The control group was treated with DMSO (0.1% of the total culture volume). At the end of the corresponding treatment time, MTT dye (5 mg/mL, Sigma, St. Louis, MO, U.S.A.) was added to each well, and cells were incubated in a cell culture incubator. After 4 h of incubation in MTT, the medium was removed, and formazan crystals were dissolved in 100 µl of DMSO (Sigma, USA). Optical density was measured at 570 nm wavelength on a microplate reader. The following Equation (1) was used to determine the percentage cell viability:

(1) $$\mathrm{Cell}\;\mathrm{Viability}\;(\%)=\;\frac{A_{\mathrm{sample}}}{A_{\mathrm{control}}}\times 100$$

The effects of the compounds on cell viability of cancerous and noncancerous cell lines were compared by using the General Linear Model. To minimize the effect of chance variance and improve accuracy, all experiments were performed in triplicates for each condition and were repeated twice.

#### 3.3.3. Cell Migration Assay

A straight line was drawn to the bottom of 12-well plates by using an ultra-fine Sharpie. HaCaT and A431 cells were seeded in marked 12-well plates in a serum-free medium containing 2 µM colcemid to 80% confluence. After overnight incubation, two scratch wounds per well were made by scraping the cell monolayer by using a p2 pipet tip. Wells were washed with 37 °C phosphate buffered saline (PBS) three times. Two mL of the medium containing 1 μM (micromolar) of the corresponding compound and 2 µM colcemid was added onto the cells. Cells were visualized and photographed under a microscope to confirm the formation of the scratch wound. After 12 h of incubation in the 37 °C incubator, cells were removed from the incubator, and wounds were photographed every two hours for the following 12-h period. At T_24_, the medium was aspirated from the wells, and the cells were fixed with 0.5 mL of ice-cold methanol at −20 °C for 10 min. Following this, methanol was aspirated from the plates, and 0.5 mL of crystal violet solution was added onto the cells. The cells were incubated with crystal violet at room temperature for 10 min. At the end of 10 min, the stain was removed and the cells were rinsed 4–5 times in deionized water. Plates were dried at room temperature, and images were taken under a 10x objective. The area of cell migration over time was quantified using ImageJ software. To minimize the effect of chance variance and improve accuracy, all experiments were performed in triplicate wells for each condition. Four images at the intersection of scratch wounds and premarked lines were taken per well. The migration distance was calculated based on the width of the wounds. The widths of the 12 areas were averaged per compound. The average for each compound was normalized to its control wells. The effects of the compounds on cell migration of cancerous and noncancerous cell lines were compared by using the General Linear Model.

#### 3.3.4. Effects of Compounds on H_2_O_2_-Induced Cytotoxicity

The antioxidant potential of the compounds was investigated by measuring the ability of the compounds to prevent H_2_O_2_-induced cell death. The 96-well plates were seeded with A431 and HaCaT cells with each well containing 5000 cells/well. After overnight incubation, the standard cell culture medium was replaced with the medium that contained a 10 µM compound. After 24 h incubation in the compounds, half of the cells were treated with 10 µM H_2_O_2_. The control group was treated with DMSO (0.1% of the total culture volume). At the end of two-hour treatment with H_2_O_2_, MTT dye (5 mg/mL, Sigma, St. Louis, MO, U.S.A.) was added to each well, and cells were incubated in cell culture incubator for 4 h. Following this, the medium was removed, and formazan crystals were dissolved in 100 µl of DMSO (Sigma, St. Louis, MO, U.S.A.). Optical density was measured at 570 nm wavelength on a microplate reader. Equation (1) was used to determine the percentage of cell viability. The effects of the compounds on cell viability of the cancerous and noncancerous cell lines were compared by using the General Linear Model. To minimize the effect of chance variance and improve accuracy, all experiments were performed in triplicates for each condition.

### 3.4. Statistical Analysis

Statistical analyses of the effects of compounds on cell viability, cell migration, and antioxidant capacity were determined using a General Linear Model (SPSS v26). For the cell viability with four time points as a within-subject factor, cancer versus no cancer cell lines as a between-subjects factor, and dose (vehicle plus seven concentrations) as another between-subjects factor (a 4 × 2 × 8 mixed factor) design. For the cell migration assay with seven time points as a within-subject factor, cancer versus no cancer cell lines as a between-subjects factor, and dose (vehicle plus one concentration) as another between-subjects factor (a 7 × 2 × 2 mixed factor) design. For the antioxidant capacity assay with the presence of H_2_O_2_ as a within-subject factor, cancer versus no cancer cell lines as a between-subjects factor, and dose (vehicle plus one concentration) as another between-subjects factor (a 2 × 2 × 2 mixed factor design).

## 4. Conclusions

The synthesis part of our study consisted of three stages. In the first step, three new substitutes 5-pyrazolone were synthesized by condensation of 4-substituted phenylhydrazines with suitable β-keto esters. Subsequently, the carbon 4 of the pyrazolone ring was formulated to form the corresponding pyrazolone ring aldehydes. In the last stage, pyrazolone-CH=NCONH_2_ tautomerized by condensation of 4-carbaldehyde-5-pyrazolone compounds with urea yielded four new substituted-pyrazole-4-ylidenemethylurea compounds in the form of pyrazolone=CH-NHCONH_2_. The structures of the nine new compounds were characterized by elemental analysis, FTIR, ^1^H and ^13^C NMR, and LC/MS-MS spectroscopic methods. In contrast, the in vitro biological potentials were evaluated by biological tests such as MTT, cell migration, and prevention of H_2_O_2_-induced cell death.

To evaluate the toxicity of the compounds on cancerous cells, both cancerous and noncancerous cells that were derived from the same tissue (tumorigenic A431 skin cancer cells and nontumorigenic HaCaT human keratinocytes) were studied simultaneously. When the efficacy of the compounds on cell viability was examined by MTT test, all compounds containing urea (**5a**–**d**) showed higher toxicity in cancerous A431 cells compared to noncancerous cells at all time points (*p* < 0.05). In particular, 1 mM **5a** decreased cell viability by approximately 90% in both A431 and HaCaT cells after 24 h of treatment (*p* < 0.05). Because of the lower toxicity of compounds **3e**, **3f**, **3h**, and **5a**–**d** to noncancerous cells, it requires further investigation of their pharmacological potential. Compounds **4d** and **3g** showed high toxicity to cancer-free cells; therefore, their effects on cell migration were not investigated.

By using cell scratch (wound healing) assay, we investigated whether the compounds inhibit the migration and invasion ability of human keratinocytes. Compound **3e**, **3f**, **5a**, and **5b** were observed to inhibit cell migration of cancerous keratinocytes (*p* < 0.05). In particular, the ability of **3e**, **5a**, and **5b** to reduce cancer metastasis, as it prevents migration of cancerous cells, since it increases the migration of cancer-free cells, was observed. Compound **3f** needs to be further investigated for its wound-healing capabilities.

To investigate the antioxidant capacity of the compounds, their ability to prevent cell death caused by H_2_O_2_ was investigated. However, four of the nine compounds prevented cell death caused by H_2_O_2_. The antioxidant capacity and the effects of these compounds on necrosis and apoptosis will be further investigated in future studies.

Our data show that some of these compounds have the potential to be considered as promising useful building blocks of drug design investigations that prevent cancer metastasis and promote wound healing. In addition, because the urea end of the pyrazolone ring has the potential to undergo different chemical modifications, we speculate that this ability may help these structures to exhibit additional pharmacological activities.

## Supplementary Materials

The following are available online. Figure S1–S9: The FTIR spectra of the synthesized compounds. Figure S10–18: The 1H-NMR spectra of the synthesized compounds. Figures S19–S27: The 13C NMR spectra of the synthesized compounds. Figure S28–S36: The LC-MS/MS spectra of the synthesized compounds. Figure S37–S45: The time-dependent cell viability plots. Figure S46: The time- and dose-dependent cell viability plots. Table S1: The elemental analysis results of the synthesized compounds.
