# Supplementary material for: Synthesis and Cytotoxic Evaluation of Some Substituted 5-Pyrazolones and Their Urea Derivatives
Source: Molecules. 2020 Feb 18;25(4):900. doi: 10.3390/molecules25040900 (PMC7070565; doi:10.3390/molecules25040900)
Supplement: Supplementary file 1 [file molecules-25-00900-s001.pdf]

SUPPLEMENTARY INFORMATION

Synthesis and cytotoxic evaluation of some substituted 5-pyrazolones and their urea derivatives

ALIYE GEDIZ ERTURK* and HILAL OMERUSTAOGLU

Department of Chemistry, Faculty of Science & Arts, Ordu University, 52200, Ordu, TURKEY
*E-mail: aerturk@att.net

Table of contents

The FT-IR spectra of the synthesized compounds (Figures S1-S9) 2

The ^{1}H NMR spectra of the synthesized compounds (Figures S10-S18 5

The FT-IR spectra of the synthesized compounds (Figures S1-S9) 2

The ^{13}C NMR spectra of the synthesized compounds (Figures S19-S27) 10

The LC-MS/MS spectra of the synthesized compounds (Figures S28-S36) 15

The Elemental analysis results of the synthesized compounds (Table S1) 20

Time dependent cell viability plots (Figures S37-S45) 21

Time and dose dependent cell viability plots (Figure S46) 23

FT-IR spectra

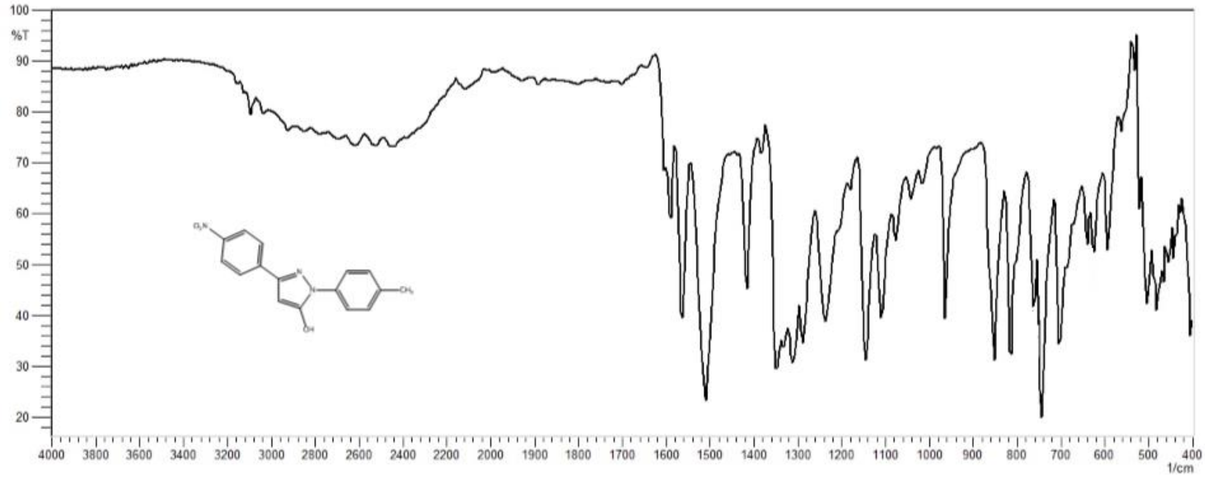

Figure S1. FTIR spectrum of 5-hydroxy-3-(4-nitrophenyl)-1-(p-tolyl)-1*H*-pyrazole **3e**.

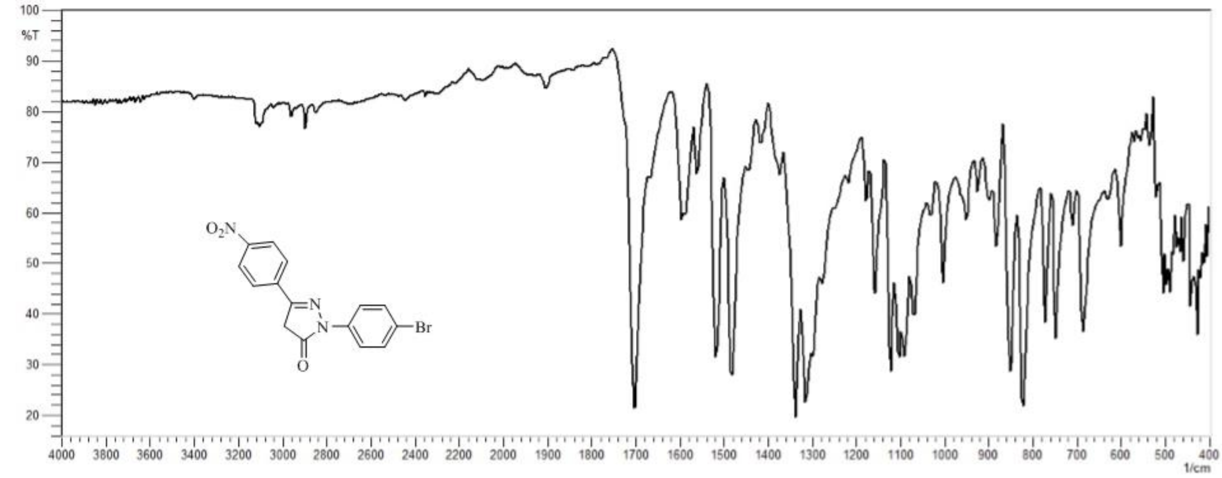

Figure S2. FTIR spectrum of 1-(4-bromophenyl)-3-(4-nitrophenyl)-1*H*-pyrazole-5(4*H*)-one **3f**.

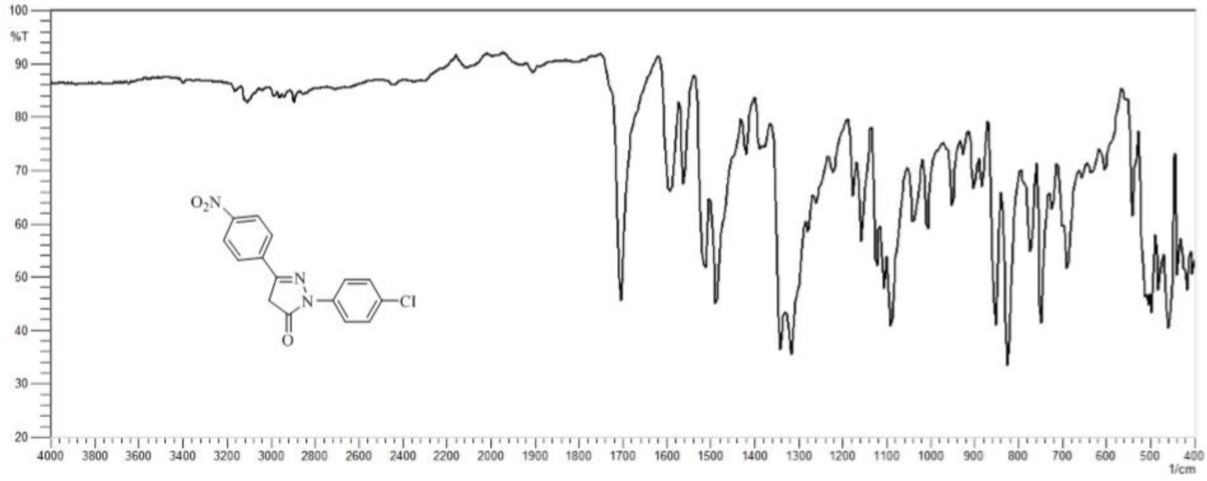

Figure S3. FTIR spectrum of 1-(4-chlorophenyl)-3-(4-nitrophenyl)-1*H*-pyrazole-5(4*H*)-one **3g**.

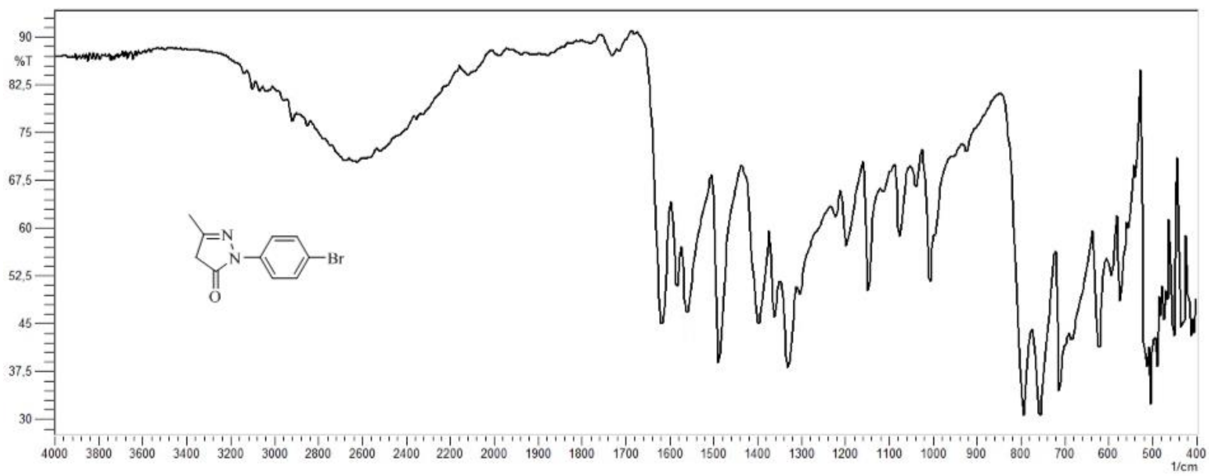

Figure S4. FTIR spectrum of 1-(4-bromophenyl)-3-methyl-1*H*-pyrazole-5(4*H*)-one **3h**.

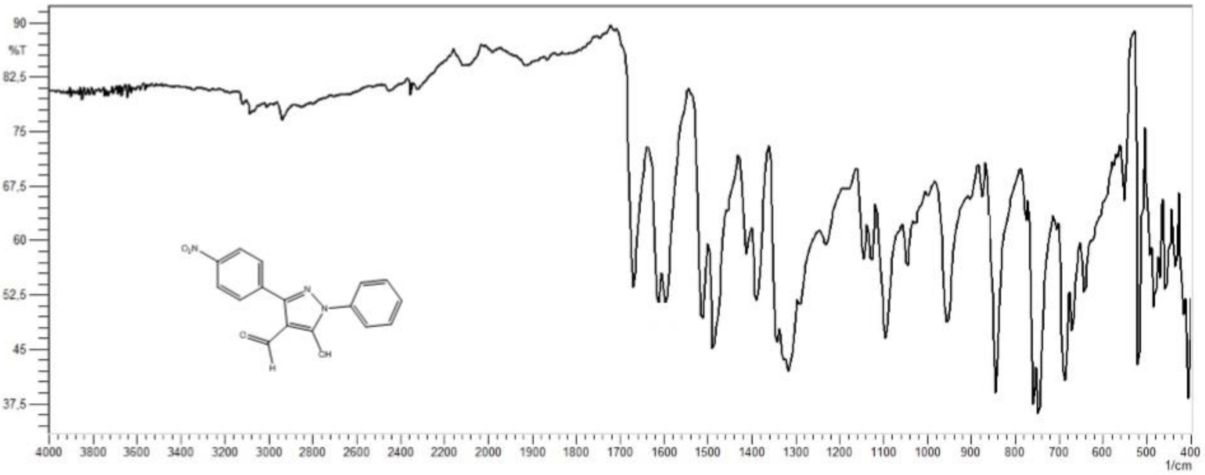

Figure S5. FTIR spectrum of 5-hydroxy-3-(4-nitrophenyl)-1-phenyl-4-formyl-1*H*-pyrazole **4d**.

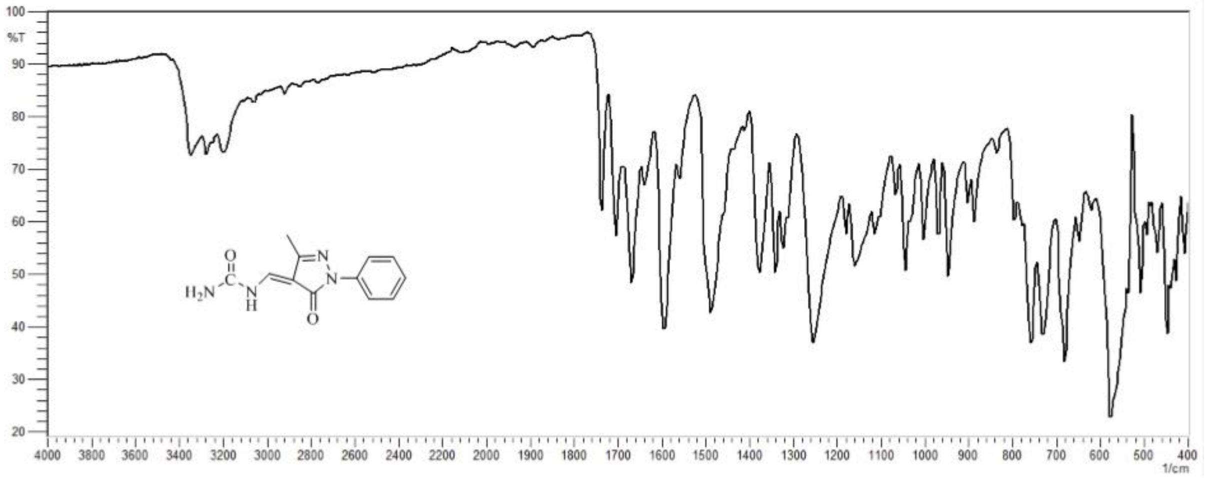

Figure S6. FTIR spectrum of N-[(1,5-dihydro-3-methyl-5-oxo-1-phenyl-4*H*-pyrazole-4-ylidene)methyl]urea **5a**.

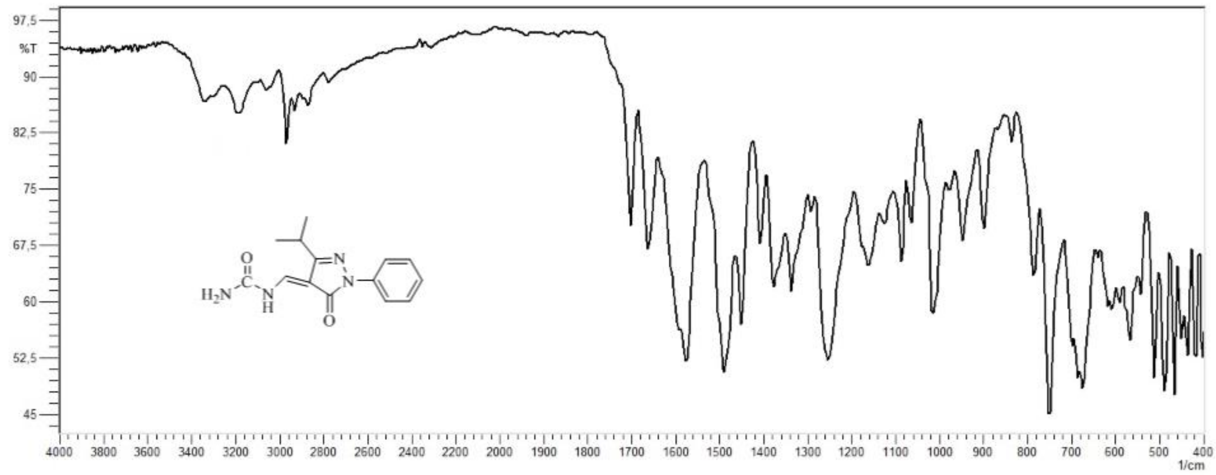

Figure 7. FTIR spectrum of N-[(1,5-dihydro-1-phenyl-5-oxo-3-(isopropyl)-4*H*-pyrazole-4-ylidene)methyl]urea **5b**.

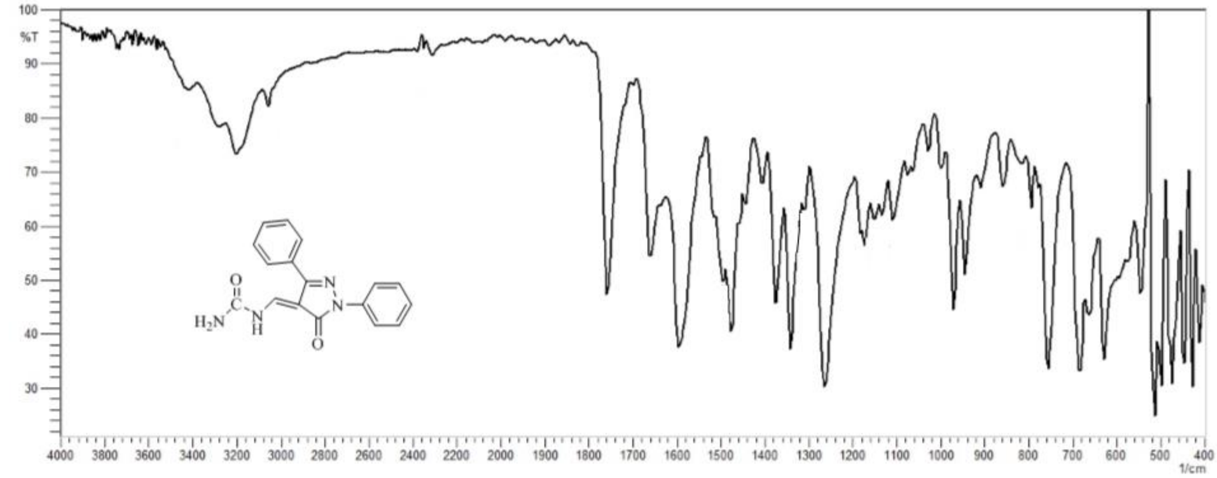

Figure 8. FTIR spectrum of N-[(1,5-dihydro-1,3-diphenyl-5-oxo-4*H*-pyrazole-4-ylidene)methyl]urea **5c**.

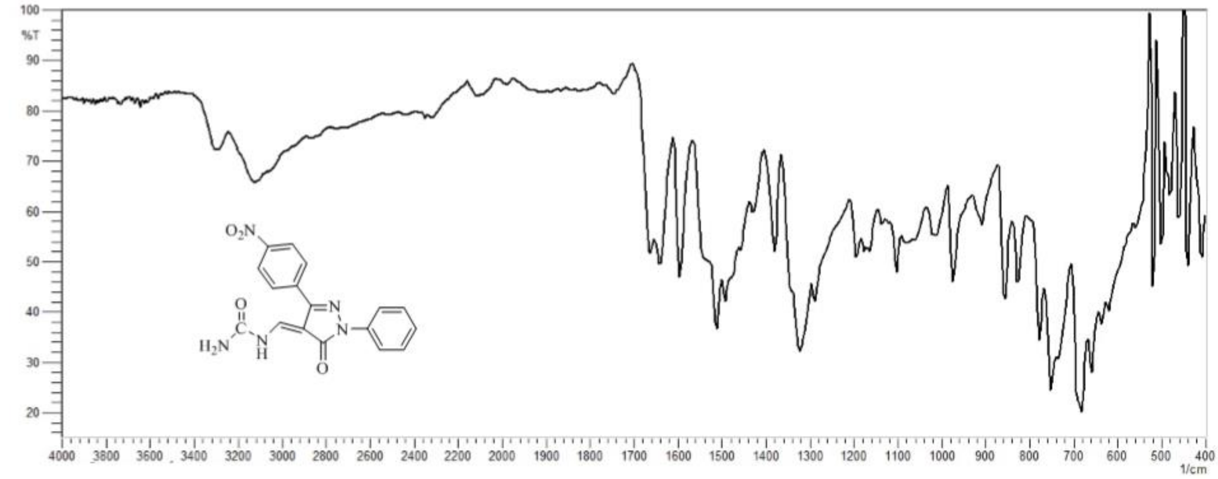

Figure 9. FTIR spectrum of N-[(1,5-dihydro-1-phenyl-5-oxo-3-(4-nitrophenyl)-4*H*-pyrazole-4-ylidene)methyl]urea **5d**.

¹H NMR spectra

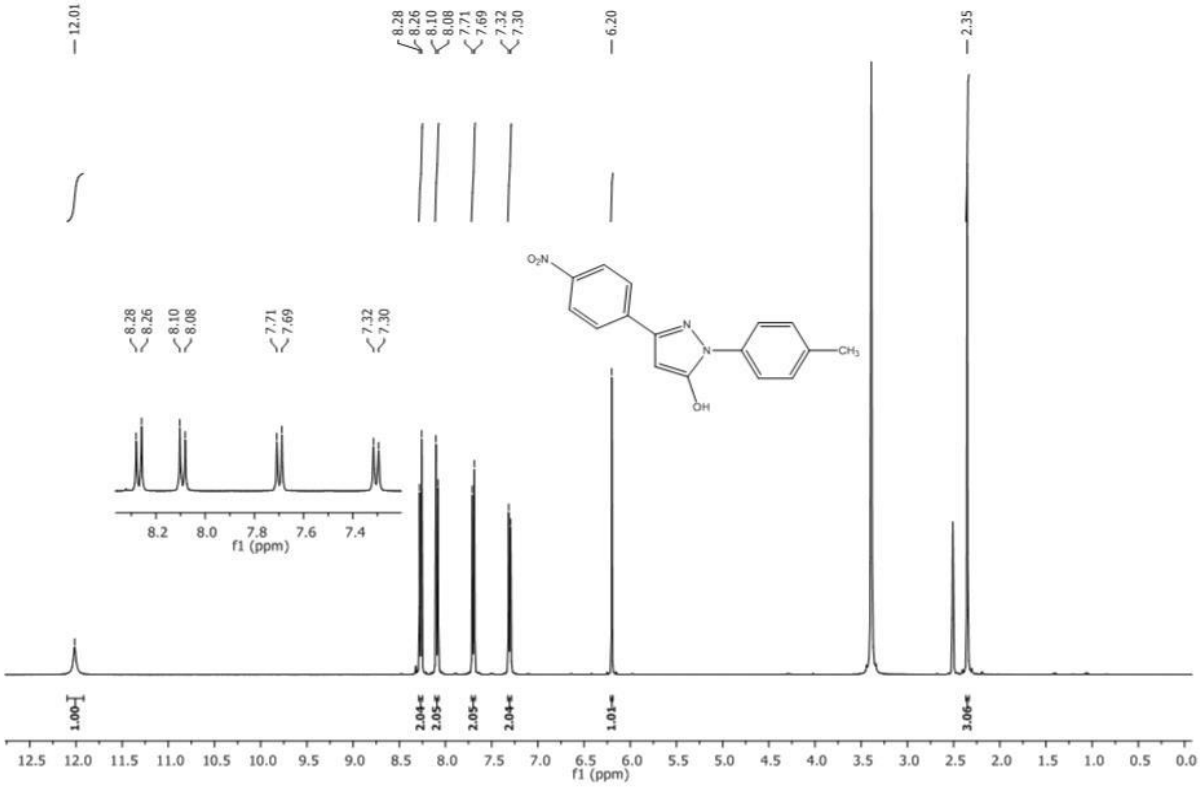

Figure S10. ¹H NMR spectrum in DMSO-d_6 of 5-hydroxy-3-(4-nitrophenyl)-1-(p-tolyl)-1*H*-pyrazole **3e**.

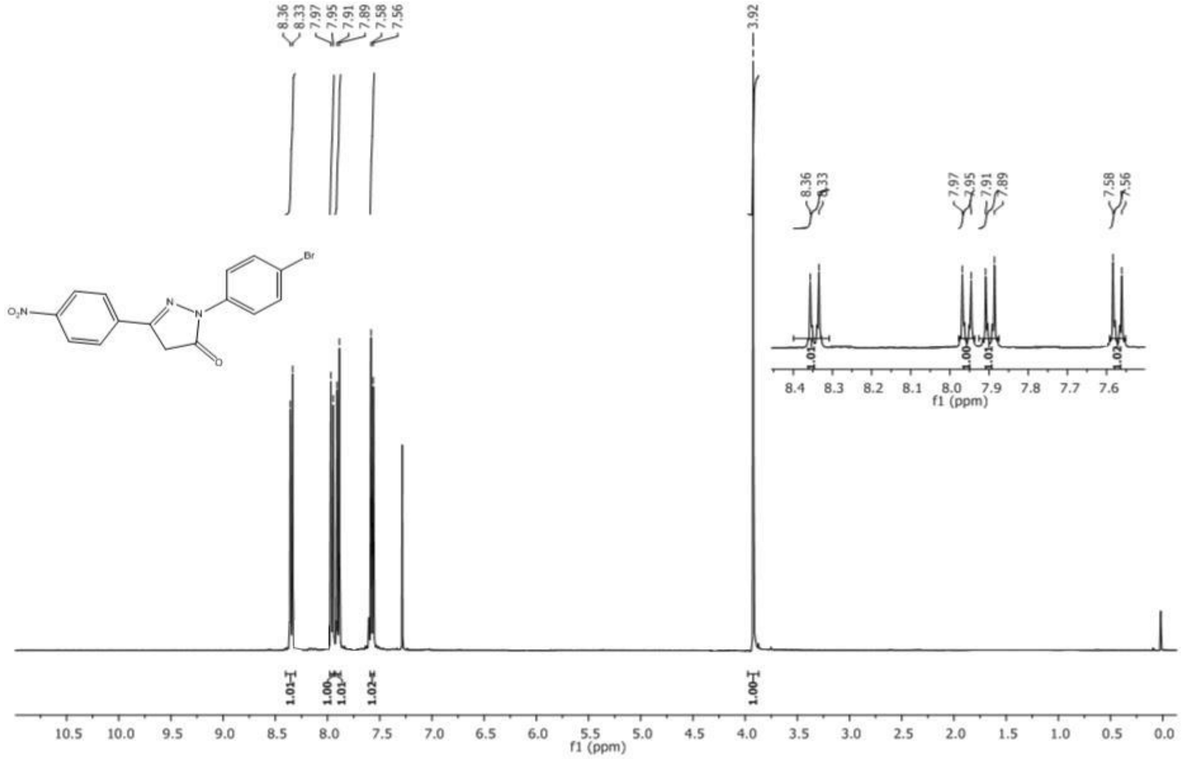

Figure S11. ¹H NMR spectrum in $CDCl_3$ of 1-(4-bromophenyl)-3-(4-nitrophenyl)-1*H*-pyrazole-5(4*H*)-one **3f**.

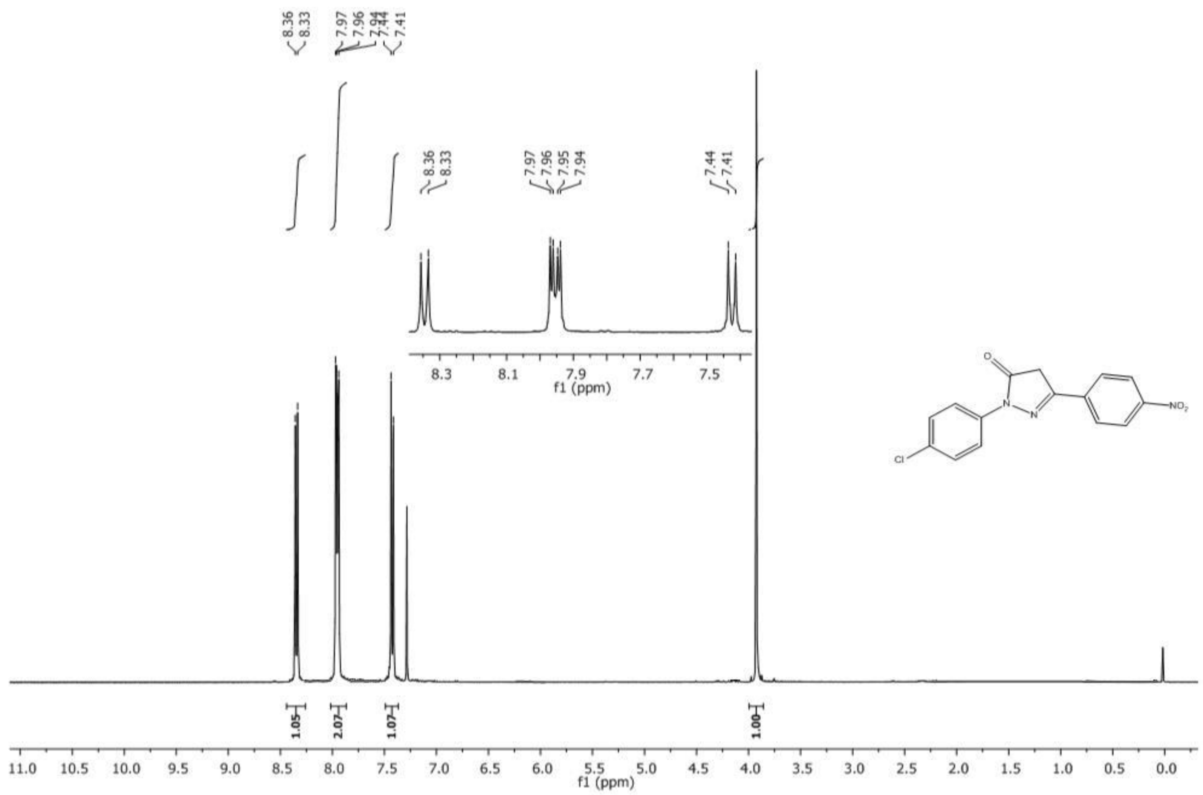

Figure S12. ^{1}H NMR spectrum in $CDCl_3$ of 1-(4-chlorophenyl)-3-(4-nitrophenyl)-1*H*-pyrazole-5(4*H*)-one **3g**.

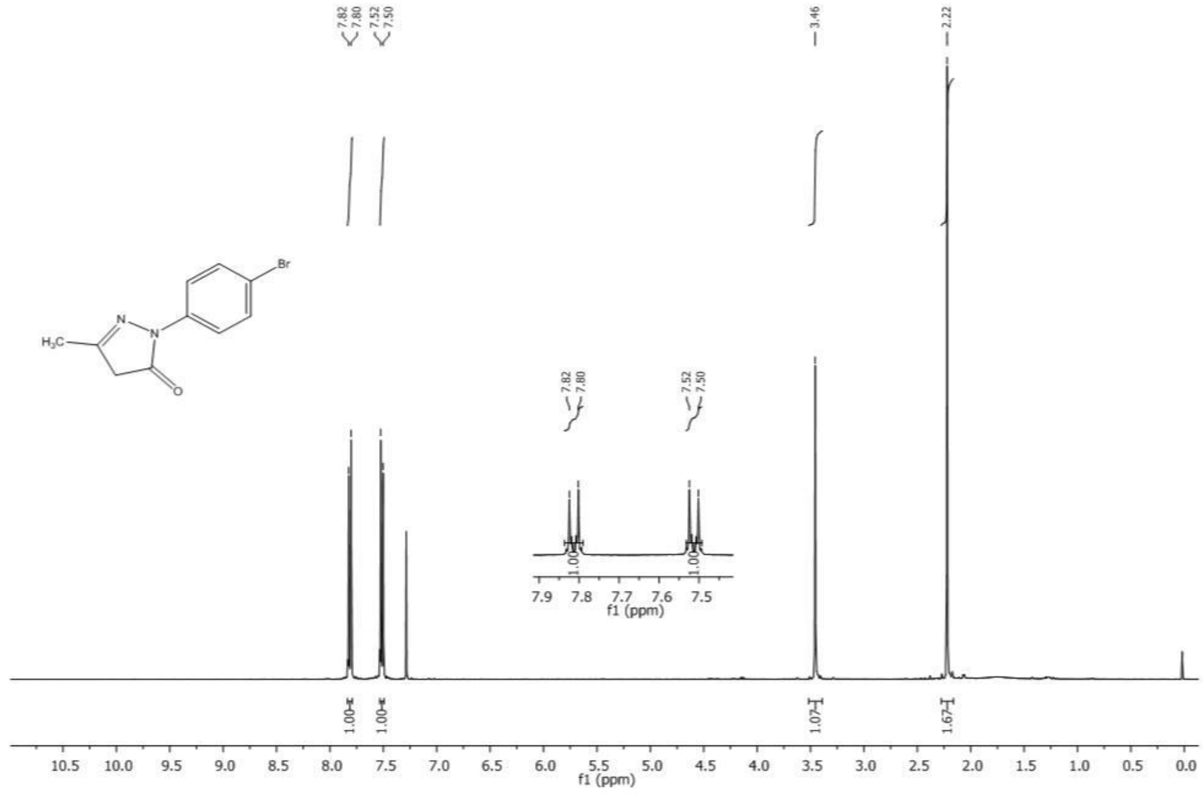

Figure S13. ^{1}H NMR spectrum in $CDCl_3$ of 1-(4-bromophenyl)-3-methyl-1*H*-pyrazole-5(4*H*)-one **3h**.

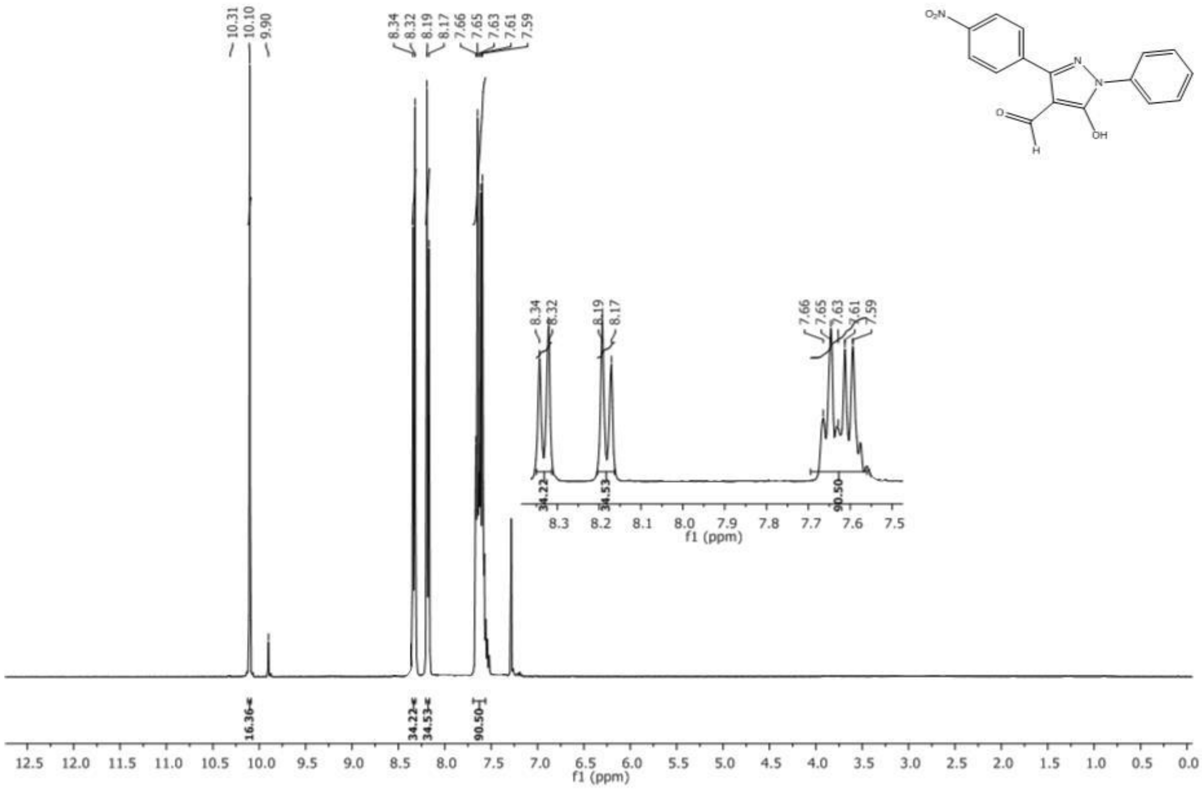

Figure S14. ^{1}H NMR spectrum in $CDCl_3$ of 5-hydroxy-3-(4-nitrophenyl)-1-phenyl-4-formyl-1*H*-pyrazole **4d**.

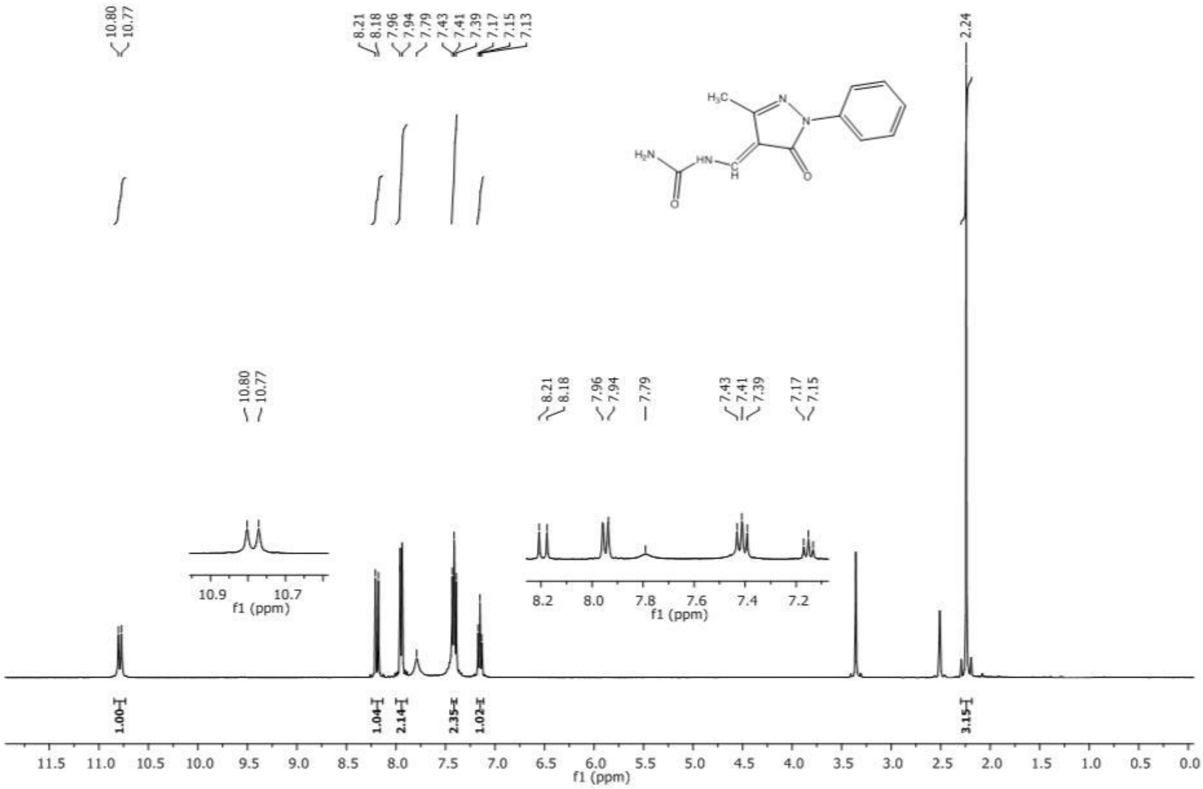

Figure S15. ^{1}H NMR spectrum in DMSO-d_6 of N-[(1,5-dihydro-3-methyl-5-oxo-1-phenyl-4*H*-pyrazole-4-ylidene)methyl]urea **5a**.

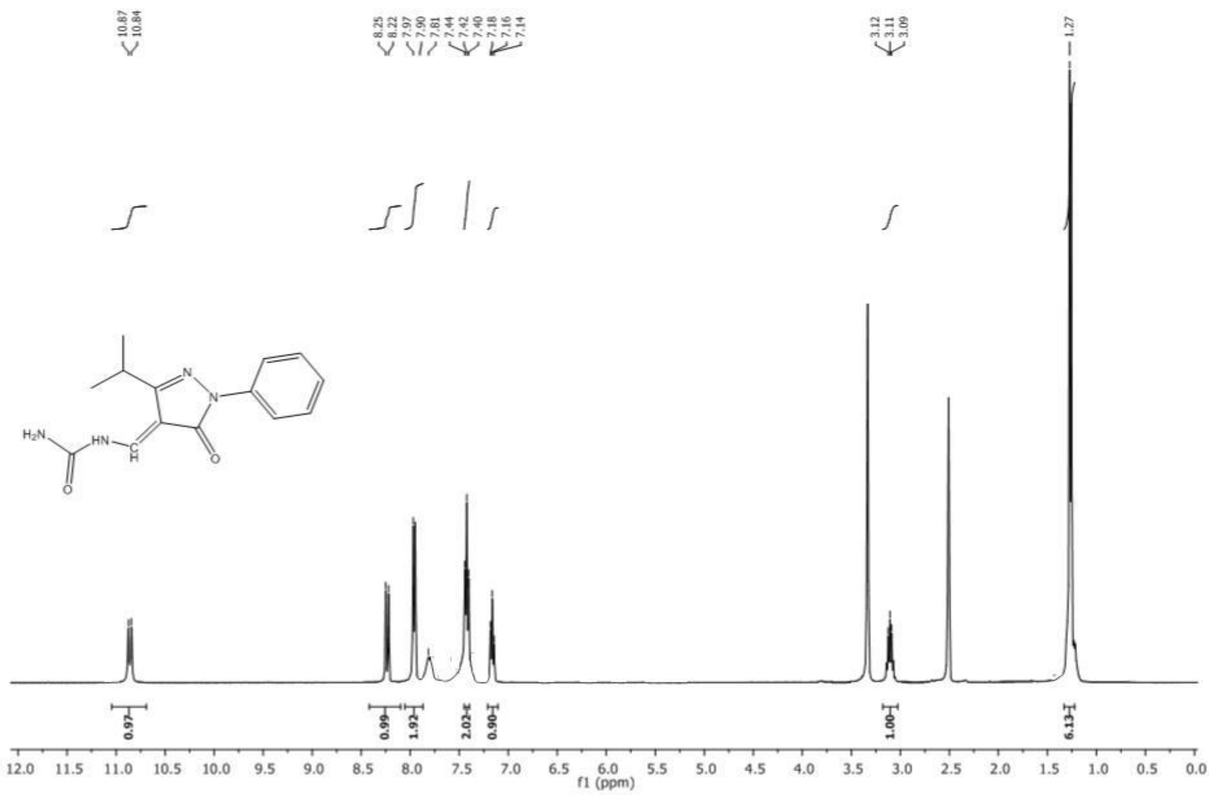

Figure S16. ^{1}H NMR spectrum in DMSO-d_6 of N-[(1,5-dihydro-1-phenyl-5-oxo-3-(isopropyl)-4*H*-pyrazole-4-ylidene)methyl]urea **5b**.

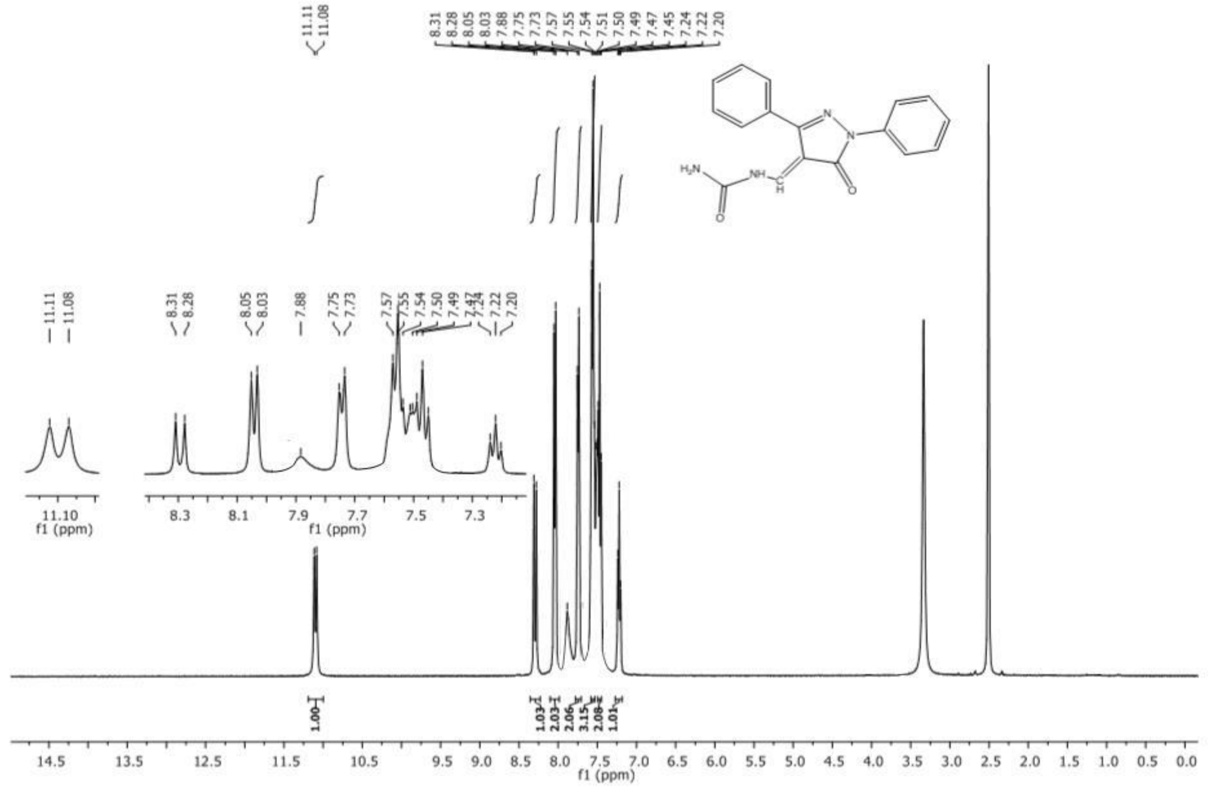

Figure S17. ^{1}H NMR spectrum in DMSO-d_6 of N-[(1,5-dihydro-1,3-diphenyl-5-oxo-4*H*-pyrazole-4-ylidene)methyl]urea **5c**.

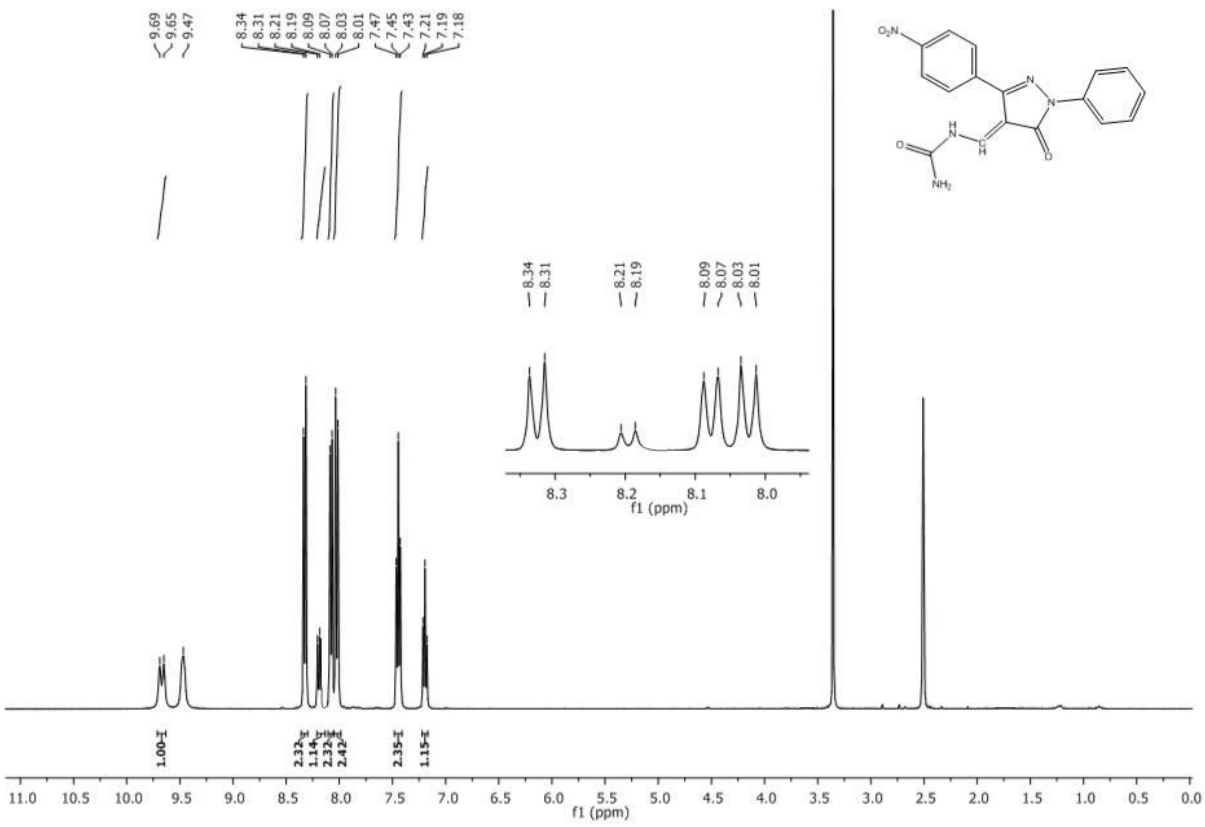

Figure S18. ^{1}H NMR spectrum in DMSO-d_6 of N-[(1,5-dihydro-1-phenyl-5-oxo-3-(4-nitrophenyl)-4*H*-pyrazole-4-ylidene)methyl]urea **5d**.

^{13}C NMR spectra

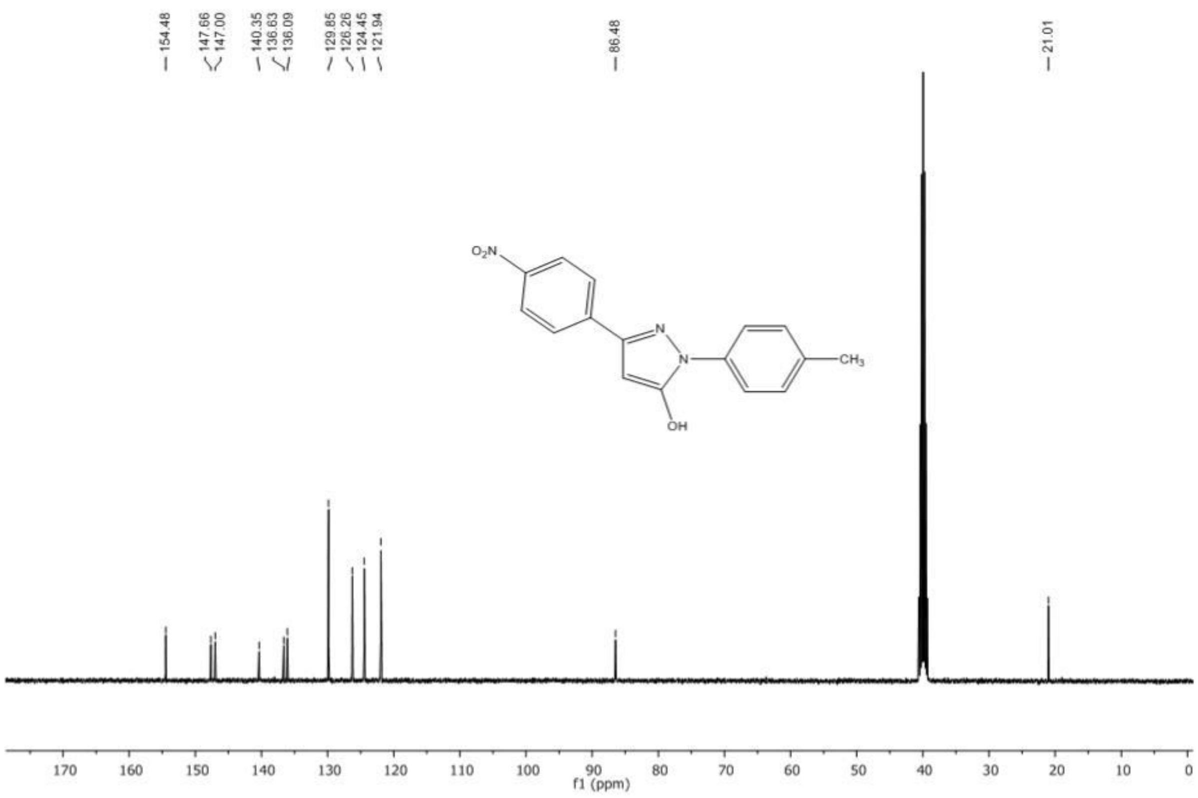

Figure S19. ^{13}C NMR spectrum in DMSO-d_6 of 5-hydroxy-3-(4-nitrophenyl)-1-(p-tolyl)-1*H*-pyrazole **3e**.

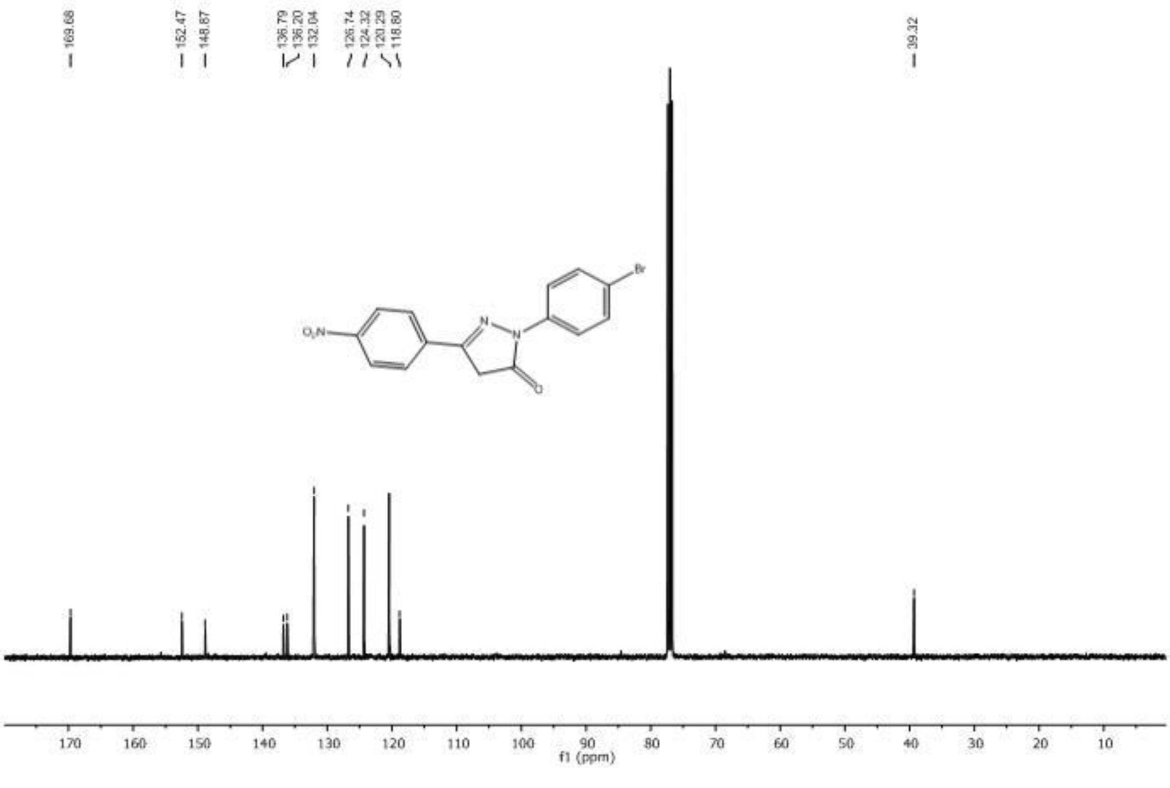

Figure S20. ^{13}C NMR spectrum in $CDCl_3$ of 1-(4-bromophenyl)-3-(4-nitrophenyl)-1*H*-pyrazole-5(4*H*)-one **3f**.

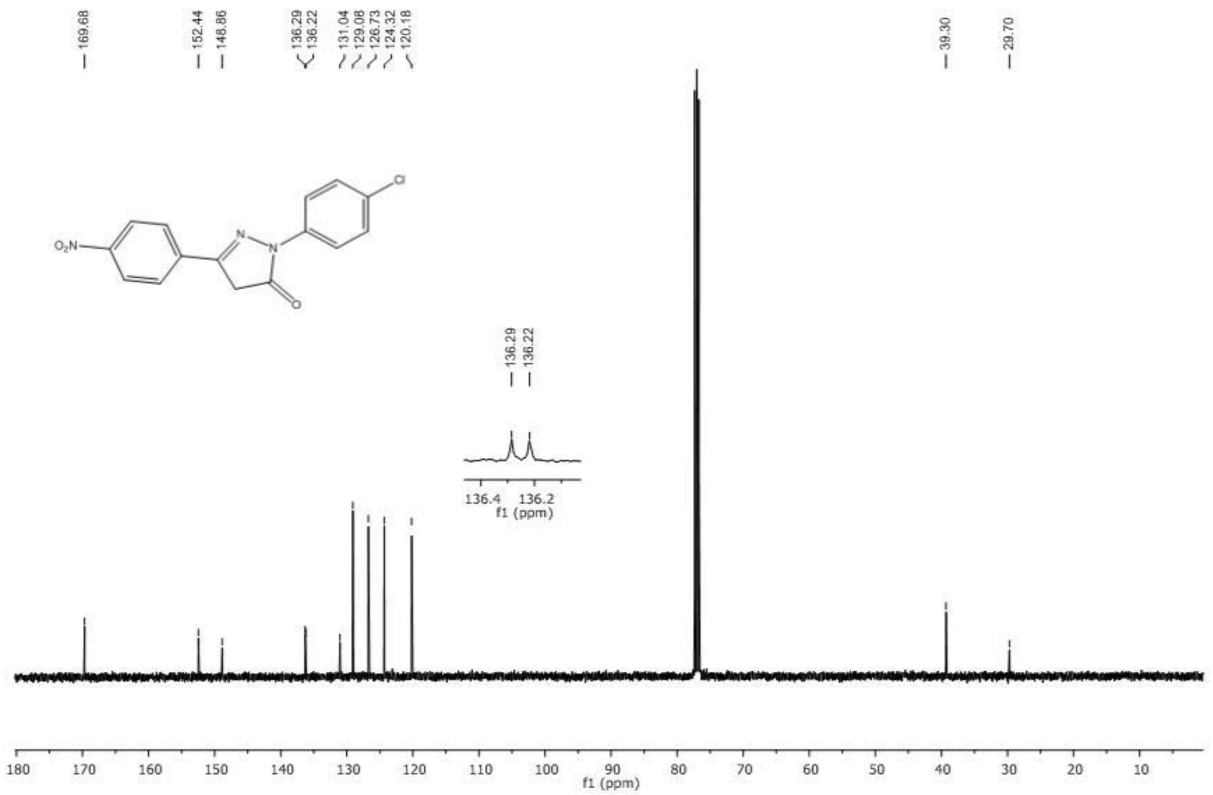

Figure S21. ^{13}C NMR spectrum in $CDCl_3$ of 1-(4-chlorophenyl)-3-(4-nitrophenyl)-1*H*-pyrazole-5(4*H*)-one **3g**.

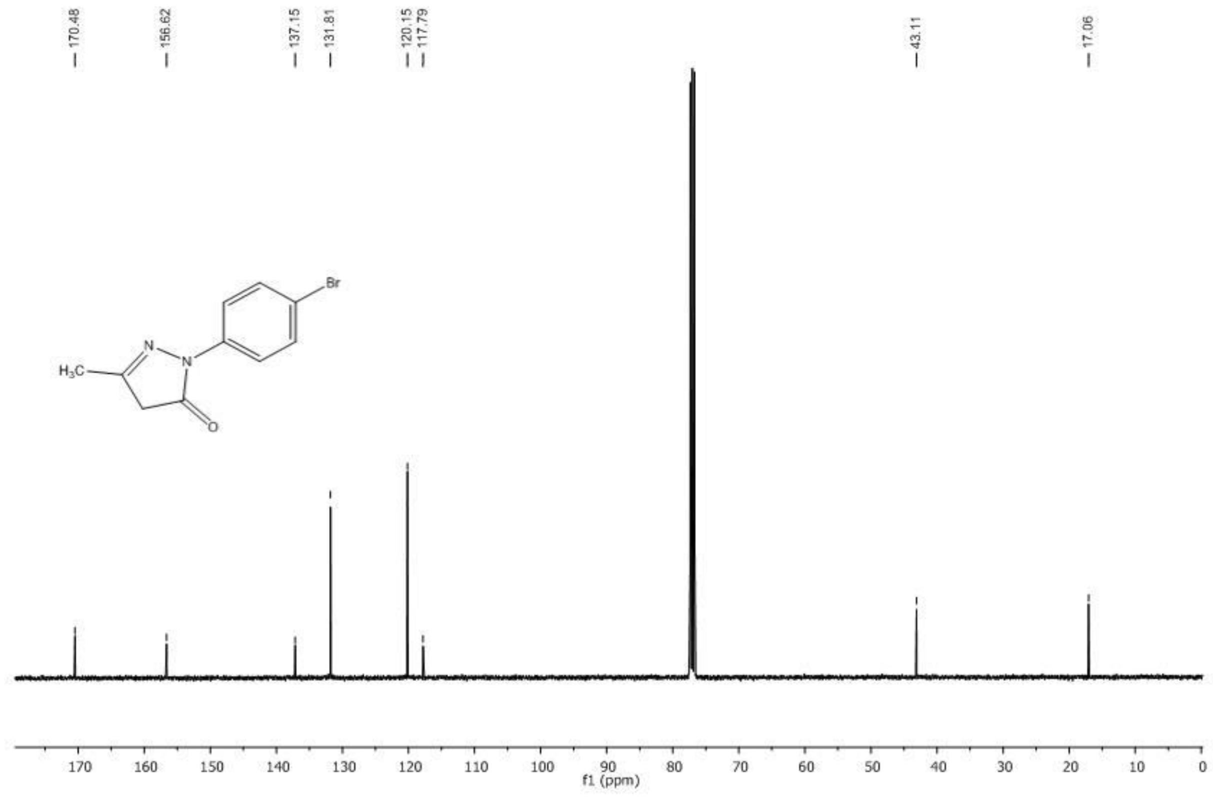

Figure S22. ^{13}C NMR spectrum in $CDCl_3$ of 1-(4-bromophenyl)-3-methyl-1*H*-pyrazole-5(4*H*)-one **3h**.

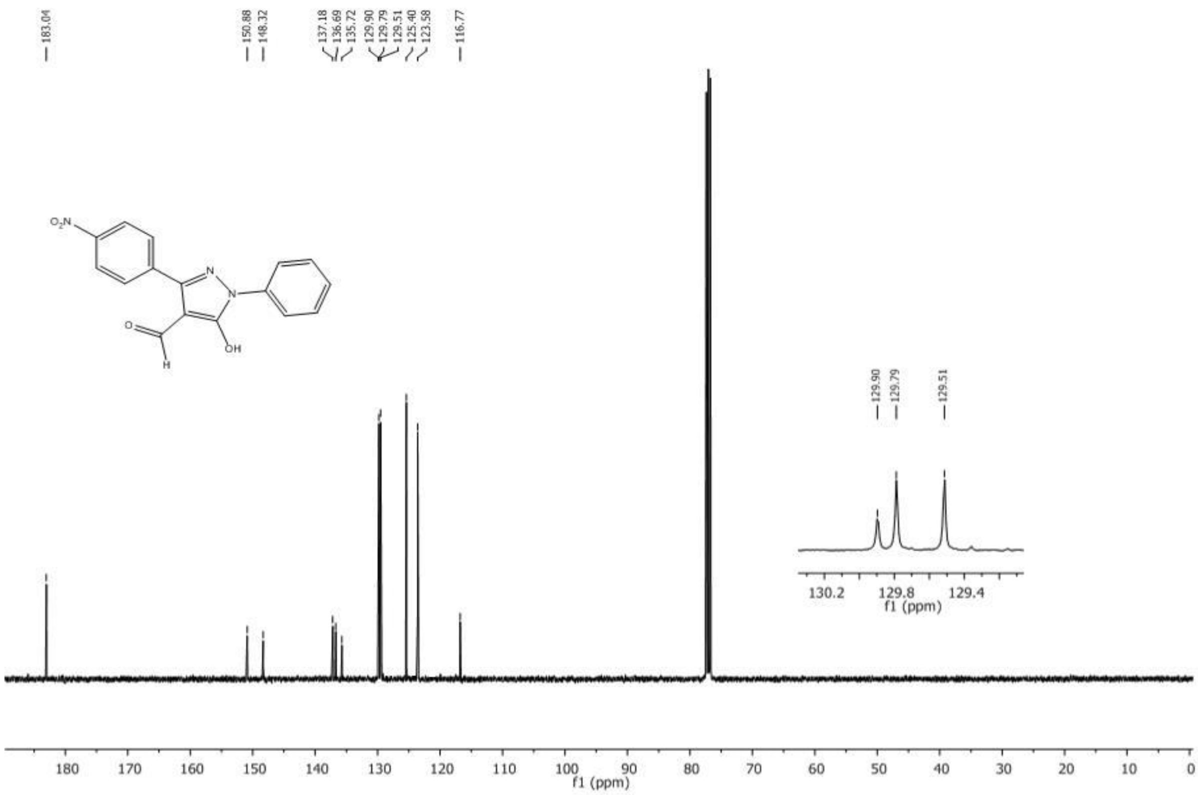

Figure S23. ^{13}C NMR spectrum in $CDCl_3$ of 5-hydroxy-3-(4-nitrophenyl)-1-phenyl-4-formyl-1*H*-pyrazole **4d**.

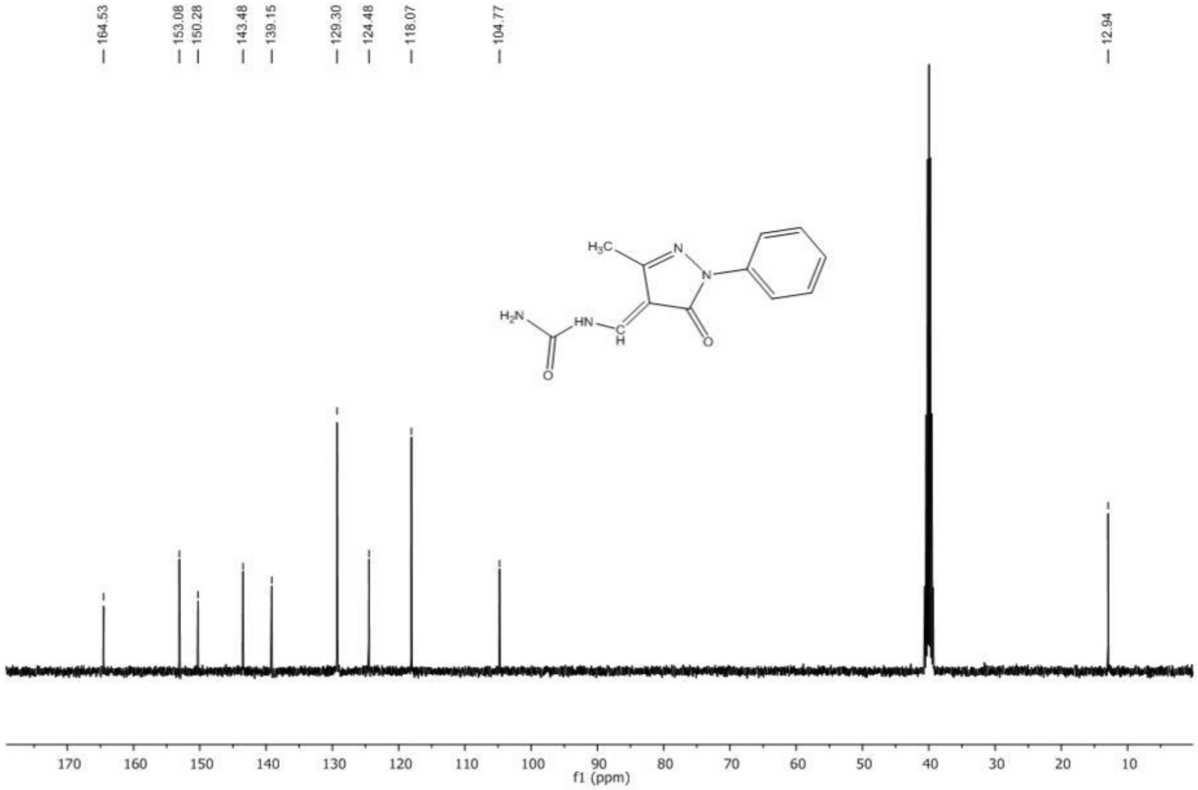

Figure S24. ^{13}C NMR spectrum in DMSO-d_6 of N-[(1,5-dihydro-3-methyl-5-oxo-1-phenyl-4*H*-pyrazole-4-ylidene)methyl]urea **5a**.

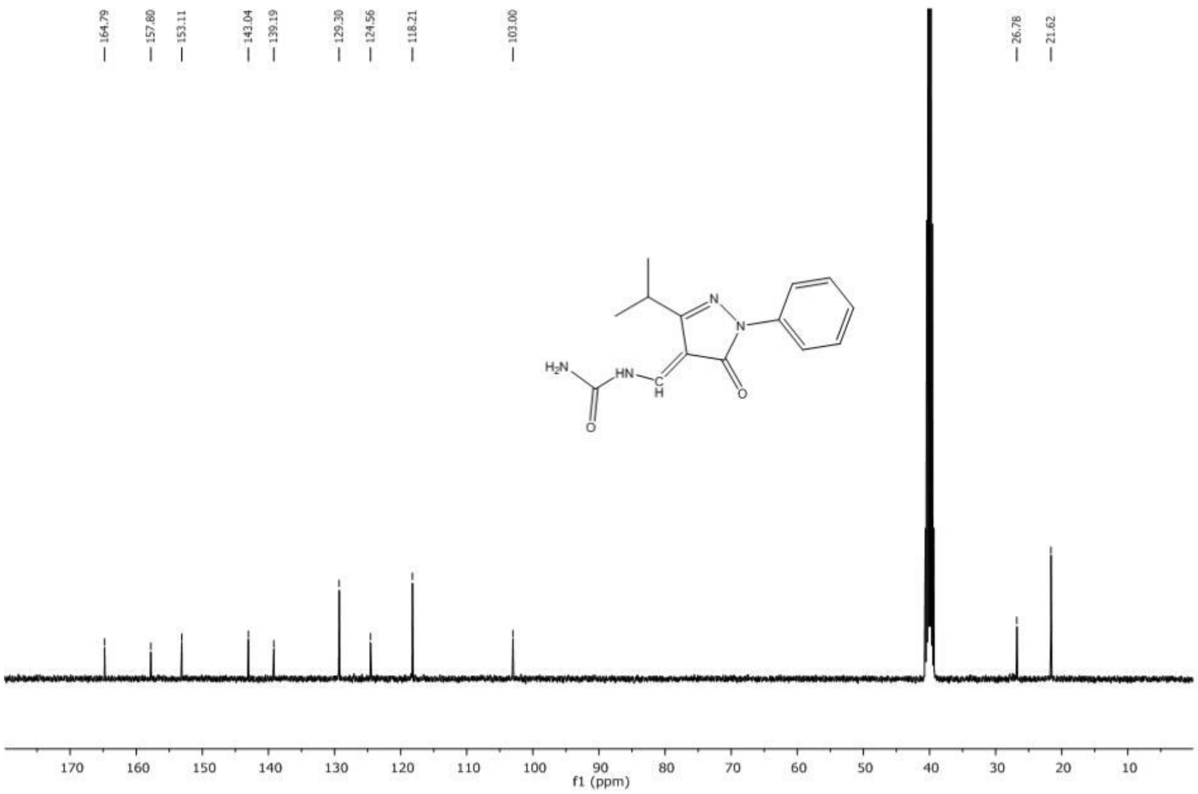

Figure S25. ^{13}C NMR spectrum in DMSO-d_6 of N-[(1,5-dihydro-1-phenyl-5-oxo-3-(isopropyl)-4*H*-pyrazole-4-ylidene)methyl]urea **5b**.

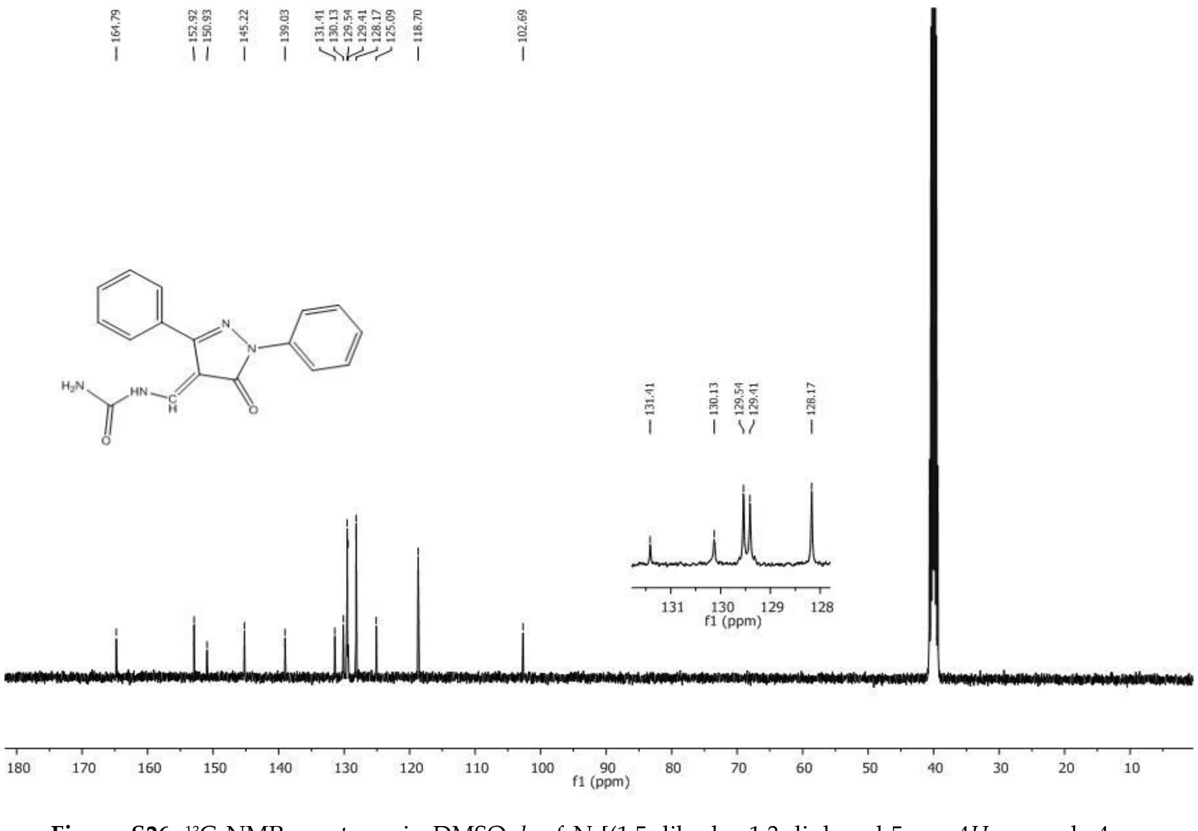

Figure S26. ^{13}C NMR spectrum in DMSO-d_6 of N-[(1,5-dihydro-1,3-diphenyl-5-oxo-4*H*-pyrazole-4-ylidene)methyl]urea **5c**.

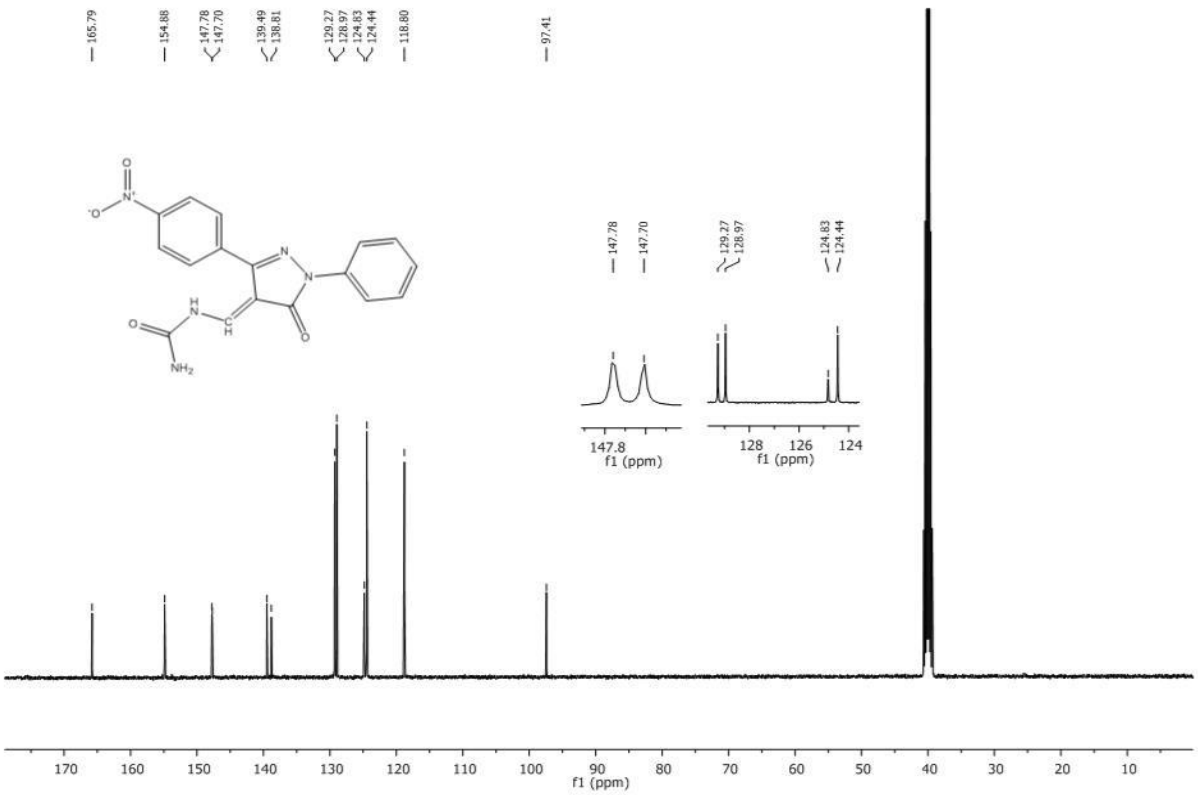

Figure S27. ^{13}C NMR spectrum in DMSO-d_6 of N-[(1,5-dihydro-1-phenyl-5-oxo-3-(4-nitrophenyl)-4*H*-pyrazole-4-ylidene)methyl]urea **5d**.

LC-MS/MS spectra

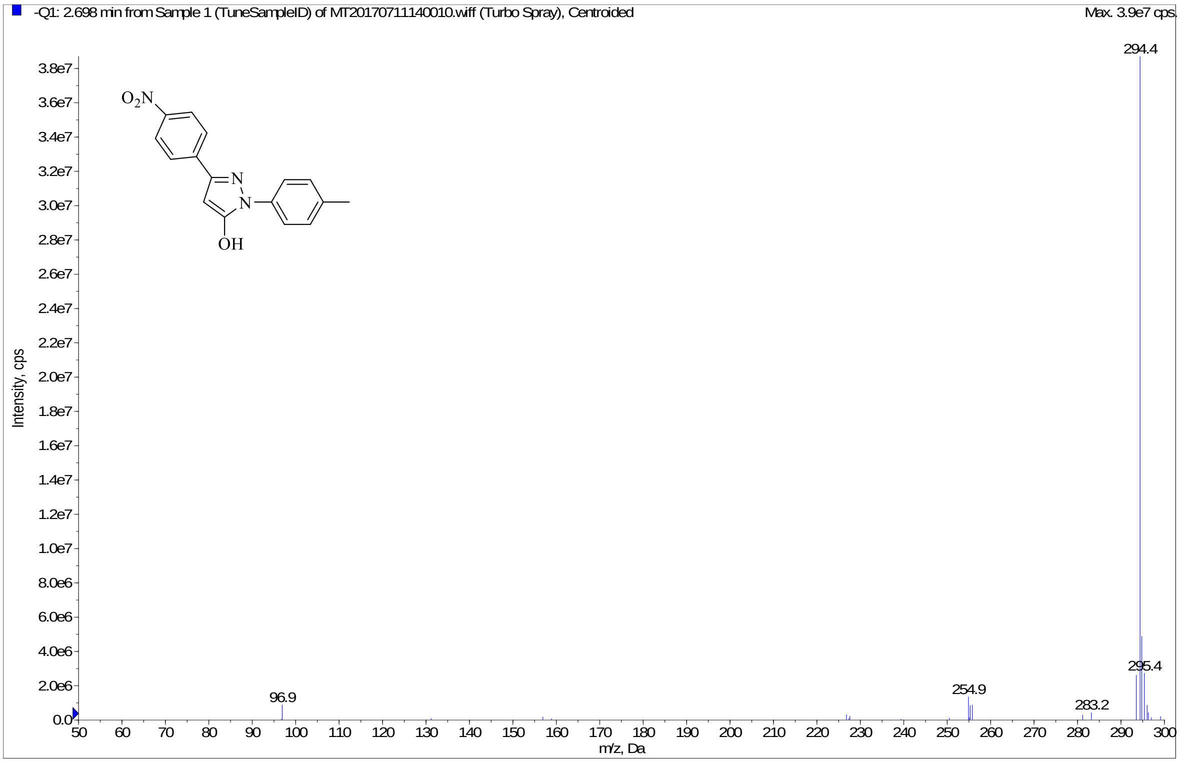

Figure S28. LC-MS/MS spectrum of 5-hydroxy-3-(4-nitrophenyl)-1-(p-tolyl)-1*H*-pyrazole **3e**.

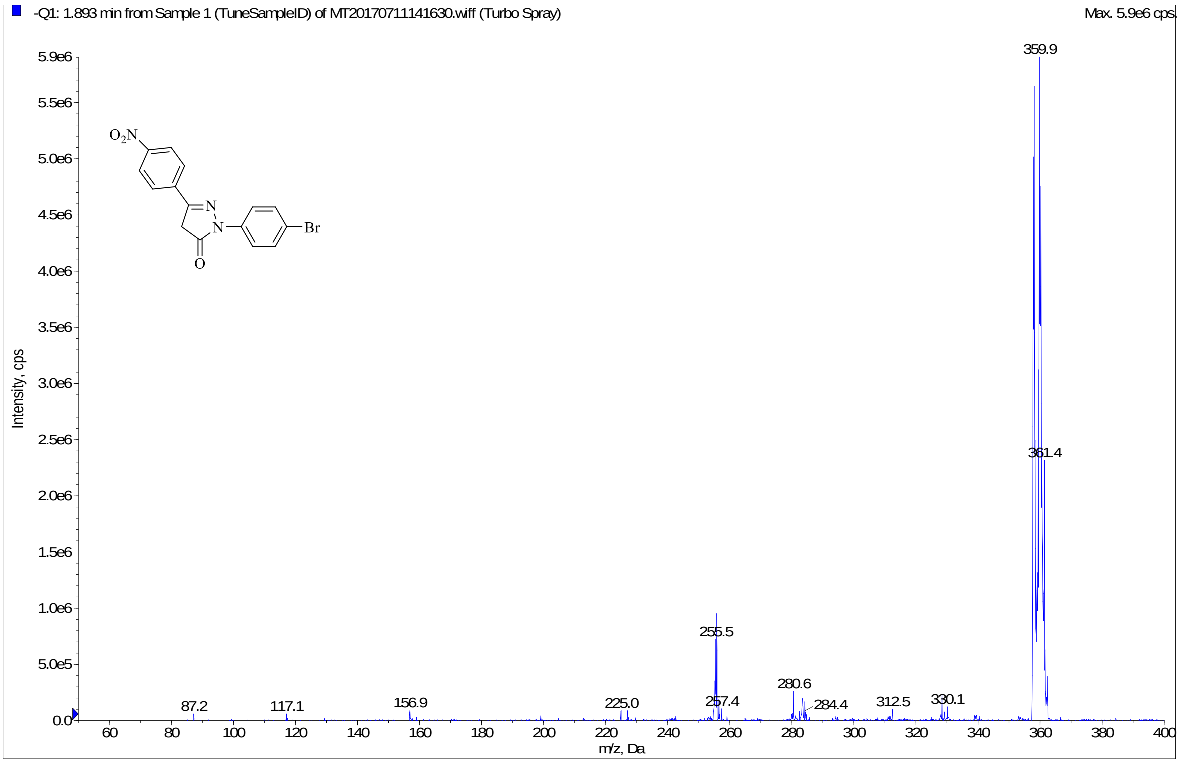

Figure S29. LC-MS/MS spectrum of 1-(4-bromophenyl)-3-(4-nitrophenyl)-1*H*-pyrazole-5(4*H*)-one **3f**.

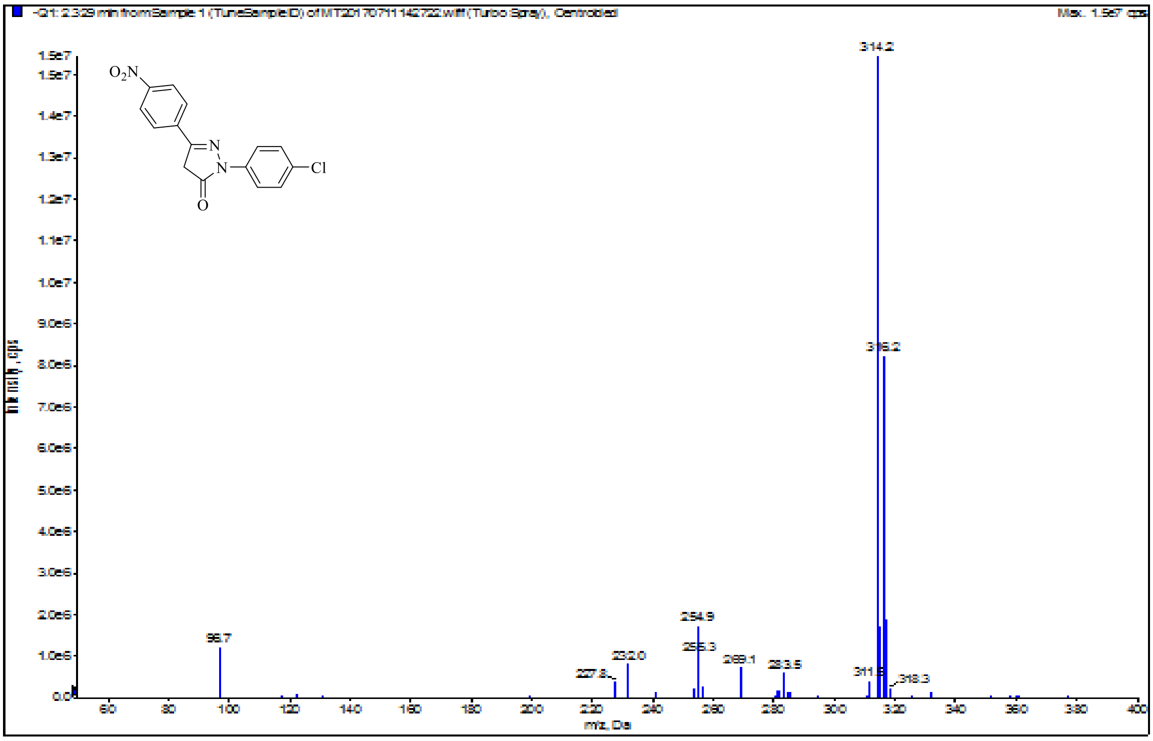

Figure S30. LC-MS/MS spectrum of 1-(4-chlorophenyl)-3-(4-nitrophenyl)-1*H*-pyrazole-5(4*H*)-one **3g**.

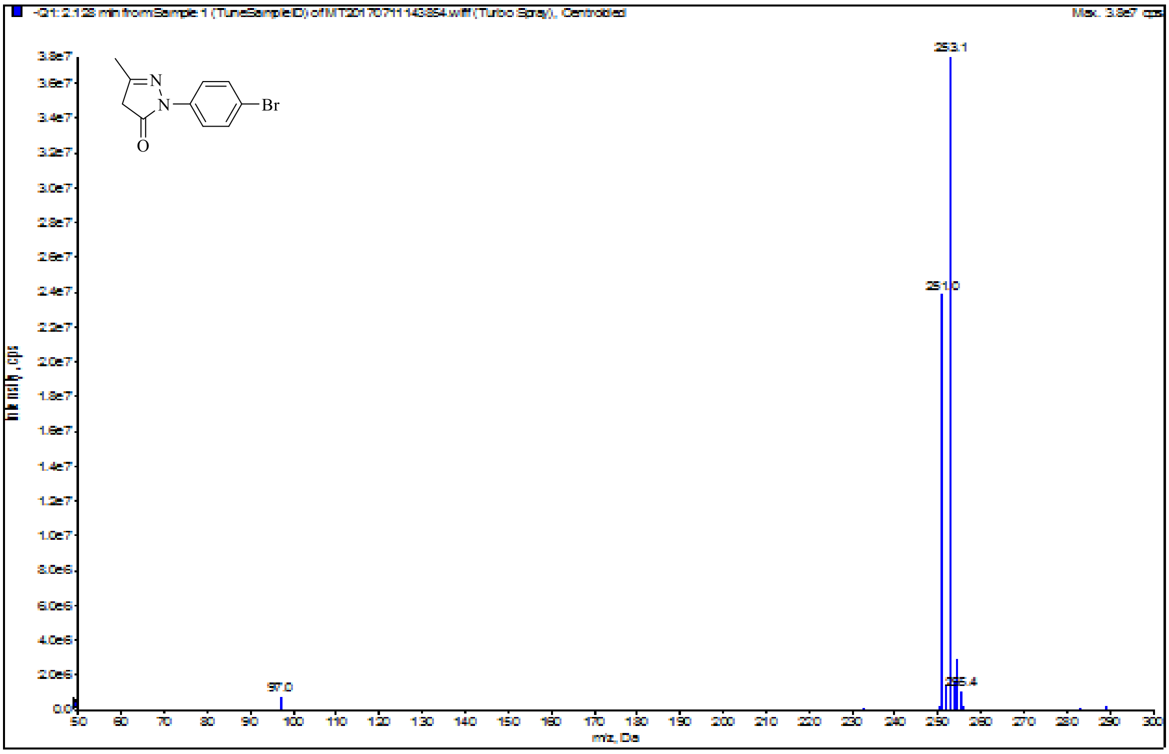

Figure S31. LC-MS/MS spectrum of 1-(4-bromophenyl)-3-methyl-1*H*-pyrazole-5(4*H*)-one **3h**.

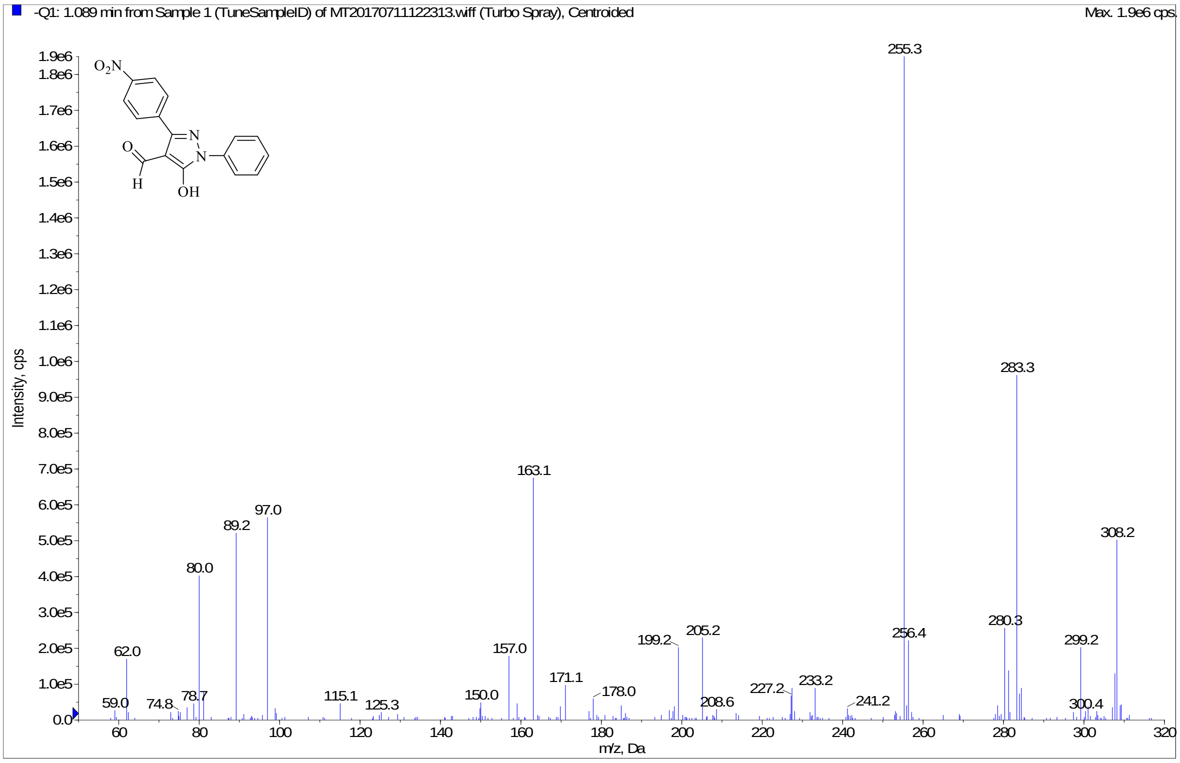

Figure S32. LC-MS/MS spectrum of 5-hydroxy-3-(4-nitrophenyl)-1-phenyl-4-formyl-1*H*-pyrazole **4d**.

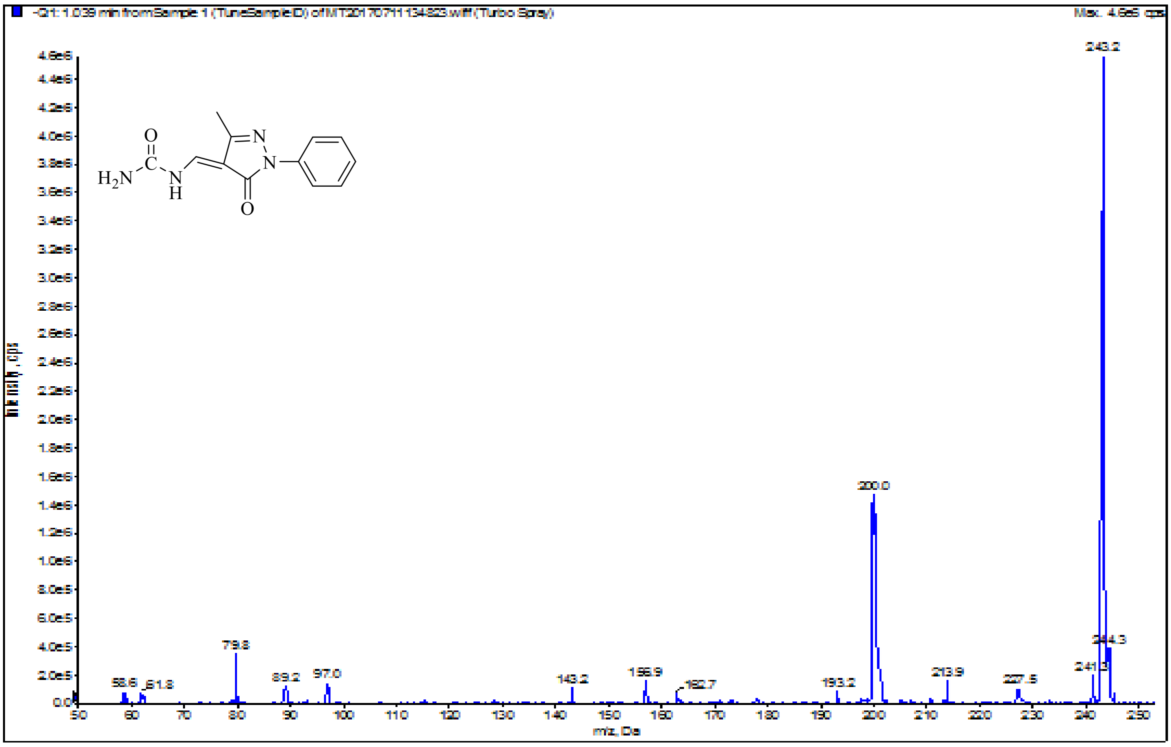

Figure S33. LC-MS/MS spectrum of N-[(1,5-dihydro-3-methyl-5-oxo-1-phenyl-4*H*-pyrazole-4-ylidene)methyl]urea **5a**.

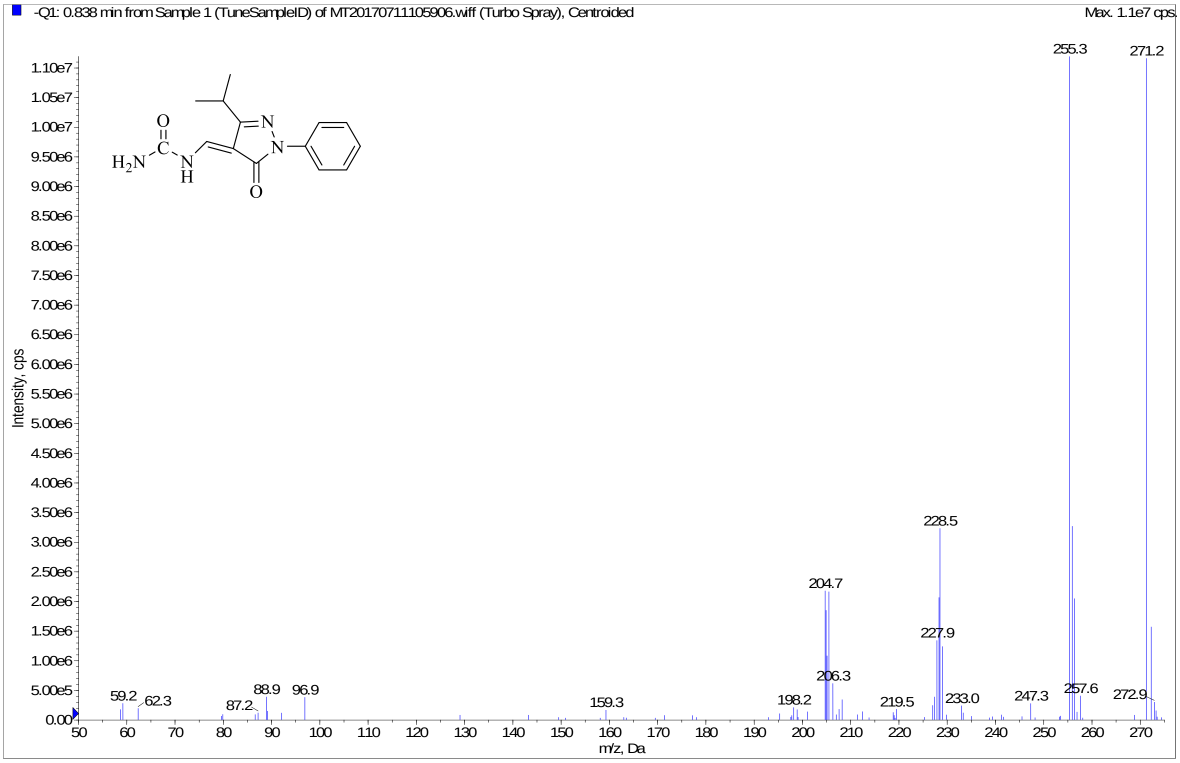

Figure S34. LC-MS/MS spectrum of N-[(1,5-dihydro-1-phenyl-5-oxo-3-(isopropyl)-4*H*-pyrazole-4-ylidene)methyl]urea **5b**.

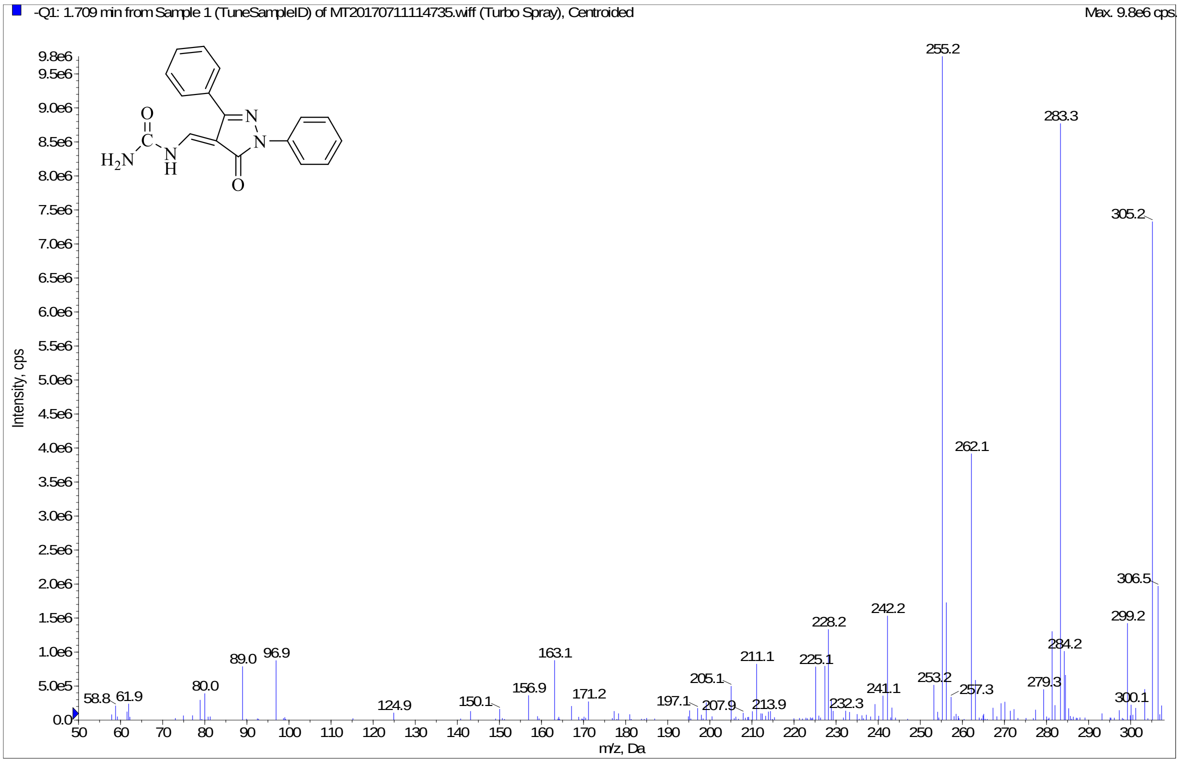

Figure S35. LC-MS/MS spectrum of N-[(1,5-dihydro-1,3-diphenyl-5-oxo-4*H*-pyrazole-4-ylidene)methyl]urea **5c**.

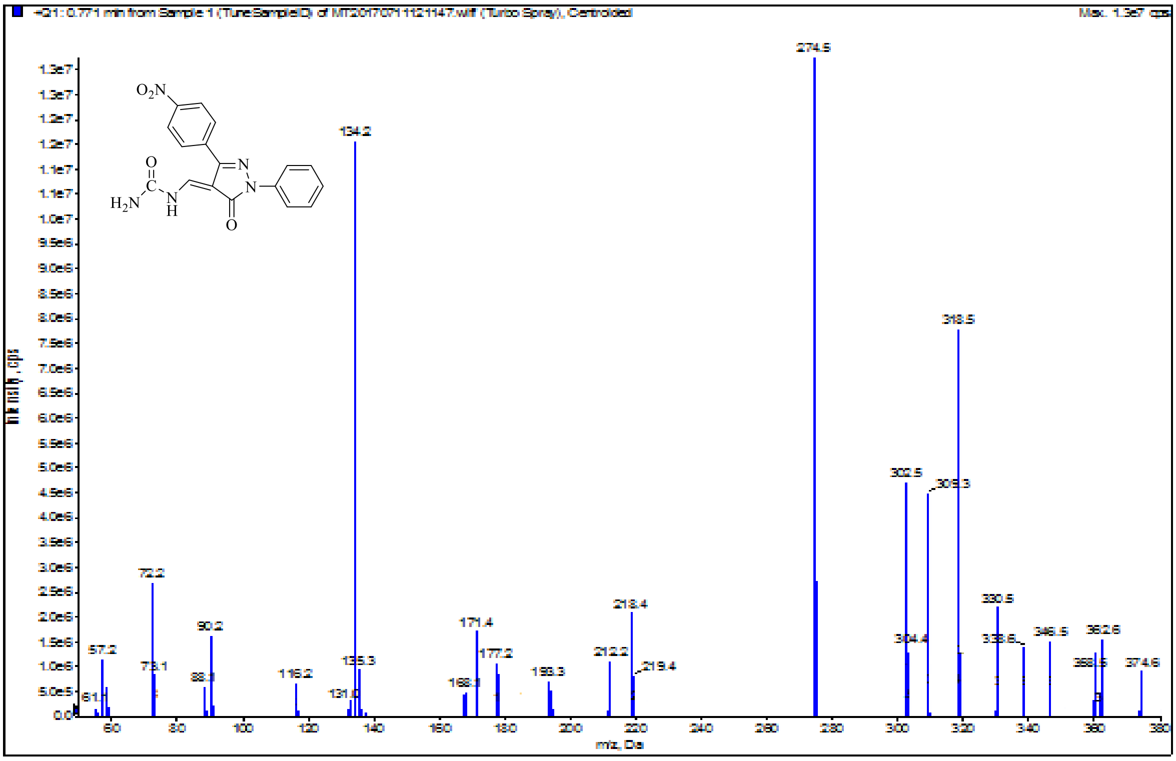

Figure S36. LC-MS/MS spectrum of N-[(1,5-dihydro-1-phenyl-5-oxo-3-(4-nitrophenyl)-4*H*-pyrazole-4-ylidene)methyl]urea **5d**.

Elemental analysis results

Table 1. Elemental analysis results of compounds.

No	Compounds	Formula	Results		
			Element	Calculated [%]	Found [%]
**3e**	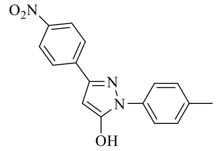	$C_{16}H_{13}N_3O_3$	C	65.08	64.97
			H	4.44	4.54
			N	14.23	14.17
**3f**	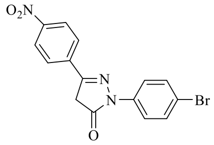	$C_{15}H_{10}BrN_3O_3$	C	50.02	49.86
			H	2.80	2.83
			N	11.67	11.55
**3g**	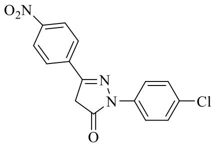	$C_{15}H_{10}ClN_3O_3$	C	57.07	56.79
			H	3.19	3.29
			N	13.31	13.87
**3h**	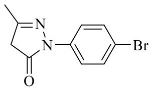	$C_{10}H_9BrN_2O$	C	47.46	47.54
			H	3.58	3.54
			N	11.07	11.14
**4d**	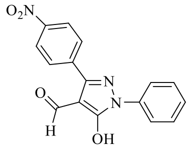	$C_{16}H_{11}N_3O_4$	C	62.14	62.06
			H	3.59	3.50
			N	13.59	13.65
**5a**	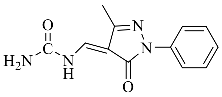	$C_{12}H_{12}N_4O_2$	C	59.01	58.93
			H	4.95	4.81
			N	22.94	23.07
**5b**	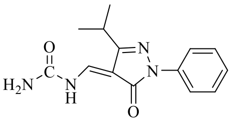	$C_{14}H_{16}N_4O_2$	C	61.75	61.88
			H	5.92	5.86
			N	20.58	20.77
**5c**	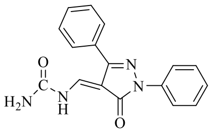	$C_{17}H_{14}N_4O_2$	C	66.66	66.9
			H	4.61	4.50
			N	18.29	18.50
**5d**	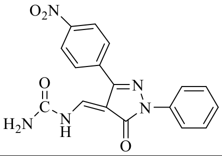	$C_{17}H_{13}N_5O_4$	C	58.12	58.26
			H	3.73	3.70
			N	19.93	20.16

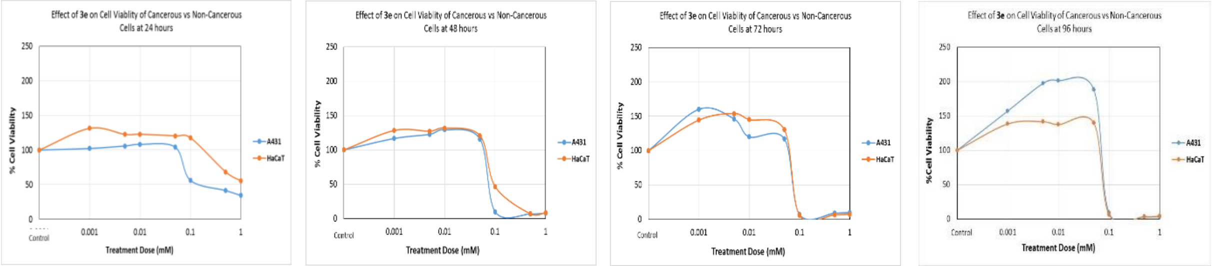

Figure S37. Effects of **3e** on Cell Viability of Cancerous vs Non-Cancerous Cells at 24, 48, 72, and 96 Hours.

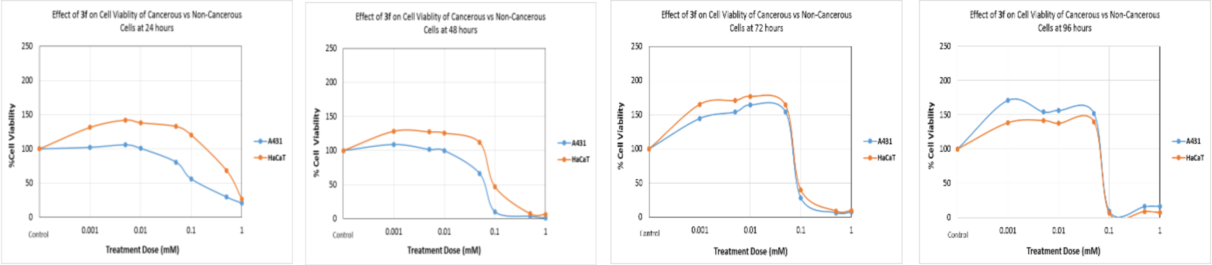

Figure S38. Effects of **3f** on Cell Viability of Cancerous vs Non-Cancerous Cells at 24, 48, 72, and 96 Hours.

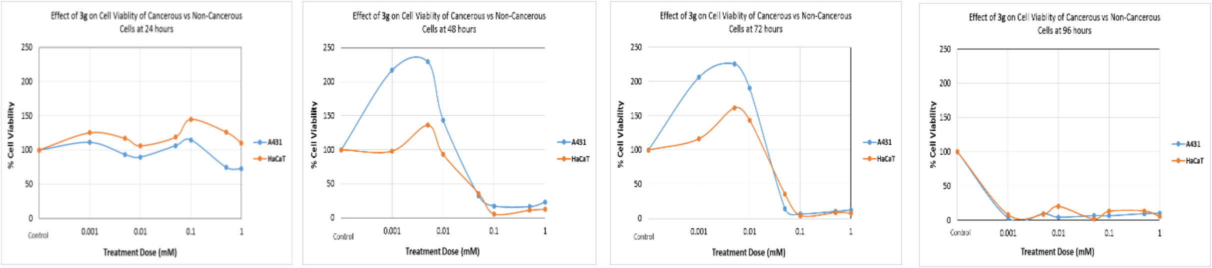

Figure S39. Effects of **3g** on Cell Viability of Cancerous vs Non-Cancerous Cells at 24, 48, 72, and 96 Hours.

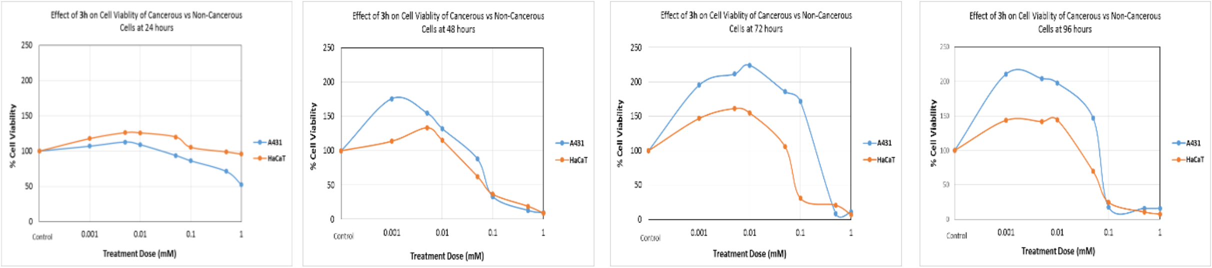

Figure S40. Effects of **3h** on Cell Viability of Cancerous vs Non-Cancerous Cells at 24, 48, 72, and 96 Hours.

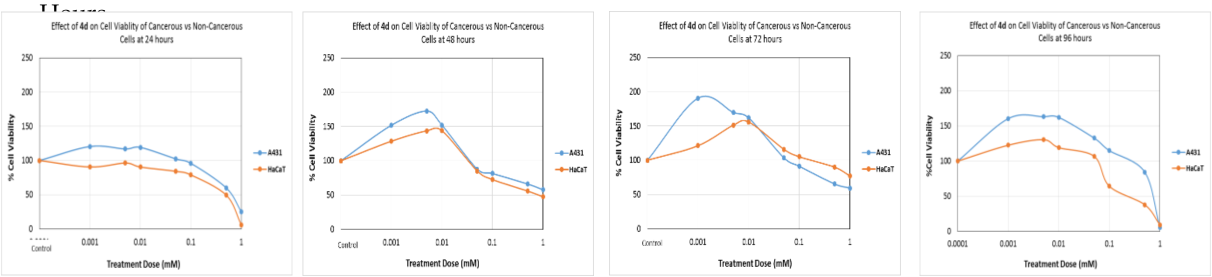

Figure S41. Effects of **4d** on Cell Viability of Cancerous vs Non-Cancerous Cells at 24, 48, 72, and 96 Hours.

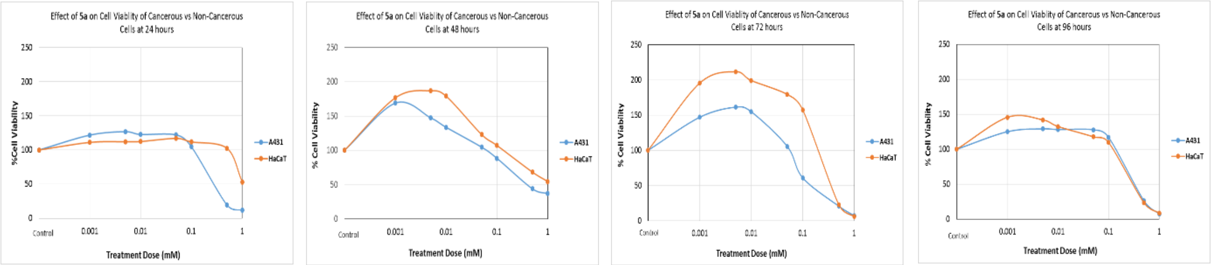

Figure S42. Effects of **5a** on Cell Viability of Cancerous vs Non-Cancerous Cells at 24, 48, 72, and 96 Hours.

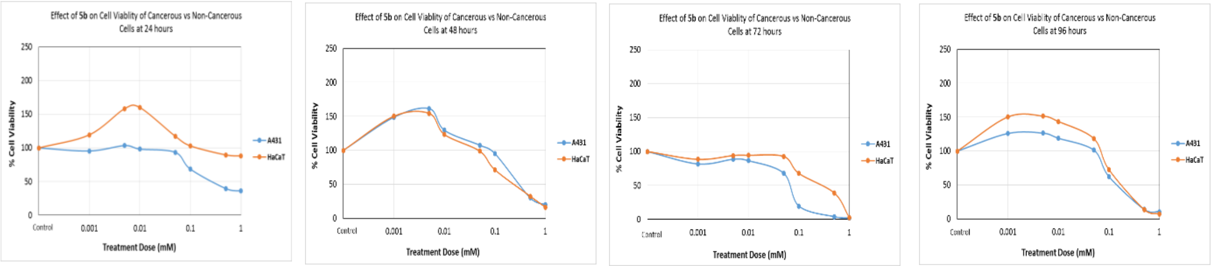

Figure S43. Effects of **5b** on Cell Viability of Cancerous vs Non-Cancerous Cells at 24, 48, 72, and 96 Hours.

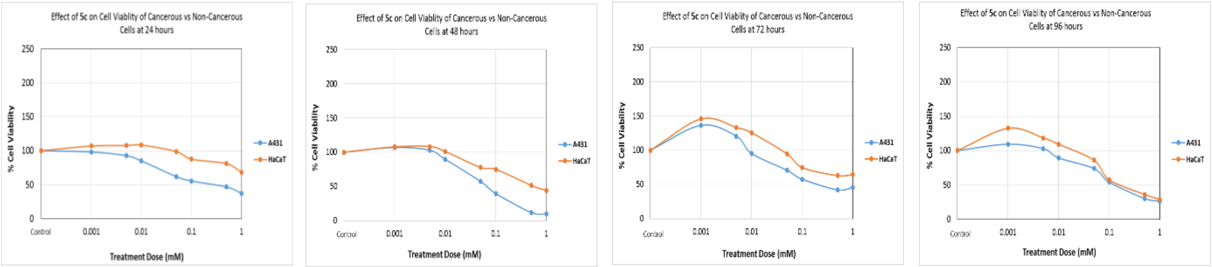

Figure S44. Effects of **5c** on Cell Viability of Cancerous vs Non-Cancerous Cells at 24, 48, 72, and 96 Hours.

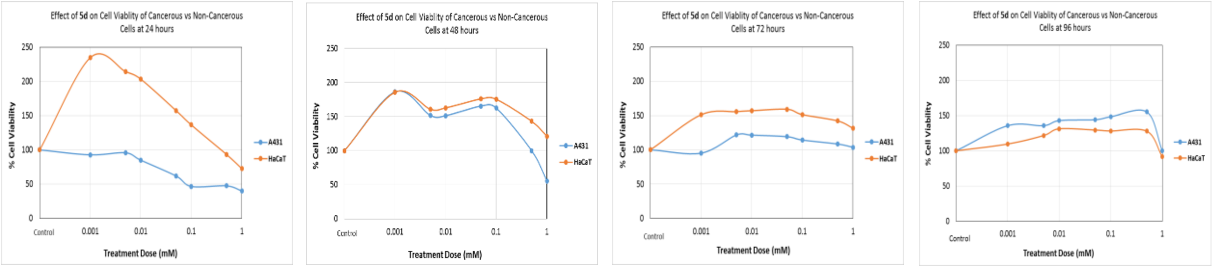

Figure S45. Effects of **5d** on Cell Viability of Cancerous vs Non-Cancerous Cells at 24, 48, 72, and 96 Hours.

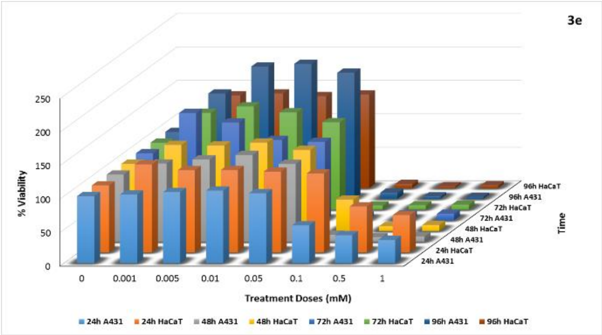
3e
% Viability
0
50
100
150
200
250
0 0.001 0.005 0.01 0.05 0.1 0.5 1
Treatment Doses (mM)
Time
24h A431 24h HaCaT 48h A431 48h HaCaT 72h A431 72h HaCaT 96h A431 96h HaCaT

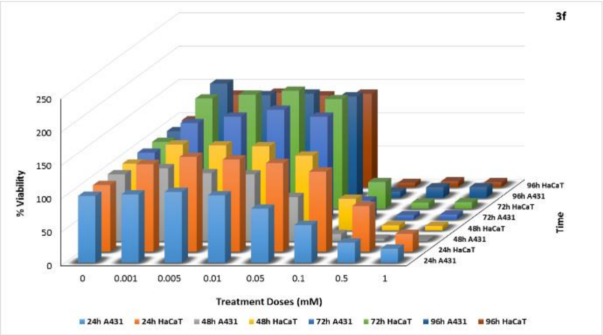
3f
% Viability
0
50
100
150
200
250
0 0.001 0.005 0.01 0.05 0.1 0.5 1
Treatment Doses (mM)
Time
24h A431 24h HaCaT 48h A431 48h HaCaT 72h A431 72h HaCaT 96h A431 96h HaCaT

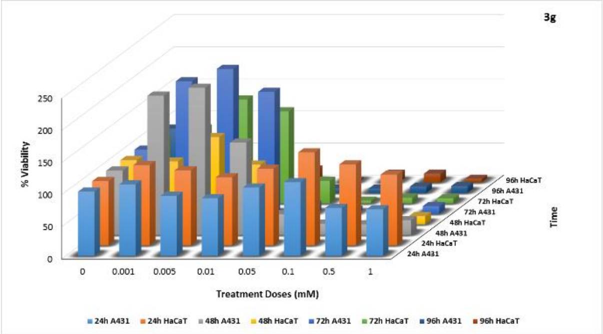
3g
% Viability
0
50
100
150
200
250
0 0.001 0.005 0.01 0.05 0.1 0.5 1
Treatment Doses (mM)
Time
24h A431 24h HaCaT 48h A431 48h HaCaT 72h A431 72h HaCaT 96h A431 96h HaCaT

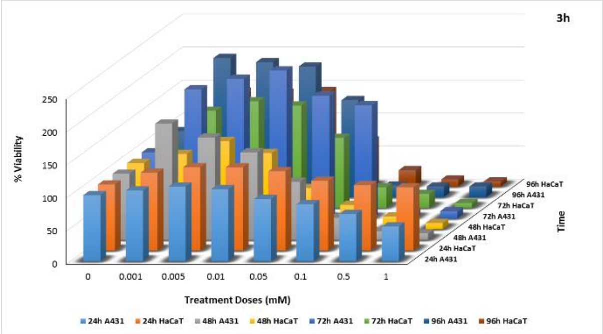
3h
% Viability
0
50
100
150
200
250
0 0.001 0.005 0.01 0.05 0.1 0.5 1
Treatment Doses (mM)
Time
24h A431 24h HaCaT 48h A431 48h HaCaT 72h A431 72h HaCaT 96h A431 96h HaCaT

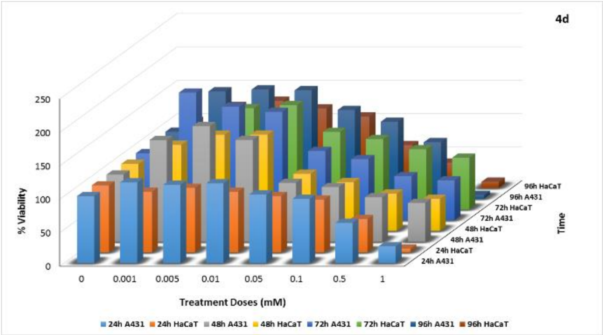
4d
% Viability
0
50
100
150
200
250
0 0.001 0.005 0.01 0.05 0.1 0.5 1
Treatment Doses (mM)
Time
24h A431 24h HaCaT 48h A431 48h HaCaT 72h A431 72h HaCaT 96h A431 96h HaCaT

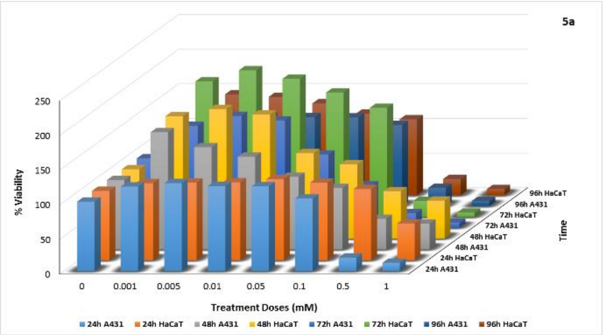
5a
% Viability
0
50
100
150
200
250
0 0.001 0.005 0.01 0.05 0.1 0.5 1
Treatment Doses (mM)
Time
24h A431 24h HaCaT 48h A431 48h HaCaT 72h A431 72h HaCaT 96h A431 96h HaCaT

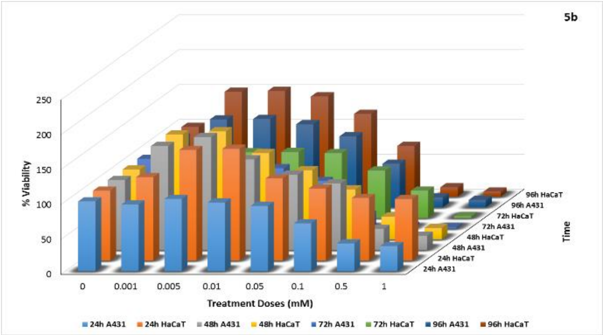
5b
% Viability
0
50
100
150
200
250
0 0.001 0.005 0.01 0.05 0.1 0.5 1
Treatment Doses (mM)
Time
24h A431 24h HaCaT 48h A431 48h HaCaT 72h A431 72h HaCaT 96h A431 96h HaCaT

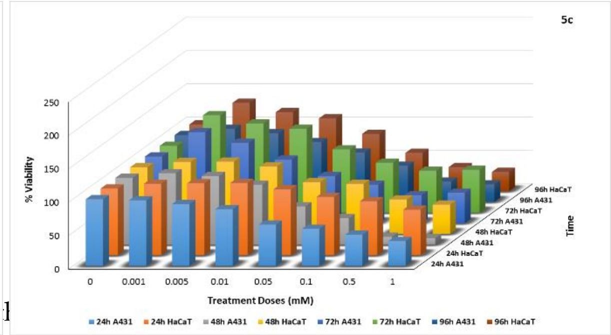
5c
% Viability
0
50
100
150
200
250
0 0.001 0.005 0.01 0.05 0.1 0.5 1
Treatment Doses (mM)
Time
24h A431 24h HaCaT 48h A431 48h HaCaT 72h A431 72h HaCaT 96h A431 96h HaCaT

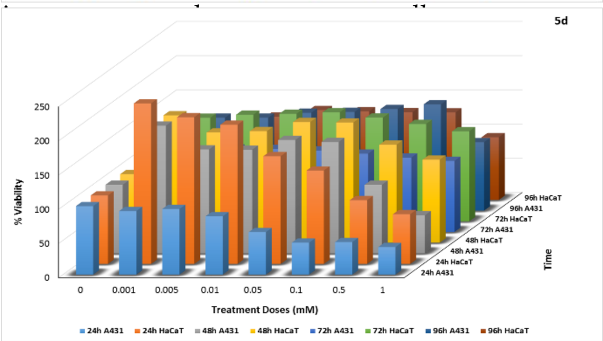
5d
% Viability
0
50
100
150
200
250
0 0.001 0.005 0.01 0.05 0.1 0.5 1
Treatment Doses (mM)
Time
24h A431 24h HaCaT 48h A431 48h HaCaT 72h A431 72h HaCaT 96h A431 96h HaCaT
